# Facial emotion recognition deficits in bipolar disorder: A systematic review and meta-analysis

**DOI:** 10.1192/j.eurpsy.2025.10147

**Published:** 2026-01-12

**Authors:** Michele De Prisco, Vincenzo Oliva, Chiara Possidente, Giovanna Fico, Laura Montejo, Lydia Fortea, Hanne Lie Kjærstad, Kamilla Woznica Miskowiak, Gerard Anmella, Diego Hidalgo-Mazzei, Alessandro Miola, Michele Fornaro, Andrea Murru, Eduard Vieta, Joaquim Radua

**Affiliations:** 1 Department of Medicine, Faculty of Medicine and Health Sciences, Institute of Neurosciences (UBNeuro), University of Barcelona (UB), C. Casanova, 143, 08036 Barcelona, Catalonia, Spain; 2 Bipolar and Depressive Disorders Unit, Hospìtal Clinic de Barcelona. c. Villarroel, 170, 08036 Barcelona, Catalonia, Spain; 3 Institut d’Investigacions Biomèdiques August Pi i Sunyer (IDIBAPS), c. Villarroel, 170, 08036 Barcelona, Catalonia, Spain; 4 Centro de Investigación Biomédica en Red de Salud Mental (CIBERSAM), Instituto de Salud Carlos III, Madrid, Spain; 5 Department of Biomedical and Neuromotor Sciences, University of Bologna, Bologna, Italy; 6 Imaging of Mood- and Anxiety-Related Disorders (IMARD) Group, Barcelona, Catalonia, Spain; 7 Neurocognition and Emotion in Affective Disorder Centre (NEAD), Psychiatric Centre Copenhagen, Frederiksberg Hospital, Denmark; 8 Department of Psychology, University of Copenhagen, Denmark; 9 Department of Neuroscience, University of Padova, Padua, Italy; 10 Department Psychiatry and Psychology, Mayo Clinic, Rochester, MN, USA; 11 Section of Psychiatry, Department of Neuroscience, Reproductive Science and Odontostomatology Federico II University of Naples, Naples, Italy

**Keywords:** affective cognition, bipolar disorder, facial emotion recognition, meta-analysis, review

## Abstract

**Background:**

Bipolar disorder (BD) is associated with impairments in facial emotion recognition (FER), affecting social functioning and quality of life. Understanding FER deficits in BD is crucial for tailoring interventions and improving treatment outcomes. This systematic review and meta-analysis aims to evaluate FER differences among individuals with BD, unaffected first-degree relatives (FDRs), and healthy controls (HCs), exploring predictors related to patient and study characteristics.

**Methods:**

We systematically searched PubMed/MEDLINE, Scopus, EMBASE, and PsycINFO databases from inception to March 28, 2024. Random-effects meta-analyses were conducted to explore differences in accuracy and reaction time during FER identification and discrimination tasks.

**Results:**

A total of 100 studies were included, comprising 4920 individuals with BD (females = 56%, mean age = 34.1 ± 9.1), 676 FDRs (females = 55%, mean age = 36.1 ± 12), and 4909 HCs (females = 53.2%, mean age = 32.5 ± 9.5). Compared to HCs, adults with BD exhibited significantly lower accuracy (SMD = −0.47; 95% CIs = −0.56, −0.38) and higher reaction time (SMD = 0.57; 95%CIs = 0.33, 0.81) during facial emotion identification tasks. During facial emotion discrimination tasks, adults with BD had significantly lower accuracy than HCs (SMD = −0.59; 95%CIs = −0.78, −0.4), but similar speed. No significant differences were observed between BD and FDRs. Meta-regressions identified several predictors of FER performance, including manic symptom severity, stimulus duration, and presence of practice before task.

**Conclusions:**

FER deficits appear to be a core feature of BD and require specialized, systematic assessment. Identifying these deficits may help guide interventions aimed at improving affective cognition and social outcomes in individuals with BD.

## Introduction

Bipolar disorder (BD) is a severe mental illness characterized by significant fluctuations in emotions, energy levels, and thoughts, affecting up to 1.1% of the world’s population [1]. In addition to mood symptoms, individuals with BD often experience cognitive impairments. Cognition broadly includes both neurocognition, which incorporates core mental processes such as memory, attention, and executive functions, and affective cognition (AC), which involves functions related to perceiving, interpreting, and responding to emotional stimuli, representing an integration of emotional and neurocognitive processes [2]. These domains collectively shape how individuals perceive, process, and respond to the world around them. Understanding these aspects is particularly important in conditions like BD, since impairments in these domains can profoundly impact daily functioning, interpersonal relationships, and overall quality of life [3]. While neurocognitive impairments have been extensively studied and recognized in BD [4], there has been a growing interest in AC as a key area of investigation, due to its crucial role in everyday interactions, contributing to social adaptation and integration [5]. AC incorporates various interconnected domains, including emotional intelligence, emotional decision-making, reward and punishment processing, emotion regulation, and facial emotion recognition (FER) [6]. Individuals with BD exhibit several alterations across these domains, including impaired emotional intelligence [7], disrupted decision making [8], and difficulties in emotion regulation [9]. Among these components of AC, FER is the ability to identify and discriminate the emotional meaning of specific facial expressions displayed by other people, typically categorized into six basic and discrete emotions (i.e., anger, disgust, fear, happiness, sadness, and surprise) [10]. FER serves as a bridge between cognitive and emotional processes, and it is critical for interacting and communicating with others, allowing individuals to make appropriate cognitive and behavioral adaptations during interpersonal exchanges [5]. Deficits in FER can have a critical impact on people’s ability to function in daily life, including in work and in social settings [11]. The International Society of Bipolar Disorder Targeting Cognition Task Force [6] emphasized the significance of understanding deficits in AC, including FER, in individuals with BD. This understanding is essential for uncovering the neurobiological basis of these impairments and for developing targeted treatments that go beyond symptom control, to improve patient-centered outcomes, including functioning, well-being, and overall quality of life. To quantify the magnitude of FER impairments in BD, we previously conducted a systematic review and meta-analysis [12] comparing patients with BD with individuals with other psychiatric disorders. Our findings revealed that individuals with BD showed a better FER performance compared to those with schizophrenia, but performed worse than individuals with major depressive disorder.

Currently, there is a lack of meta-analytic evidence to quantify whether and how FER differs between BD patients, unaffected first-degree relatives (FDRs), and healthy controls (HCs), relevant for guiding future research and clinical practice. Therefore, this systematic review and meta-analysis aims to (a) investigate FER performance in BD compared with FDRs and HCs, and (b) identify potential predictors that might influence FER performance.

## Methods

This systematic review and meta-analysis were conducted according to the Meta-analysis Of Observational Studies in Epidemiology (MOOSE) guidelines [13]. The protocol was registered on PROSPERO (CRD42023422035). The MOOSE checklist and deviations from the protocol are reported in Supplementary Appendix I.

### Identification and selection of studies

The PubMed/MEDLINE, Scopus, EMBASE, and PsycINFO databases were searched from inception to March 28, 2024. Search strategies are provided in Supplementary Appendix II. Reference lists of each included study, textbooks, and other materials were searched to identify additional studies. Inclusion criteria required original studies (a) to provide quantitative data on FER, (b) in people diagnosed with BD (c) according to the Diagnostic and Statistical Manual for Mental Disorders or the International Classification of Diseases diagnostic criteria, (d) and compared with unaffected FDRs (i.e., without BD) or HCs. Both observational and interventional studies were considered, but only baseline data were collected. No age, sample size, or language restrictions were applied. Where populations overlapped across multiple studies, the largest study with the most representative data, or the most recent, was preferred. Exclusion criteria included (a) reviews, (b) case reports, (c) case series, (d) studies not peer-reviewed, (e) studies that were retracted after publication, and (f) animal studies. Three authors (MDP, VO, CP) independently examined studies of potential interest at both title and abstract and full-text screening, and in case of disagreement, another author (JR) was consulted. The same three authors independently extracted relevant data regarding outcome measures and study characteristics. In cases of insufficient data, corresponding authors were contacted twice.

### Risk of bias

Three authors (MDP, VO, CP) independently evaluated the risk of bias of included studies using the Newcastle-Ottawa Scale (NOS) [14], and another author (JR) resolved disagreements. The NOS scores were converted to Agency for Healthcare Research and Quality (AHRQ) standards, as done elsewhere [12].

### Outcomes

FER tasks focusing on emotion identification (i.e., the ability to match an emotional stimulus with its corresponding emotion) or discrimination (i.e., the ability to discriminate whether two presented faces show the same emotion or not) were studied. Any outcome aimed at exploring the differences in FER was considered eligible for inclusion. “Accuracy” was defined as any measure assessing the overall correct recognition of emotions (e.g., percentage of correct recognition, scores from specific scales evaluating the number of emotions correctly identified, and author-defined accuracy). “Reaction time” was defined as the time elapsed before responding, and “number of errors” referred to the count of errors made by individuals during a FER task.

### Statistical analysis

Random-effect meta-analyses (restricted maximum-likelihood estimator) [15] were conducted through the R-package “metafor” [16], using R, version 4.3.1 [17]. The results were presented for each outcome according to a three-level scheme, as done in our previous studies [12, 18]. Level one represents a global measure of FER, referring to the overall ability to recognize any emotion. Level two is a measure of FER based on emotion valence, distinguishing between negative (i.e., anger, disgust, fear, or sadness), or positive (i.e., happiness or surprise) emotions. Level three is a measure of FER based on specific emotions analyzed separately (i.e., anger, disgust, fear, happiness, sadness, or surprise). A graphical representation of this three-level scheme is presented in Supplementary Appendix II. When studies did not provide information at all levels, we hierarchically derived missing levels to maximize data inclusion, where possible. Specifically: (a) when studies provided only level-three data (i.e., relative to specific emotions), we computed mean values for emotions belonging to the same valence (i.e., negative or positive) to obtain level-two information; (b) when studies reported level-three data without an overall measure of FER, we computed mean values across all available individual emotions to obtain level-one information; (c) when studies reported only level-two data (i.e., positive or negative emotion scores), we computed mean values across them to derive level-one information. Standardized mean differences (SMD) with their confidence intervals (CIs) were used as effect sizes and represented by Hedge’s g. These values can be interpreted as indicating small (SMD = 0.2), moderate (SMD = 0.5), or large (SMD = 0.8) effects, based on their magnitude [19]. Heterogeneity was assessed by using Cochran’s Q test [20], *τ* ^2^ and *I* ^2^ statistics [21]. Prediction intervals were calculated. For the associations analyzed at level one, meta-regression analyses were conducted according to the following predictors considering original study characteristics (i.e., primary or secondary outcome, and publication year), sociodemographic (i.e., % of females, mean age, and years of education), clinical characteristics of people with BD (i.e., % of people in (hypomania), % of people in depression, % of people in euthymia, % of BD-I, age at onset, duration of illness, depression symptoms severity, and manic symptoms severity), and FER task characteristics (i.e., duration of the stimulus, duration of the inter-stimulus interval, maximum emotion intensity, minimum emotion intensity, presence of morphing, number of emotions considered in the whole task, number of stimuli, presence of practice, and presence of neutral faces), when at least 10 studies providing this data were available. For the associations analyzed at the levels two and three, the same meta-regression analyses were conducted when the Cochran’s *Q* test presented a *p* < 0.10 or the *I* ^2^ statistic showed a value >50%, and when at least 10 studies providing this data were available. Sensitivity analyses were conducted to explore the robustness of the results: (a) excluding one study at a time from the main analysis (i.e., leave-one-out); (b) by including only good-quality studies according to AHRQ standards; (c) by removing those studies whose lower-level data were calculated from higher-level information (only for levels one and two). Publication bias was explored by visual inspection of funnel plots and using the Egger’s test [22] when at least 10 studies were available.

## Results

**Figure 1.**
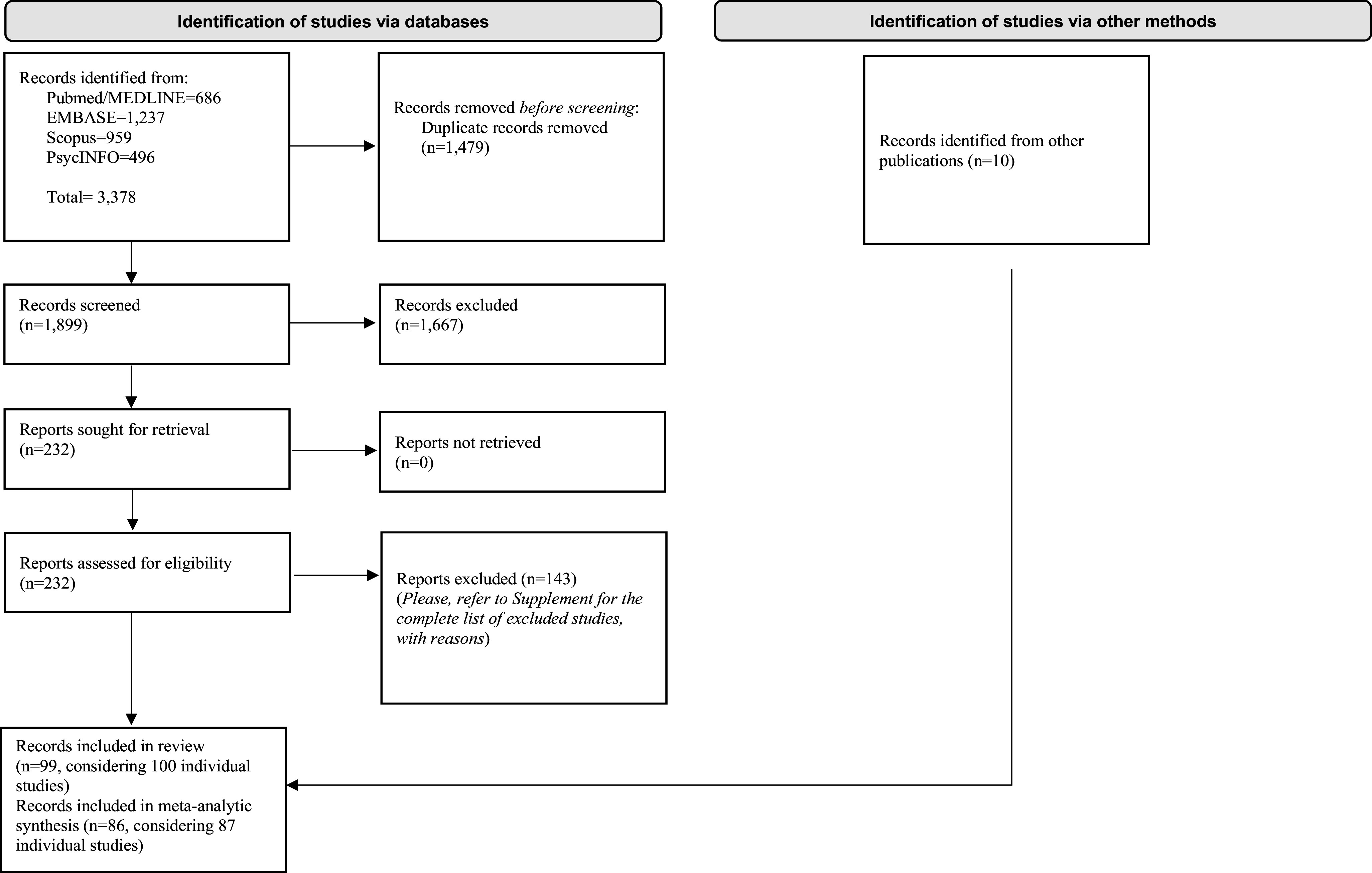
Flow diagram of study selection.

Overall, 3388 records were identified. After duplicate removal, 1667 were excluded at the title/abstract level and 143 after the full-text evaluation. Finally, 99 papers (considering 100 individual studies) were included, and 86 of them (considering 87 individual studies) provided enough data to perform a meta-analysis comparing people with BD to FDRs or HCs. The flow diagram is reported in Figure 1. Details on included studies are displayed in Table 1. Excluded studies, as well as additional information about the studies included only in the systematic review, are presented in Supplementary Appendices III-IV.

**Table 1.** Characteristics of the studies included in the systematic review and meta-analysis

Author, year, country	Study design	Setting	Population (*n*)	Mood state patients with BD (%)	Mean age	Percentage of females	Diagnostic criteria	Instrument adopted	Emotion type	Outcome type	Quality of the study (NOS)	Included in the meta-analysis
Addington et al., 1998 [47], Canada	Prospective cohort	Outpatients	BD (40) HCs (40)	Euthymic (97.5%) Depressed (2.5%)	38.5 ± 11 32.6 ± 11.3	75% 42.5%	DSM-III-R (SCID)	POFA	Anger, disgust, fear, happiness, sadness, surprise	Identification (score); Discrimination (score)	6 (POOR)	Yes
Almeida et al., 2010 [48], USA	Cross-sectional	NA	BD (30) HCs (15)	Euthymic (50%) Depressed (50%)	34.92 ± 9.85 32.69 ± 8	80% 80%	DSM-IV (SCID-P)	POFA, FEEST	Fear, happiness, sadness	Identification (accuracy)	7 (GOOD)	Yes
Altamura et al., 2016 [49], Italy	Cross-sectional	NA	BD (16) HCs (20)	Euthymic (100%)	46.2 ± 10.6 24 ± 4.1	56% 50%	DSM-IV (SCID)	KDEF	Anger, happiness	Identification (accuracy, reaction time)	6 (GOOD)	Yes
Baez et al., 2013 [50], Argentina	Cross-sectional	Outpatients	BD (15) HCs (30)	Euthymic (70%) Depressed (30%)	35.9 ± 11.8 34.3 ± 9.3	70% 50%	DSM-IV	POFA	Anger, disgust, fear, happiness, sadness, surprise	Identification (accuracy, response time)	6 (FAIR)	Yes
Barbosa et al., 2023 [51], Brazil	Cross-sectional	NA	BD (20) HCs (40)	Euthymic (100%)	57.4 ± 11.4 61.2 ± 9.9	60% 70%	DSM-IV (MINI)	POFA, Mini-SEA	Anger, disgust, fear, happiness, sadness, surprise	Identification (score)	7 (GOOD)	Yes
Bellack et al., 1996 [52], USA	Cross-sectional	Inpatients	BD (11) HCs (19)	NA	39.27 ± 5.75 34.68 ± 9.73	64% 58%	DSM-III-R (SCID-P)	POFA, FOE	Anger, disgust, fear, happiness, sadness, surprise	Identification (score); Discrimination (score)	6 (GOOD)	Yes
Benito et al., 2013 [53], Spain	Cross-sectional	Outpatients	BD (44) HCs (48)	Euthymic (100%)	42.3 ± 11 45.73 ± 12.2	64% 63%	DSM-IV-TR	POFA, FOE, ER–40	Anger, disgust, fear, happiness, sadness, shame, surprise	Identification (score); Discrimination (score)	6 (FAIR)	Yes
Bio et al., 2013 [54], Brazil	Cross-sectional	Outpatients	BD (39) HCs (40)	Euthymic (100%)	32.9 ± 10.9 25.9 ± 5.8	62% 50%	DSM-IV-TR (SCID-P)	POFA, EK	Anger, disgust, fear, happiness, sadness, surprise	Identification (accuracy)	5 (FAIR)	No
Bjertrup et al., 2021 [55], Denmark	Prospective cohort	Outpatients	BD (7) HCs (29)	Euthymic (100%)	29.4 ± 4.2 29.2 ± 6.5	100% 100%	DSM-IV (MINI)	POFA	Anger, disgust, fear, happiness, sadness, surprise	Identification (accuracy)	8 (GOOD)	Yes
Bora et al., 2005 [56], Turkey	Cross-sectional	NA	BD (43) HCs (30)	Euthymic (100%)	38.6 ± 9.33 38.93 ± 10.23	47% 57%	DSM-IV (SCID)	Faces test, Benton facial recognition test	Anger, disgust, distress, fear, happiness, sadness, surprise	Identification (score)	7 (GOOD)	Yes
Bozikas et al., 2006 [57], Greece	Cross-sectional	Outpatients	BD (19) HCs (30)	Euthymic (100%)	39 ± 11 38 ± 10	58% 50%	DSM-IV (MINI)	KAMT	Anger, disgust, fear, happiness, sadness, surprise	Discrimination (score)	7 (GOOD)	Yes
Bozorg et al., 2014 [58], Iran	Cross-sectional	Outpatients	BD (30) HCs (30)	Euthymic (100%)	15.43 ± 1.27 15.07 ± 1.57	55% 55%	DSM-IV (K-SADS)	Cohn-Kanade	Anger, happiness, sadness	Identification (accuracy, reaction time)	8 (GOOD)	Yes
Branco et al., 2018 [59], Brazil	Cross-sectional	Outpatients	BD (30) HCs (45)	Euthymic (33%) Depressed (67%)	42.9 ± 13.12 26.16 ± 10.13	80% 47%	DSM–5 (MINI)	NA	Anger, disgust, fear, happiness, sadness, surprise	Identification (score)	7 (GOOD)	Yes
Brotman et al., 2008 [60], USA	Cross-sectional	NA	BD (52) HCs (78) FDRs (24)	Euthymic (65%) Depressed (6%) Manic/mixed (29%)	13.3 ± 2.85 14.43 ± 2.28 11.45 ± 3.98	50% 53% 29%	DSM-IV-TR (SCID)	DANVA	Anger, fear, happiness, sadness	Identification (number of errors)	6 (GOOD)	Yes
Brotman et al., 2008 [61], USA	Cross-sectional	NA	BD (37) HCs (36) FDRs (25)	Euthymic (46%) Depressed (14%) Manic/mixed (40%)	14.16 ± 2.92 14.34 ± 2.28 12.15 ± 3.05	64% 44% 28%	DSM-IV-TR (SCID)	POFA, EEMT	Anger, disgust, fear, happiness, sadness, surprise	Identification (number of morphs before response, number of morphs before correct response)	6 (GOOD)	No
Bürger et al., 2017 [62], Germany	Cross-sectional	Inpatients	BD (36) HCs (36)	Depressed (100%)	38.56 ± 12.3 41.33 ± 6.05	50% 53%	DSM-IV (SCID)	NimStim database	Anger, fear, happiness	Discrimination (accuracy, reaction time)	7 (GOOD)	Yes
Darke et al., 2021 [63], Australia	Cross-sectional	Inpatients	BD (15) HCs (20)	The sample is not euthymic	36.6 ± 14.8 34.05 ± 10.72	40% 40%	DSM-IV	MIMI, FEED	Disgust, fear	Identification (d prime); Discrimination (d prime)	4 (POOR)	Yes
Daros et al., 2014 [64], Canada	Prospective cohort	NA	BD (16) HCs (32)	Depressed (31%) Manic (50%) Mixed (19%)	23.63 ± 6.27 25.78 ± 6.81	44% 66%	DSM-IV (SCID)	PEAT	Happiness, sadness	Identification (z-score)	6 (FAIR)	Yes
David et al., 2014 [65], Brazil	Cross-sectional	NA	BD (110) HCs (96)	Euthymic (35%) Depressed (37%) Manic/mixed (28%)	29.86 ± 7.21 24.3 ± 4.7	68% 53%	DSM-IV-TR (SCID)	POFA	Anger, disgust, fear, happiness, sadness, surprise	Identification (accuracy)	6 (GOOD)	No
de Siqueira Rotenberg et al., 2023 [66], Denmark	Prospective cohort	Outpatients	BD (275) HCs (190)	NA	31.8 ± 9.2 31.4 ± 11.2	65% 62%	ICD–10 (SCAN)	P1vital Affective Faces	Anger, disgust, fear, happiness, sadness, surprise	Identification (d prime)	8 (GOOD)	Yes
Derntl et al., 2009 [67], Germany	Cross-sectional	Outpatients	BD (62) HCs (62)	NA	42.87 ± 9.6 42.89 ± 10.19	44% 60%	DSM-IV (MINI)	VERT-K	Anger, disgust, fear, happiness, sadness	Identification (accuracy)	7 (GOOD)	Yes
Derntl et al., 2012 [68], Germany	Cross-sectional	Inpatients and outpatients	BD (24) HCs (24)	NA	44 ± 9.8 39.9 ± 10	50% 50%	DSM-IV (MINI)	3D Facial expression task	Anger, disgust, fear, happiness, sadness	Identification (score)	7 (GOOD)	Yes
Dima et al., 2016 [69], UK	Cross-sectional	Outpatients	BD (41) HCs (46) FDRs (25)	Euthymic (100%)	44.3 ± 11.9 40.3 ± 13.2 39.7 ± 13.7	51% 46% 48%	DSM-IV	POFA	Anger, fear, sadness	Identification (accuracy, reaction time)	6 (GOOD)	Yes
Fernandes et al., 2016 [70], Brazil	Cross-sectional	Outpatients	BD (23) HCs (27) FDRs (22)	Euthymic (100%)	37 ± 9 33 ± 10 32 ± 7	30% 37% 36%	DSM-IV (SCID)	ER–40	Anger, fear, happiness, sadness	Identification (score, false positive score, reaction time); Discrimination (score, reaction time)	7 (GOOD)	Yes
Foland-Ross et al., 2012 [71], USA	Cross-sectional	Outpatients	BD (24) HCs (26)	Euthymic (100%)	33.8 ± 12.8 37.9 ± 13.4	38% 42%	DSM-IV (SCID)	NA	NA	Identification (accuracy, reaction time); Discrimination (accuracy, reaaction time)	7 (GOOD)	Yes
Fulford et al., 2014 [72], USA	Cross-sectional	Outpatients	BD (60) HCs (43)	Euthymic (100%)	37.1 ± 11.8 33.5 ± 12.2	65% 58%	DSM-IV (SCID)	FEEST	Anger, fear, happiness, sadness	Identification (accuracy)	6 (FAIR)	No
Furlong et al., 2022 [73], USA	Cross-sectional	Outpatients	BD (34) HCs (32)	Euthymic (71%)	38 ± 12.08 34.94 ± 11.12	36% 56%	DSM-IV (SCID)	POFA	Anger, fear, sadness	Identification (accuracy, reaction time)	7 (GOOD)	No
Getz et al., 2003 [74], USA	Cross-sectional	Inpatients	BD (25) HCs (25)	Manic/mixed (100%)	25.3 ± .4 25.3 ± 7.4	52% 64%	DSM-IV (SCID)	POFA	Anger, disgust, fear, happiness, sadness, surprise	Identification (score); Discrimination (score)	7 (GOOD)	Yes
Goghari et al., 2013 [75], USA	Cross-sectional	Outpatients	BD (16) HCs (30)	NA	46.2 ± 11.3 48.8 ± 9.7	19% 48%	DSM-IV (DIGS)	Pennsylvania emotive faces	Anger, fear, happiness, sadness	Identification (accuracy, reaction time)	7 (GOOD)	Yes
Golkhatmi et al., 2015 [76], Iran	Cross-sectional	NA	BD (30) HCs (30)	Manic/mixed (100%)	29.13 ± 8.08 33.63 ± 11.71	53% 47%	DSM-IV	POFA	Anger, disgust, fear, happiness, sadness, surprise	Identification (score)	4 (POOR)	Yes
Gray et al., 2024 [77], New Zealand	Cross-sectional	NA	BD (108) HCs (61)	NA	27.3 ± 7 37.7 ± 12.7	75% 67%	DSM-IV (SCID)	POFA	Anger, disgust, fear, happiness, sadness, surprise	Identification (accuracy)	4 (POOR)	Yes
Guyer et al., 2007 [78], USA	Cross-sectional	Outpatients	BD (42) HCs (92)	NA	12.8 ± 2.5 14.4 ± 2.4	48% 49%	DSM-IV (K-SADS-PL)	DANVA	Anger, fear, happiness, sadness	Identification (number of errors)	7 (GOOD)	Yes
Harmer et al., 2002 [79], UK	Cross-sectional	Outpatients	BD (20) HCs (20)	Euthymic (100%)	37.8 ± 2.5 37.7 ± 3.8	50% 35%	DSM-IV (SCID)	POFA	Anger, disgust, fear, happiness, sadness, surprise	Identification (accuracy, reaction time)	7 (GOOD)	Yes
Hassel et al., 2008 [80], USA	Cross-sectional	Outpatients	BD (15) HCs (21)	Euthymic (100%)	32.47 ± 8.8 27.78 ± 8.7	47% 57%	DSM-IV (SCID)	KDEF	Anger, disgust, fear, happiness, sadness	Identification (accuracy)	5 (FAIR)	No
Hoertnagl et al., 2011 [81], Austria	Cross-sectional	Outpatients	BD (47) HCs (45)	Euthymic (100%)	42.2 ± 10.2 39.9 ± 6.2	62% 47%	DSM-IV (MINI)	JACFEE, FEEL	Anger, disgust, fear, happiness, sadness, surprise	Identification (accuracy)	7 (GOOD)	Yes
Hulvershorn et al., 2012 [82], USA	Cross-sectional	Outpatients	BD (75) HCs (30)	Euthymic (20%) Depressed (40%) Manic/mixed (40%)	33.8 ± 11 32 ± 10	63% 63%	DSM-IV-TR (MINI)	NimStim database, 3-minute emotion-matching task	Anger, fear	Discrimination (accuracy, reaction time, reaction time for correct responses)	8 (GOOD)	Yes
Hwang et al., 2021 [83], South Korea	Cross-sectional	Outpatients	BD (53) HCs (50)	NA	27 ± 6.8 25.4 ± 5.8	51% 52%	DSM–5	Emotion perception test	Pleasant, unpleasant	Discrimination (accuracy, reaction time)	7 (GOOD)	Yes
Iakimova et al., 2016 [84], France	Cross-sectional	Outpatients	BD (34) HCs (29)	Euthymic (100%)	50.38 ± 11.46 43.7 ± 17.5	67% 47%	DSM-IV-TR	Radboud faces database	Anger, disgust, fear, happiness, sadness, surprise	Identification (accuracy)	6 (FAIR)	Yes
Jogia et al., 2011 [85], Denmark	Cross-sectional	Outpatients	BD (41) HCs (50) FDRs (25)	Euthymic (100%)	44.29 ± 11.87 34.96 ± 13.2 35 ± 13.67	51% 46% 48%	DSM-IV-TR (SCID)	POFA	Fear	Identification (accuracy, reaction time)	7 (GOOD)	Yes
Kaufmann et al., 2017 [86], Norway	Cross-sectional	NA	BD (97) HCs (136)	Euthymic (46%) Depressed (31%) Manic/mixed (23%)	33.69 ± 10.93 35.21 ± 8.66	59% 41%	DSM-IV (SCID)	NA	Positive, negative	Discrimination	6 (GOOD)	No
Khafif et al., 2023 [87], Brazil	Cross-sectional	Outpatients	BD (36) HCs (22)	Euthymic (36%) Depressed (3%) Manic (48%) Mixed (13%)	12–17 12–17	67% 27%	DSM-IV (K-SADS-PL)	ER–40	Anger, fear, happiness, sadness	Identification (score, reaction time)	5 (POOR)	Yes
Kim et al., 2013 [88], USA	Cross-sectional	Outpatients	BD (22) HCs (22)	Euthymic (73%) Depressed (4%) Manic (14%) Mixed (9%)	15.42 ± 1.97 14.14 ± 2.26	31% 45%	DSM-IV-TR (K-SADS-PL)	POFA	Anger, fear, happiness, sadness	Identification (accuracy)	6 (GOOD)	Yes
Kjærstad et al., 2022 [89], Denmark	Prospective cohort	Outpatients	BD (266) HCs (190) FDRs (76)	NA	31.2 ± 9 31.4 ± 11.6 29 ± 9.4	65% 62% 53%	ICD–10 (SCAN)	P1vital Affective Faces	Anger, disgust, fear, happiness, sadness, surprise	Identification (accuracy, reaction time, d prime)	8 (GOOD)	Yes
Lahera et al., 2015 [90], Spain	Cross-sectional	Outpatients	BD (46) HCs (50)	Euthymic (100%)	38.6 ± 10.63 43.4 ± 13.6	63% 58%	DSM-IV-TR	FEIT, FEDT, ER–40	Anger, disgust, fear, happiness, sadness, shame, surprise	Identification (score); Discrimination (score)	6 (FAIR)	Yes
Lawlor-Savage et al., 2014 [91], USA	Cross-sectional	Outpatients	BD (17) HCs (50)	Euthymic (100%)	46.53 ± 11.03 45.9 ± 11.51	18% 50%	DSM-IV	3D Facial expression task	Anger, fear, happiness, sadness	Identification (accuracy)	6 (FAIR)	Yes
Lee et al., 2020 [92], USA	Cross-sectional	Outpatients	BD (29) HCs (29)	NA	34.76 ± 12.9 35.41 ± 13.3	83% 69%	DSM-IV (MINI)	ER–96	Anger, fear, happiness, sadness	Identfication (accuracy)	5 (FAIR)	Yes
Lelli-Chiesa et al., 2011 [93], UK	Cross-sectional	Outpatients	BD (40) HCs (50) FDRs (25)	Euthymic (100%)	44 ± 11.9 34.9 ± 13.2 34.9 ± 14.3	53% 48% 60%	DSM-IV (SCID)	POFA	Sadness	Identification (score, reaction time)	6 (FAIR)	Yes
Lembke et al., 2002 [94], USA	Cross-sectional	Inpatients and outpatients	BD (24) HCs (10)	Euthymic (66%) Manic/mixed (34%)	NA	NA	DSM-IV (SCID)	POFA	Anger, disgust, fear, happiness, sadness, surprise	Identification	4 (POOR)	No
Lescalier et al., 2015 [95], France	Cross-sectional	Outpatients	BD (28) HCs (28)	Euthymic (100%)	36.93 ± 9.68 38.64 ± 12.65	82% 64%	DSM-IV-TR (SCID-P)	KDEF	Happiness, sadness	Identification (hu index)	7 (GOOD)	Yes
Li et al., 2022 [96], China	Cross-sectional	Outpatients	BD (29) HCs (29)	Euthymic (100%)	31.34 ± 8.6 27.93 ± 6.44	70% 48%	DSM-IV (SCID)	CAFPS	Anger, fear	Discrimination (accuracy, reaction time)	7 (GOOD)	Yes
MacPherson et al., 2021 [97], USA	Prospective-cohort	Outpatients	BD (52) HCs (64)	NA	20.89 ± 2.7 21.75 ± 2.19	40% 44%	DSM-IV (SCID, K-SADS)	DANVA–2	Anger, fear, happiness, sadness	Identification (number of errors)	6 (FAIR)	Yes
Maila de Castro et al., 2015 [98], Brazil	Cross-sectional	Outpatients	BD (21) HCs (21)	Euthymic (100)	39.95 ± 13.54 37.86 ± 8.22	52% 53%	DSM-IV-TR (MINI-plus)	ER–40	Anger, fear, happiness, sadness	Identification (score, reaction time)	7 (GOOD)	Yes
Malhi et al., 2007 [99], USA	Cross-sectional	Outpatients	BD (10) HCs (10)	Euthymic (100%)	33.5 ± 8.7 33.6 ± 6.4	100% 100%	DSM-IV (SCID-P)	POFA	Disgust, fear	Identification (percentage of errors, reaction time)	7 (GOOD)	Yes
Martino et al., 2011 [100], Argentina	Cross-sectional	Outpatients	BD (81) HCs (34)	Euthymic (100%)	39.7 ± 10.3 39.7 ± 12.5	65% 65%	DSM-IV (SCID)	POFA, EK	Anger, disgust, fear, happiness, sadness, surprise	Identification (score)	8 (GOOD)	Yes
McClure et al., 2005 [101], USA	Cross-sectional	Outpatients	BD (38) HCs (20)	Euthymic (71%)	12.9 ± 2.7 13.5 ± 2	45% 36%	DSM-IV (K-SADS-PL)	DANVA	Anger, fear, happiness, sadness	Identification (number of errors)	7 (GOOD)	Yes
Millett et al., 2023 [102], USA	Cross-sectional	Outpatients	BD (202) HCs (53)	Euthymic (74%)	43.4 ± 2.2 37.9 ± 12.2	47% 57%	DSM-IV (SCID)	CANTAB	Anger, disgust, fear, happiness, sadness, surprise	Identification (accuracy)	6 (GOOD)	Yes
Miola et al., 2023 [103], Italy	Cross-sectional	Outpatients	BD (48) HCs (39)	Euthymic (100%)	35.81 ± 12.6 40.1 ± 12.8	33% 41%	DSM–5 (SCID)	NimStim database	Anger, disgust, sadness	Identification (accuracy, reaction time)	7 (GOOD)	Yes
Mourão-Miranda et al., 2012 [104], UK	Cross-sectional	NA	BD (18) HCs (18)	Depressed (100%)	36 ± 11 30 ± 9	78% 89%	DSM-IV-TR (SCID-P)	FEEST	Happiness	Identification (accuracy)	6 (FAIR)	No
Navarra-Ventura et al., 2021 [105], Spain	Cross-sectional	Outpatients	BD (60) HCs (40)	Euthymic (100%)	47.2 ± 8.75 45.8 ± 10.5	50% 50%	DSM-IV-TR	POFA	Anger, disgust, fear, happiness, sadness, surprise	Identification (score)	7 (GOOD)	Yes
Nigam et al., 2021 [106], India	Cross-sectional	Outpatients	BD (27) HCs (28) FDRs (20)	Euthymic (100%)	44.03 ± 11.01 37 ± 9.51 42.7 ± 12.76	37% 39% 60%	ICD–10	TRENDS	Anger, disgust, fear, happiness, sadness, surprise	Identification (score)	6 (FAIR)	Yes
Pan et al., 2013 [107], Taiwan	Cross-sectional	Inpatients	BD (45) HCs (40)	Euthymic (41%) Manic/mixed (59%)	35.46 ± 11.61 33.75 ± 10.64	53% 63%	DSM-IV (DIGS)	DANVA–2	Anger, fear, happiness, sadness	Identification (accuracy)	7 (GOOD)	Yes
Pavuluri et al., 2009 [108], USA	Cross-sectional	Outpatients	BD (10) HCs (10)	Euthymic (100%)	15.2 ± 2 14.3 ± 2.1	50% 50%	DSM-IV (WASH-U-KSADS)	Gur Faces	Anger, happiness	Identification (accuracy, reaction time)	6 (FAIR)	Yes
Priyesh et al., 2022 [109], India	Cross-sectional	NA	BD (26) HCs (26)	Manic (100%)	36.6 ± 9.5 35.7 ± 10.5	50% 50%	DSM–5	TRENDS	Anger, happiness	Identification (accuracy, reaction time)	6 (GOOD)	Yes
Quidé et al., 2018 [110], Australia	Cross-sectional	NA	BD (84) HCs (75)	NA	37.47 ± 12.14 36.13 ± 10.52	63% 45%	ICD–10 (DIP)	TASIT, POFA	Anger, disgust, fear, happiness, sadness, surprise	Identification (score)	6 (POOR)	Yes
Reddy et al., 2022 [111], India	Cross-sectional	Outpatients	BD (30) FDRs (21) HCs (30)	Euthymic (100%)	30.63 ± 8.33 27.19 ± 4.55 28.87 ± 6.02	23% 40% 36%	DSM-IV (MINI)	TRENDS	Anger, disgust, fear, sadness,	Discrimination (accuracy, reaction time)	7 (GOOD)	Yes
Robinson et al., 2015 [112], UK (Study 1)	Cross-sectional	Outpatients	BD (38) HCs (28)	Euthymic (100%)	44.8 ± 12.8 46.5 ± 10.8	55.3% 53.6%	DSM-IV (SCID)	POFA	Anger, disgust, fear, happiness, sadness	Identification (accuracy)	7 (GOOD)	Yes
Robinson et al., 2015 [112], UK (Study 2)	Cross-sectional	Outpatients	BD (53) HCs (47)	Depressed (100%)	47.3 ± 9.6 45 ± 13.7	37.7% 53.6%	DSM-IV (SCID)	POFA	Anger, disgust, fear, happiness, sadness	Identification (accuracy)	7 (GOOD)	Yes
Rossell et al., 2014 [113], Australia	Cross-sectional	Outpatients	BD (43) HCs (112)	Euthymic (25%) Depressed (75%)	40.5 ± 10.64 35.14 ± 12.4	63% 67%	DSM-IV (SCID)	Comprehensive Affective Testing System	Anger, disgust, fear, happiness, sadness, surprise	Identification (accuracy, reaction time)	5 (POOR)	Yes
Rowland et al., 2013 [114], Australia	Cross-sectional	NA	BD (33) HCs (58)	Euthymic (36.4%) Depressed (3%) Mixed (24.2%) Hypomanic (36.4%)	40.67 ± 11.27 33.91 ± 12.24	45.7% 50%	DSM-IV	POFA, TASIT	Anger, disgust, fear, happiness, sadness, surprise	Identification (accuracy, score)	4 (POOR)	Yes
Rubin et al., 2022 [115], USA	Cross-sectional	NA	BD (113) HCs (236)	NA	38.1 ± 11.6 34.4 ± 12.6	55% 58%	DSM-IV (SCID)	Cohn-Kanade face database	Anger, disgust, fear, happiness, sadness, surprise	Identification (accuracy, reaction time)	6 (POOR)	Yes
Ruihua et al., 2021 [116], China	Cross-sectional	Inpatients and outpatients	BD (30) HCs (30)	Depressed (100%)	24.25 ± 9.03 28.45 ± 7.59	56.7% 26.7%	DSM-IV	POFA	Anger, disgust, fear, happiness, sadness, surprise	Identification (reaction time)	5 (FAIR)	No
Ruocco et al., 2014 [117], USA	Cross-sectional	NA	BD (248) FDRs (286) HCs (380)	NA	36.22 ± 12.72 40.25 ± 15.98 37.71 ± 12.69	63% 53% 64%	DSM-IV (SCID)	ER–40	Anger, fear, happiness, sadness	Identification (accuracy, reaction time)	6 (POOR)	Yes
Ryan et al., 2013 [118], USA	Cross-sectional	Inpatients and outpatients	BD (156) HCs (143)	Euthymic (100%)	37.39 ± 11.75 32.2 ± 13.12	42% 44%	DSM-IV (DIGS)	POFA	Anger, disgust, fear, happiness, sadness, surprise	Identification (accuracy, reaction time)	6 (GOOD)	Yes
Sagar et al., 2013 [119], USA	Cross-sectional	NA	BD (23) HCs (18)	Euthymic (100%)	26.65 ± 6.65 23.11 ± 3.15	NA NA	DSM-IV (SCID)	FEEST	Anger, disgust, fear, happiness, sadness, surprise	Identification (accuracy)	5 (POOR)	Yes
Schaefer et al., 2010 [120], USA	Cross-sectional	NA	BD (21) HCs (24)	Depressed (100%)	46.8 ± 11.8 45.3 ± 3.9	62% 50%	DSM-IV (SCID)	POFA	Anger, disgust, fear, happiness, sadness, surprise	Identification (accuracy)	6 (GOOD)	Yes
Schenkel et al., 2007 [121], USA	Cross-sectional	NA	BD (58) HCs (28)	NA	11.94 ± 3.19 11.19 ± .57	41% 54%	DSM-IV (K-SADS-PL)	Pennsylvania emotive faces	Anger, happiness	Identification (accuracy) Discrimination (accuracy)	6 (FAIR)	No
Schenkel et al., 2012 [122], USA	Cross-sectional	NA	BD (39) HCs (31)	Depressed (20%) (Hypo)manic (60%) Mixed (20%)	13.58 ± 2.76 13 ± 3.4	51% 45%	DSM-IV-TR (K-SADS-PL)	DANVA	Fear, happiness	Identification (accuracy)	7 (GOOD)	Yes
Schenkel et al., 2020 [123], USA	Cross-sectional	Outpatients	BD (25) HCs (25)	NA	12 ± 3.2 12.44 ± 2.84	28% 36%	DSM-IV (K-SADS-PL)	DANVA–2	Anger, fear, happiness, sadness	Identification (accuracy)	7 (GOOD)	Yes
Seidel et al., 2012 [124], Germany	Cross-sectional	Outpatients	BD (21) HCs (21)	NA	46 ± 11.49 41.67 ± 9.13	42% 47.6%	DSM-IV (MINI)	Gur Faces	Anger, disgust, fear, happiness, sadness	Identification (accuracy, reaction time)	6 (GOOD)	Yes
Seymour et al., 2013 [125], USA	Cross-sectional	NA	BD (30) HCs (41)	Euthymic (70%) Depressed (10%) Manic (13%) Mixed (7%)	13.3 ± 2.99 12.24 ± 3.23	33% NA	DSM-IV (K-SADS-PL)	DANVA	Anger, fear, happiness, sadness	Identification (number of errors)	6 (GOOD)	Yes
Shah et al., 2009 [126], USA	Cross-sectional	NA	BD (30) HCs (48)	Euthymic (100%)	32.75 ± 13.95 27.6 ± 9.8	67% 58%	DSM-IV (SCID)	POFA	Fear, happiness	Identification (accuracy, reaction time)	6 (GOOD)	Yes
Sinha et al., 2020 [127], India	Cross-sectional	NA	BD (30) HCs (30)	Manic (100%)	28.07 ± 7.16 30.93 ± 5.96	33% 33%	ICD–10 (SCAN)	NA	Anger, fear, happiness, sadness	Identification	7 (GOOD)	No
Soeiro-de-Souza et al., 2012 [128], Brasil	Cross-sectional	NA	BD (64) HCs (75)	Depressed (64%) Manic (36%)	28.16 ± 5.24 23.54 ± 3.53	NA NA	DSM-IV-TR (SCID)	POFA	Anger, disgust, fear, happiness, sadness, surprise	Identification (score)	7 (GOOD)	Yes
Soeiro-de-Souza et al., 2012 [129], Brasil	Cross-sectional	Outpatients	BD (39) HCs (40)	Euthymic (100%)	32.9 ± 10.9 25.9 ± 5.8	62% 50%	DSM-IV-TR (SCID)	POFA	Anger, disgust, fear, happiness, sadness, surprise	Identification (score)	6 (GOOD)	Yes
Summers et al., 2006 [130], UK	Cross-sectional	Outpatients	BD (36) HCs (30)	NA	39 NA	64% NA	DSM-IV (SCID)	POFA	Anger, disgust, fear, happiness, sadness, surprise	Identification (accuracy)	6 (GOOD)	Yes
Tesli et al., 2015 [131], Norway	Cross-sectional	NA	BD (80) HCs (119)	Euthymic (31%) Depressed (40%)	34.8 ± 11.2 35 ± 8.8	61.2% 43.8%	DSM-IV (SCID)	POFA	Anger, fear, happiness	Identification (accuracy, reaction time)	6 (POOR)	Yes
Thaler et al., 2013 [132], USA	Cross-sectional	Outpatients	BD (48) HCs (24)	Euthymic (100%)	35.85 ± 13.2 36.1 ± 13.4	38% 54%	DSM-IV (SCID)	BLERT	Anger, disgust, fear, happiness, sadness, surprise	Identification (number of errors)	6 (GOOD)	Yes
Ulusoy et al., 2020 [133], Turkey	Cross-sectional	Outpatients	BD (38) HCs (30) FDRs (60)	Euthymic (55%) Depressed (29%) Manic/mixed (16%)	28.42 ± 5.65 28.83 ± 5.7 53.91 ± 6.08	55% 53% 50%	DSM-IV (SCID)	FEIT, FEDT	Anger, fear, happiness, sadness, shame, surprise	Identification (score); Discrimination (score)	8 (GOOD)	Yes
Van Rheenen et al., 2014 [134], Australia	Cross-sectional	Outpatients	BD (50) HCs (52)	Euthymic (34%) Depressed (34%) Manic (8%) Mixed (24%)	37.92 ± 12.45 33.98 ± 14.27	68% 62%	DSM-IV-TR (MINI)	POFA	Anger, fear, happiness, sadness	Identification (accuracy, reaction time)	7 (GOOD)	Yes
Van Rheenen et al., 2017 [135], Australia	Cross-sectional	NA	BD (28) HCs (28)	Euthymic (57%) Depressed (43%)	41.96 ± 11.35 42.43 ± 11.29	69% 53.4%	DSM-IV	POFA	Anger, fear, happiness, sadness	Identification (accuracy, reaction time)	4 (POOR)	Yes
Vaskinn et al., 2007 [136], Norway	Cross-sectional	NA	BD (21) HCs (31)	NA	38.1 ± 9.3 30.7 ± 9.6	48% 35%	DSM-IV (SCID)	POFA, FOE	Anger, disgust, happiness, sadness, shame, surprise	Identification (score); Discrimination (score)	6 (GOOD)	Yes
Vederman et al., 2012 [137], USA	Cross-sectional	Inpatients and outpatients	BD (119) HCs (66)	NA	37 ± 11.8 37.2 ± 13.2	67% 64.2%	DSM-IV (SCID, DIGS)	NA	Anger, fear, happiness, sadness	Identification (accuracy)	7 (GOOD)	Yes
Venn et al., 2004 [138], UK	Cross-sectional	Outpatients	BD (17) HCs (17)	Euthymic (100%)	44.35 ± 3.2 43.76 ± 3.36	41% 41%	DSM-IV (SCID)	POFA	Anger, disgust, fear, happiness, sadness, surprise	Identification (score)	7 (GOOD)	Yes
Versace et al., 2010 [139], USA	Cross-sectional	NA	BD (31) HCs (24)	Euthymic (54.8%) Depressed (45.2%)	35.9 ± 8.8 29.5 ± 5.2	65% 54%	DSM-IV (SCID)	POFA	Happiness, sadness	Identification (score)	6 (POOR)	Yes
Vierck et al., 2015 [36], New Zealand	Cross-sectional	Outpatients	BD (36) FDRs (24) HCs (40)	Euthymic (53%) Depressed (44%) Mixed (3%)	40.8 ± 11.6 33.2 ± 13.5 36.2 ± 11.3	75% 70.8% 72.5%	DSM-IV (SCID)	POFA	Anger, disgust, fear, happiness, sadness	Identification (accuracy, reaction time)	6 (GOOD)	Yes
Wegbreit et al., 2015 [140], USA	Cross-sectional	Outpatients	BD (66) HCs (87)	Euthymic (77.3%)	16.7 ± 4.7 17.9 ± 4.7	36.4% 39.1%	DSM-IV (K-SADS-PL)	DANVA–2	Anger, fear, happiness, sadness	Identification (number of errors)	5 (FAIR)	No
Wiggins et al., 2017 [141], USA	Cross-sectional	NA	BD (36) FDRs (22) HCs (41)	Euthymic (81%) Depressed (5%) Hypomanic (14%)	17.9 ± 3.3 15.7 ± 3.6 17.31 ± 4.2	42% 41% 49%	DSM–5 (K-SADS-PL, SCID)	POFA	Anger, fear, happiness	Identification (accuracy)	7 (GOOD)	Yes
Wynn et al., 2013 [142], USA	Cross-sectional	Outpatients	BD (57) HCs (30)	NA	44.9 ± 10.4 40.6 ± 10.1	43% 36.7%	DSM-IV (SCID)	POFA	Anger, fear, happiness, sadness, shame, surprise	Identification (accuracy)	7 (GOOD)	Yes
Yalcin-Siedentopf et al., 2014 [143], Austria	Cross-sectional	Outpatients	BD (57) HCs (50)	Euthymic (100%)	41.9 ± 11.7 38.8 ± 6.9	65% 42%	DSM-IV (MINI)	FEEL	Anger, disgust, fear, happiness, sadness, surprise	Identification (score)	6 (FAIR)	Yes
Zhang et al., 2018 [144], China	Cross-sectional	NA	BD (61) HCs (54)	NA	20.03 ± 1.9 20.68 ± 2.83	55.7% 54%	DSM–5	NA	Anger, happiness, sadness	Identification (accuracy, reaction time)	5 (FAIR)	Yes

**Abbreviations:** BD, bipolar disorder; BLERT, Bell-Lysaker emotion recognition test; CAFPS, Chinese affective facial picture system; CANTAB, Cambridge Neuropsychological Test Automated Battery; Cohn-Kanade, Cohn-Kanade action unit-coded facial expression database; DANVA, diagnostic analysis of non-verbal accuracy; DIGS, Diagnostic Interview for Genetic Studies; DIP, The Diagnostic Interview for Psychoses; DSM-III-R, Diagnostic and Statistical Manual of Mental Disorders – third ed. Revised; DSM-IV, Diagnostic and Statistical Manual of Mental Disorders – fourth ed.; DSM-IV-TR, Diagnostic and Statistical Manual of Mental Disorders – fourth ed. – Text Revision; DSM-5, Diagnostic and Statistical Manual of Mental Disorders – fifth ed.; EEMT, Emotional Expression Multimorph Task; ER-40, Penn Emotion Recognition-40; ER-96, Penn Emotion Recognition-96; EK, Ekman 60 Faces Test; FDRs, First-Degree Relatives; FEDT, Facial Emotion Discrimination Test; FEIT, Facial Emotion Identification Test; FEED, Facial Expression and Emotions Database; FEEL, Facially Expressed Emotion Labeling; FEEST, Facial Expressions of Emotion: Stimuli and Tests; FOE, The Face of Emotions; HCs, healthy controls; ICD-10, International Classification of Diseases; JACFEE, Japanese and Caucasian Facial Expressions of Emotion; KAMT, Kinney’s Affect Matching Test; KDEF, Karolinska Directed Emotional Faces; K-SADS-PL, Kiddie Schedule for Affective Disorders and Schizophrenia, present and lifetime version; MIMI, MIMI Facial Expression Database; MINI, The Mini-International Neuropsychiatric Interview; Mini-SEA, mini-Social Cognition and Emotional Assessment; NA, not available; NOS, Newcastle-Ottawa Scale; PEAT, Penn Emotion Acuity Test; POFA, Pictures of Facial Affect; SCAN, Schedules for Clinical Assessment in Neuropsychiatry; SCID, Structured Clinical Interview for DSM Disorders; SCID-P, Structured Clinical Interview for DSM Disorders-Patient version; TRENDS, Tool for Recognition of Emotions in Neuropsychiatric Disorders; VERT-K, Vienna Emotion Recognition Tasks; WASH-U-KSADS, Washington University Schedule for Affective Disorders and Schizophrenia.

### Characteristics of included studies

The 100 studies included were published between 1996 and 2024 in 21 countries worldwide. Ninety-four studies (94%) were cross-sectional, and six (6%) were longitudinal. Regarding the age group, 84 (84%) were conducted in adults, 12 (12%) in children/adolescents, and four (4%) in mixed age groups.

Individuals with BD (*n* = 4920, range = 7–275; females = 56%, mean age = 34.1 ± 9.1) had an age at onset of 21.2 ± 7.6 years and a duration of illness of 13.6 ± 8.7 years. 74.5% were diagnosed with BD-I. Regarding their mood state, 62.9% were euthymic, 18.1% depressed, and 12.5% (hypo)manic. In 21 studies (21%), the information regarding the mood state was unclear. Control groups included both FDRs (*n* = 676, range = 20–286, females = 55%, mean age = 36.1 ± 12), and HCs (*n* = 4909, range = 10–380, females = 53.2%, mean age = 32.5 ± 9.5).

Ninety-four studies (94%) used a FER identification task, and 19 studies (19%) used a FER discrimination task (Supplementary Appendix IV).

### Risk of bias evaluation

Sixty-five studies (65%) were considered “good quality” according to the AHRQ standards, 20 studies (20%) were considered “fair quality,” and 15 studies (15%) were considered “poor quality.” Additional details are available in Table 1 and in Supplementary Appendix V.

### Main analyses

**Figure 2.**
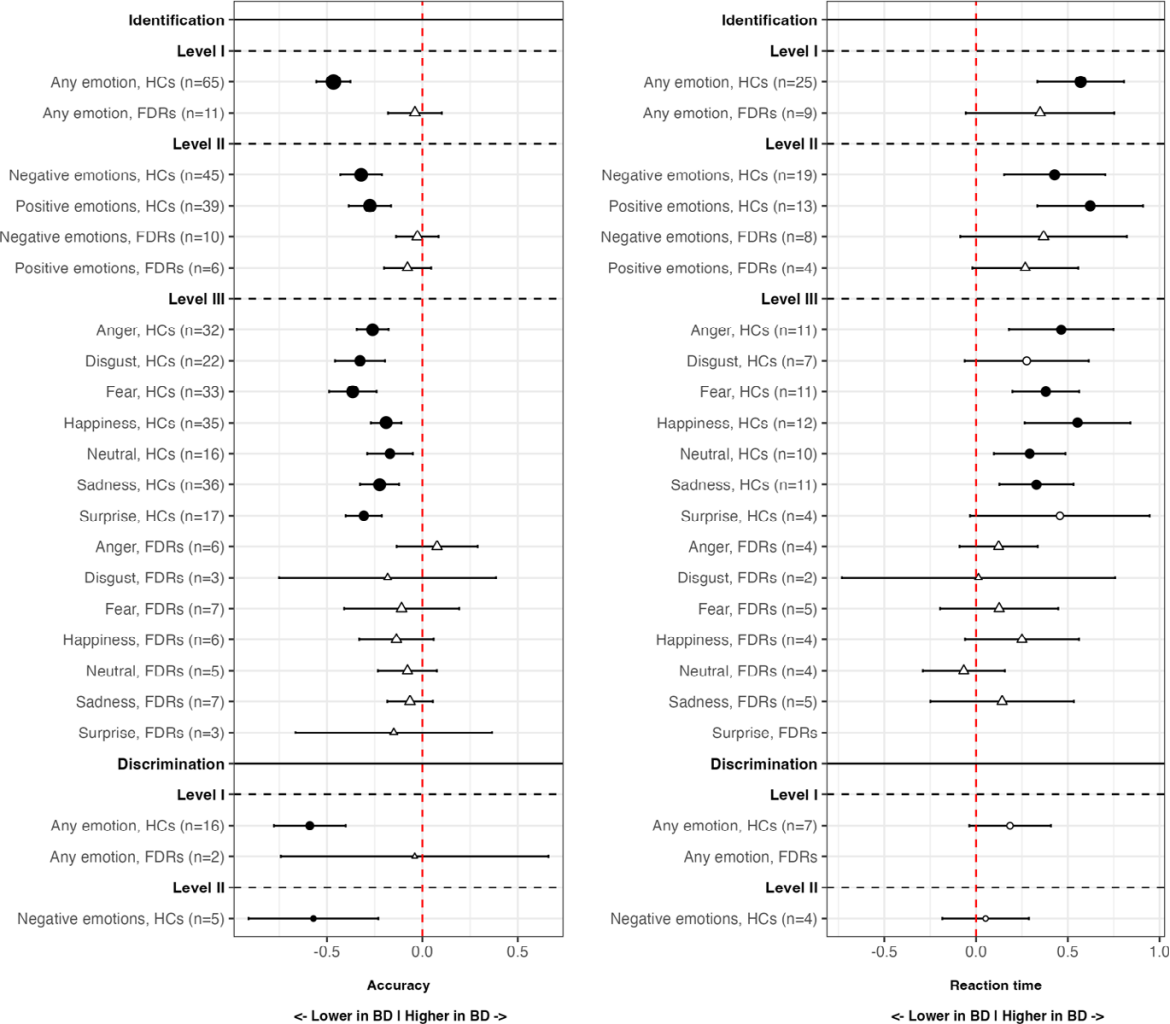
Jungle plot for differences in facial emotion recognition accuracy (left) and reaction time (right), adults only. BD, bipolar disorder; FDRs, first-degree relatives; HCs, healthy controls. Circles represent HCs, while triangles represent FDRs. Black-filled circles or triangles indicate statistically significant comparisons, while white-filled ones indicate non-significant comparisons. Point size is proportional to the number of patients included in that specific comparison.

Table 2 displays the main results of all the meta-analyses conducted. Figure 2 is the jungle plot [23] for the differences in FER accuracy and reaction time among adult populations.

**Table 2.** Results of the meta-analyses in detail

Control group	Emotion type	Outcome type	Studies, *n*	BD patients, *n*	Controls, *n*	SMD	95% CIs	*p*-value	95% PIs	*I* ^2^	tau^2^	*Q* test *p*-value
***Identification***												
*Adults*												
Level I												
HCs	Any emotion	Accuracy	65	3221	3334	**−0.466**	**−0.556, −0.377**	**<0.001**	−1.015, 0.082	63.44	0.08	<0.1
HCs	Any emotion	Number of errors	3	110	98	0.253	−0.026, 0.532	0.08	−0.026, 0.532	0	0	0.85
HCs	Any emotion	Reaction time	25	1453	1796	**0.57**	**0.334, 0.806**	**<0.001**	−0.521, 1.661	89.27	0.3	<0.1
FDRs	Any emotion	Accuracy	11	811	605	−0.039	−0.18, 0.102	0.58	−0.299, 0.22	23.48	0.01	0.39
FDRs	Any emotion	Reaction time	9	746	525	0.349	−0.056, 0.753	0.09	−0.832, 1.529	88.91	0.32	<0.1
Level II												
HCs	Negative	Accuracy	45	2364	2435	**−0.321**	**−0.43, −0.212**	**<0.001**	−0.894, 0.251	66.29	0.08	<0.1
HCs	Negative	Number of errors	3	110	98	0.177	−0.102, 0.456	0.21	−0.102, 0.456	0	0	0.94
HCs	Negative	Reaction time	19	1160	1445	**0.428**	**0.153, 0.704**	**0.002**	−0.701, 1.558	90.1	0.31	<0.1
HCs	Positive	Accuracy	39	2155	2185	**−0.275**	**−0.386, −0.164**	**<0.001**	−0.804, 0.254	63.22	0.07	<0.1
HCs	Positive	Reaction time	13	950	1220	**0.622**	**0.334, 0.91**	**<0.001**	−0.345, 1.588	88.17	0.22	<0.1
FDRs	Negative	Accuracy	10	790	584	−0.026	−0.138, 0.085	0.64	−0.138, 0.085	0	0	0.65
FDRs	Negative	Reaction time	8	725	504	0.368	−0.086, 0.822	0.11	−0.908, 1.644	90.84	0.37	<0.1
FDRs	Positive	Accuracy	6	638	488	−0.078	−0.201, 0.046	0.22	−0.201, 0.046	0	0	0.45
FDRs	Positive	Reaction time	4	573	408	0.268	−0.02, 0.557	0.07	−0.266, 0.803	67.36	0.05	<0.1
Level III												
HCs	Anger	Accuracy	32	1907	1903	**−0.261**	**−0.344, −0.177**	**<0.001**	**−0.504, −0.018**	26.34	0.01	0.15
HCs	Anger	Number of errors	2	100	88	0.263	−0.032, 0.557	0.08	−0.032, 0.557	0	0	0.73
HCs	Anger	Reaction time	11	0862	1066	**0.464**	**0.179, 0.749**	**0.001**	−0.404, 1.332	86.14	0.17	<0.1
HCs	Disgust	Accuracy	22	1269	1153	**−0.327**	**−0.458, −0.196**	**<0.001**	−0.764, 0.111	51.95	0.05	<0.1
HCs	Disgust	Number of errors	2	58	34	0.343	−0.088, 0.774	0.12	−0.088, 0.774	0	0	0.56
HCs	Disgust	Reaction time	7	508	565	0.276	−0.062, 0.614	0.11	−0.545, 1.097	80.83	0.15	<0.1
HCs	Fear	Accuracy	33	1892	1896	**−0.365**	**−0.489, −0.24**	**<0.001**	−0.92, 0.191	65.71	0.08	<0.1
HCs	Fear	Number of errors	3	110	98	0.068	−0.211, 0.347	0.63	−0.211, 0.347	0	0	0.56
HCs	Fear	Reaction time	11	818	1061	**0.38**	**0.198, 0.562**	**<0.001**	−0.079, 0.839	61.3	0.05	<0.1
HCs	Happiness	Accuracy	35	2044	2042	**−0.19**	**−0.27, −0.11**	**<0.001**	−0.43, 0.049	25.75	0.01	<0.1
HCs	Happiness	Number of errors	3	152	152	0.064	−0.164, 0.293	0.58	−0.164, 0.293	0	0	0.98
HCs	Happiness	Reaction time	12	924	1194	**0.553**	**0.265, 0.841**	**<0.001**	−0.376, 1.481	87.99	0.2	<0.1
HCs	Neutral	Accuracy	16	950	1001	**−0.17**	**−0.289, −0.05**	**0.005**	−0.424, 0.085	25.4	0.01	0.25
HCs	Neutral	Number of errors	2	58	34	0.028	−0.4, 0.456	0.9	−0.4, 0.456	0	0	0.49
HCs	Neutral	Reaction time	10	758	838	**0.292**	**0.097, 0.487**	**0.003**	−0.185, 0.769	60.94	0.05	<0.1
HCs	Sadness	Accuracy	36	2036	2020	**−0.224**	**−0.327, −0.122**	**<0.001**	−0.658, 0.209	53.64	0.05	<0.1
HCs	Sadness	Number of errors	2	100	88	0.174	−0.12, 0.468	0.25	−0.12, 0.468	0	0	0.69
HCs	Sadness	Reaction time	11	886	1096	**0.329**	**0.127, 0.531**	**0.001**	−0.235, 0.894	72.9	0.07	<0.1
HCs	Surprise	Accuracy	17	912	937	**−0.307**	**−0.402, −0.213**	**<0.001**	**−0.402, −0.213**	0	0	0.32
HCs	Surprise	Reaction time	4	414	476	0.457	−0.033, 0.946	0.07	−0.544, 1.457	88.45	0.2	<0.1
FDRs	Anger	Accuracy	6	638	488	0.077	−0.135, 0.29	0.48	−0.334, 0.489	51.32	0.03	<0.1
FDRs	Anger	Reaction time	4	573	408	0.123	−0.09, 0.336	0.26	−0.224, 0.47	43.57	0.02	0.19
FDRs	Disgust	Accuracy	3	329	120	−0.182	−0.751, 0.386	0.53	−1.221, 0.857	79.25	0.2	<0.1
FDRs	Disgust	Reaction time	2	302	100	0.013	−0.731, 0.758	0.97	−1.213, 1.24	84.88	0.25	<0.1
FDRs	Fear	Accuracy	7	679	513	−0.109	−0.41, 0.193	0.48	−0.84, 0.623	77.79	0.12	<0.1
FDRs	Fear	Reaction time	5	614	433	0.126	−0.196, 0.448	0.44	−0.552, 0.804	76.18	0.09	<0.1
FDRs	Happiness	Accuracy	6	638	488	−0.136	−0.331, 0.059	0.17	−0.492, 0.22	42.98	0.02	0.14
FDRs	Happiness	Reaction time	4	573	408	0.25	−0.06, 0.561	0.11	−0.338, 0.838	71.77	0.06	<0.1
FDRs	Neutral	Accuracy	5	600	428	−0.078	−0.233, 0.076	0.32	−0.284, 0.128	14.07	0	0.53
FDRs	Neutral	Reaction time	4	573	408	−0.067	−0.29, 0.157	0.56	−0.44, 0.307	47.93	0.02	0.13
FDRs	Sadness	Accuracy	7	678	513	−0.065	−0.184, 0.055	0.29	−0.184, 0.055	0	0	0.83
FDRs	Sadness	Reaction time	5	613	433	0.142	−0.249, 0.533	0.48	−0.719, 1.003	84.03	0.15	<0.1
FDRs	Surprise	Accuracy	3	331	156	−0.15	−0.665, 0.365	0.57	−1.089, 0.789	79.51	0.16	<0.1
*Children/adolescents*												
Level I												
HCs	Any emotion	Accuracy	6	162	140	**−0.588**	**−0.821, −0.355**	**<0.001**	**−0.821, −0.355**	0	0	0.44
HCs	Any emotion	Number of errors	4	162	231	**0.659**	**0.447, 0.87**	**<0.001**	0.447, 0.87	0	0	0.89
HCs	Any emotion	Reaction time	3	76	62	**0.352**	**0.01, 0.694**	**0.044**	0.01, 0.694	0	0	0.36
Level II												
HCs	Negative	Accuracy	4	130	108	**−0.541**	**−0.802, −0.279**	**<0.001**	**−0.802, −0.279**	0	0	0.73
HCs	Negative	Number of errors	3	110	153	**0.493**	**0.235, 0.751**	**<0.001**	**0.235, 0.751**	0	0	0.7
HCs	Negative	Reaction time	2	66	52	2.468	−1.965, 6.901	0.28	−5.169, 10.105	98.4	10.07	<0.1
HCs	Positive	Accuracy	4	130	108	**−0.581**	**−0.924, −0.238**	**<0.001**	**−1.137, −0.025**	40.86	0.05	0.17
HCs	Positive	Number of errors	3	110	153	0.281	−0.371, 0.933	0.4	−0.936, 1.498	83.22	0.27	<0.1
HCs	Positive	Reaction time	2	66	52	**0.418**	**0.048, 0.788**	**0.027**	**0.048, 0.788**	0	0	0.73
Level III												
HCs	Anger	Accuracy	4	130	108	**−0.429**	**−0.772, −0.086**	**0.014**	−0.99, 0.133	41.99	0.05	0.16
HCs	Anger	Number of errors	3	110	153	**0.447**	**0.189, 0.705**	**<0.001**	**0.189, 0.705**	0	0	0.29
HCs	Anger	Reaction time	2	66	52	0.209	−0.356, 0.774	0.47	−0.619, 1.037	57.29	0.1	0.13
HCs	Fear	Accuracy	2	61	47	−0.326	−0.711, 0.06	0.1	−0.711, 0.06	0	0	0.8
HCs	Fear	Number of errors	3	110	153	**0.364**	**0.107, 0.621**	**0.005**	**0.107, 0.621**	0	0	0.7
HCs	Happiness	Accuracy	4	130	108	**−0.581**	**−0.924, −0.238**	**<0.001**	**−1.137, −0.025**	40.86	0.05	0.17
HCs	Happiness	Number of errors	3	110	153	**0.643**	**0.382, 0.904**	**<0.001**	**0.382, 0.904**	0	0	0.94
HCs	Happiness	Reaction time	2	66	52	**0.418**	**0.048, 0.788**	**0.027**	**0.048, 0.788**	0	0	0.73
HCs	Neutral	Accuracy	2	66	52	−0.285	−0.653, 0.082	0.13	−0.653, 0.082	0	0	0.88
HCs	Neutral	Reaction time	2	66	52	0.463	−0.084, 1.009	0.1	−0.324, 1.249	53.51	0.08	0.14
HCs	Sadness	Accuracy	3	91	77	**−0.588**	**−0.901, −0.276**	**<0.001**	**−0.901, −0.276**	0	0	0.75
HCs	Sadness	Number of errors	3	110	153	**0.703**	**0.442, 0.965**	**<0.001**	**0.442, 0.965**	0	0	0.82
HCs	Sadness	Reaction time	2	66	52	4.905	−3.556, 13.367	0.26	−9.694, 19.505	98.85	36.85	<0.1
*Mixed age groups*												
Level I												
HCs	Any emotion	Accuracy	3	160	134	−0.22	−0.565, 0.124	0.21	−0.751, 0.31	45.28	0.04	0.16
Level II												
HCs	Negative	Accuracy	3	160	134	−0.127	−0.443, 0.189	0.43	−0.587, 0.332	36.28	0.03	0.19
HCs	Positive	Accuracy	3	160	134	**−0.365**	**−0.727, −0.002**	**0.049**	−0.937, 0.208	49.69	0.05	0.14
Level III												
HCs	Anger	Accuracy	2	144	102	−0.058	−0.315, 0.199	0.66	−0.315, 0.199	0	0	0.53
HCs	Fear	Accuracy	2	144	102	−0.09	−0.347, 0.167	0.49	−0.347, 0.167	0	0	0.83
HCs	Happiness	Accuracy	3	160	134	**−0.425**	**−0.664, −0.186**	**<0.001**	**−0.664, −0.186**	0	0	0.5
HCs	Neutral	Accuracy	2	124	93	−0.261	−0.833, 0.31	0.37	−1.139, 0.616	65.2	0.12	<0.1
HCs	Sadness	Accuracy	2	124	93	−0.076	−0.965, 0.812	0.87	−1.541, 1.388	85.13	0.35	<0.1
***Discrimination***												
*Adults*												
Level I												
HCs	Any emotion	Accuracy	16	549	606	**−0.59**	**−0.778, −0.402**	**<0.001**	**−1.175, −0.004**	56.23	0.08	<0.1
HCs	Any emotion	Reaction time	7	290	313	0.185	−0.037, 0.408	0.1	−0.253, 0.624	41.6	0.04	0.12
FDRs	Any emotion	Accuracy	2	68	81	−0.04	−0.742, 0.661	0.91	−1.153, 1.073	75.52	0.19	<0.1
Level II												
HCs	Negative	Accuracy	5	185	145	**−0.571**	**−0.911, −0.231**	**<0.001**	**−1.216, 0.074**	53.1	0.08	<0.1
HCs	Negative	Reaction time	4	170	125	0.052	−0.184, 0.288	0.66	−0.184, 0.288	0	0	0.92

Abbreviations: BD, bipolar disorder; CIs, confidence intervals; FDRs, first-degree relatives; HCs, healthy controls; PIs, prediction intervals; SMD, standardized mean difference. Statistically significant results (p < 0.05) are highlighted in bold.

Regarding the identification of any emotion, adults with BD were less accurate (*k* = 65; SMD = −0.47; 95% CIs = −0.56, −0.38; *p*-value <0.001; *I* ^2^= 63.4%) and showed higher reaction time (*k* = 25; SMD = 0.57; 95%CIs = 0.33, 0.81; *p*-value <0.001; *I* ^2^ = 89.3%) compared to HCs.

Regarding the identification of negative and positive emotions, adults with BD were less accurate (*k* = 45; SMD = −0.32; 95%CIs = −0.43, −0.21; *p*-value <0.001; *I* ^2^ = 66.3%), and showed higher reaction time (*k* = 19; SMD = 0.43; 95%CIs = 0.15, 0.7; *p*-value = 0.002; *I* ^2^ = 90.1%) compared to HCs, during the recognition of negative emotions. Similarly, they were less accurate (*k* = 39; SMD = −0.28; 95%CIs = −0.39, −0.16; *p*-value <0.001; *I* ^2^ = 63.2%), and showed higher reaction time (*k* = 13; SMD = 0.62; 95%CIs = 0.33, 0.91; *p*-value <0.001; *I* ^2^ = 88.2%), during the recognition of positive emotions too.

Regarding the identification of specific types of emotions, adults with BD were less accurate than HCs in recognizing anger (*k* = 32; SMD = −0.26; 95%CIs = −0.34, −0.18; *p*-value <0.001; *I* ^2^ = 26.3%), disgust (*k* = 22; SMD = −0.33; 95%CIs = −0.46, −0.20; *p*-value <0.001; *I* ^2^ = 52%), fear (*k* = 33; SMD = −0.37; 95%CIs = −0.49, −0.24; *p*-value <0.001; *I* ^2^ = 65.7%), happiness (*k* = 35; SMD = −0.19; 95%CIs = −0.27, −0.11; *p*-value <0.001; *I* ^2^ = 25.8%), neutral (*k* = 16; SMD = −0.17; 95%CIs = −0.29, −0.05; *p*-value = 0.005; *I* ^2^ = 25.4%), sadness (*k* = 36; SMD = −0.22; 95%CIs = −0.33, −0.12; *p*-value <0.001; *I* ^2^ = 53.6%), and surprise (*k* = 17; SMD = −0.31; 95%CIs = −0.4, −0.21; *p*-value <0.001; *I* ^2^ = 0%), and showed higher reaction time during the recognition of anger (*k* = 11; SMD = 0.46; 95%CIs = 0.18, 0.75; *p*-value = 0.001; *I* ^2^ = 86.1%), fear (*k* = 11; SMD = 0.38; 95%CIs = 0.2, 0.56; *p*-value <0.001; *I* ^2^ = 61.3%), happiness (*k* = 12; SMD = 0.55; 95%CIs = 0.27, 0.84; *p*-value <0.001; *I* ^2^ = 88%), neutral (*k* = 10; SMD = 0.29; 95%CIs = 0.1, 0.49; *p*-value = 0.03; *I* ^2^ = 61%), and sadness (*k* = 11; SMD = 0.33; 95%CIs = 0.13, 0.53; *p*-value = 0.001; *I* ^2^ = 72.9%).

Additional details are presented in Supplementary Appendix VI.

### Meta-regression analyses

Characteristics of the original study, sociodemographic and clinical characteristics of people with BD, and FER task characteristics were chosen as predictors in the meta-regression analyses. Table 3 displays the significant predictors among all the meta-regressions conducted.

**Table 3.** Results of the meta-regression analyses, only significant predictors reported

Predictor	Studies No	Beta (95% CIs)	Association	From smaller to larger SMD	From larger to smaller SMD	SMDs opposite in directions
***Identification***						
*Characteristics of the original study*						
Publication year (from lower to higher)	25	−0.053 (−0.102, −0.003)	BD versus HCs, any emotion, reaction time		1.02 to 0.18	
	19	−0.059 (−0.111, −0.008)	BD versus HCs, negative emotions, reaction time		0.97 to 0.02	
	11	−0.046 (−0.068, −0.025)	BD versus HCs, fear, reaction time		0.79 to 0.1	
	11	−0.064 (−0.088, −0.04)	BD versus HCs, sadness, reaction time			0.77 to −0.06
*Sociodemographic characteristics of people with BD*						
Age (from younger to older)	36	0.017 (0.002, 0.033)	BD versus HCs, sadness, accuracy		−0.53 to −0.01	
	25	0.051 (0.016, 0.086)	BD versus HCs, any emotion, reaction time			−0.35 to 0.99
	13	0.048 (0.01, 0.085)	BD versus HCs, positive emotions, reaction time			−0.14 to 1.11
	12	0.043 (0.004, 0.081)	BD versus HCs, happiness, reaction time			−0.13 to 0.99
Education (from less to more years)	21	0.075 (0.006, 0.144)	BD versus HCs, happiness, accuracy			−0.41 to 0.15
	21	0.082 (0.008, 0.157)	BD versus HCs, sadness, accuracy			−0.5 to 0.11
	13	0.138 (0.016, 0.26)	BD versus HCs, disgust, accuracy			−0.57 to 0.19
Percentage of females (from lower to higher)	21	−1.161 (−2.098, −0.223)	BD versus HCs, disgust, accuracy	−0.05 to −0.83		
*Clinical characteristics of people with BD*						
Age at onset (from younger to older)	10	0.137 (0.096, 0.177)	BD versus HCs, any emotion, reaction time			−0.1 to 3
Percentage of people with BD-I (from lower to higher)	24	−0.391 (−0.707, −0.075)	BD versus HCs, happiness, accuracy			0.06 to −0.33
Symptoms severity scale, mania (from lower to higher score)	42	−0.024 (−0.046, −0.003)	BD versus HCs, any emotion, accuracy	−0.35 to −1.02		
	14	−0.148 (−0.278, −0.017)	BD versus HCs, negative emotions, reaction time			0.83 to −0.47
*FER task characteristics*						
Duration of stimulus (from shorter to longer)	43	−0.00004 (−6e–05, −1e–05)	BD versus HCs, any emotion, accuracy	−0.32 to −0.9		
	10	0.00014 (2e–05, 0.00025)	BD versus HCs, positive emotions, reaction time	0.37 to 1.42		
Practice (from absence to presence of practice before the task)	65	0.254 (0.033, 0.476)	BD versus HCs, any emotion, accuracy		−0.51 to −0.26	
	19	−0.596 (−1.163, −0.028)	BD versus HCs, negative emotions, reaction time			0.58 to −0.01
Number of stimuli (from lower to higher)	12	−0.003 (−0.006, −0.0001)	BD versus HCs, positive emotions, reaction time		0.94 to 0.16	
***Discrimination***						
*Sociodemographic characteristics of people with BD*						
Education (from less to more years)	11	0.181 (0.035, 0.328)	BD versus HCs, any emotion, accuracy		−1.09 to −0.26	
*Clinical characteristics of people with BD*						
Percentage of people with depression (from lower to higher)	12	0.667 (0.206, 1.127)	BD versus HCs, any emotion, accuracy		−0.76 to −0.09	

Abbreviations: BD, bipolar disorder; CIs, confidence intervals; HCs, healthy controls; SMD, standardized mean difference.

Among the characteristics of the original study, higher publication years were associated to smaller differences between adults with BD and HCs regarding the reaction time during the identification of any emotion (*k* = 25; beta = −0.05; 95%CIs = −0.1, −0.003), the reaction time during the identification of negative emotions (*k* = 19; beta = −0.06; 95%CIs = −0.11, −0.01), and the reaction time during the identification of fear (*k* = 11; beta = −0.05; 95%CIs = −0.07, −0.03).

Among the sociodemographic characteristics of people with BD, older age was associated to smaller differences between adults with BD and HCs regarding the accurate identification of sadness (*k* = 36; beta = 0.02; 95%CIs = 0.002, 0.03), and higher percentage of females was associated to bigger differences between adults with BD and HCs regarding the accurate identification of disgust (*k* = 21; beta = −1.16; 95%CIs = −2.1, −0.22). More years of education were associated to smaller differences between adults with BD and HCs regarding the accurate discrimination of any emotion (*k* = 11; beta = 0.18; 95%CIs = 0.04, 0.33).

Among the clinical characteristics of people with BD, more severe manic symptoms were associated to bigger differences between adults with BD and HCs regarding the accurate identification of any emotion (*k* = 42; beta = −0.02; 95%CIs = −0.05, −0.003). Higher percentage of people experiencing a depressive episode were associated to smaller differences between adults with BD and HCs regarding the accurate discrimination of any emotion (*k* = 12; beta = 0.67; 95%CIs = 0.21, 1.13).

Among the FER task characteristics, longer duration of stimuli was associated to bigger differences between adults with BD and HCs regarding the accurate identification of any emotion (*k* = 43; beta = −4e−05; 95%CIs = −6e−05, −1e−05), and the reaction time during the identification of positive emotions (*k* = 10; beta = 1.4e−04; 95%CIs = 2e−05, 2.5e−04). On the other hand, presence of practice was associated to smaller differences between adults with BD and HCs regarding the accurate identification of any emotion (*k* = 65; beta = 0.25; 95%CIs = 0.03, 0.48), and the higher number of stimuli was associated to smaller differences between adults with BD and HCs regarding the reaction time during the identification of positive emotions (*k* = 12; beta = −0.003; 95%CIs = −0.006, −1e04).

Additional details are presented in Supplementary Appendix VI.

### Sensitivity analyses

Sensitivity analyses were conducted: (a) by excluding one study at a time from the main analysis; (b) by including only good-quality studies according to AHRQ standards; (c) by removing those studies whose lower-level data were calculated from higher-level information.

Additional details are presented in Supplementary Appendix VI.

### Publication bias

Publication bias was examined in 21 comparisons with at least 10 available studies. Among these comparisons, publication bias was identified in 10 of them.

Additional details are presented in Supplementary Appendix VI.

## Discussion

This systematic review and meta-analysis aimed to investigate the differences in FER among BD, FDRs, and HCs. Overall, adults with BD were less accurate than HCs on all FER identification and discrimination tasks, and they required more time to respond on all FER identification tasks, except those related to disgust and surprise. Differences were more pronounced with greater stimulus durations and manic symptoms severity, and less pronounced when participants had practice before the task. These deficits seem to manifest early in life, as even children/adolescents presented reduced accuracy and increased reaction times compared to HCs. No significant differences were found comparing BD and FDRs.

Regarding comparisons with HCs, our results are consistent with findings observed in other domains of AC, such as emotion regulation [9], and affective decision-making and reward processing [8]. The observed deficits in FER may be explained by several reasons. From a brain functioning perspective, patients with BD undergoing emotion processing tasks like FER appear to exhibit consistent hyperactivation in amygdala activity compared to HCs [24]. The amygdala is a hub for different networks involved in core affect representation, playing a significant role in emotional stimulus generation and perception [25]. Its hyperactivation may indicate heightened sensitivity to emotional stimuli, potentially contributing to worsened mood symptoms or the genesis of mood episodes [24]. Consistently, alterations in other dimensions of AC associated with changes in amygdala activity, such as emotion dysregulation, were also found to be associated with more severe mood symptoms [26], with dimensions more strongly influenced by amygdala hyperactivity showing a stronger correlation to symptoms [27]. Extended neurocognitive impairments observed in BD may also contribute to difficulties in FER. As AC involves the integration of emotions and neurocognitive processes, challenges in FER may depend on the integrity of neurocognitive performance. A post-hoc analysis [28] showed that BD patients with a cognitive performance similar to HCs had also similar FER skills, while impaired neurocognitive functions were associated to deficits in FER. This observation was confirmed by another study [29] and maintained when the problem was analyzed the other way around. Indeed, emotionally preserved BD patients generally exhibited better cognitive abilities, especially in attention, psychomotor speed, working memory, and executive functions [30].

For many years, cognitive symptoms in BD have been overshadowed by an almost exclusive focus on depressive and manic symptomatology, leaving them underappreciated despite their significant impact on patients’ lives. Nowadays, cognitive impairments are increasingly recognized as a core feature of BD, persisting throughout the illness, even in the absence of acute mood episodes [31]. This growing awareness has led to the development of interventions targeting cognitive functions, yet most treatments focus on neurocognitive domains without specifically addressing AC [32–35]. Indeed, evidence on interventions aimed at improving FER in BD remains limited. While selective serotonin reuptake inhibitors have shown efficacy in enhancing FER in some individuals [36], results are inconsistent [37]. Similarly, antipsychotics, which are effective in reducing manic symptoms, may indirectly affect FER by impairing eye-gaze movements [38], which complicate task performance. Our findings, confirming FER impairments in BD, underscore the importance of future research focused on developing treatments specifically targeting domains of AC.

We observed high heterogeneity in nearly half of our results, which can be partly attributed to the neurocognitive variability inherent to BD, as described above. In addition, FER was not uniformly assessed across each study. Different paradigms were used, including the use of facial expressions from multiple atlases, consideration of a varying range of emotions or stimulus durations, or allowing for a trial before task execution. Only 20% of the included studies used facial expressions morphed from neutral or mild intensity to full intensity expressions. This is critical because the International Society of Bipolar Disorder Targeting Cognition Task Force has recommended the use of FER tasks using morphed faces as the gold standard for studying this domain in BD [6]. While we believe that such aspects should be controlled upstream, encouraging future studies to use standardized tasks aligned with existing guidelines, we attempted to control this heterogeneity with numerous meta-regressions to identify some important variables in predicting our outcomes.

Among FER task characteristics, we observed that an increased stimulus duration was associated with an increase in the magnitude of differences in correctly identifying facial expressions (SMD from −0.32 to −0.9) or in the speed with which participants provided a response in identifying positive emotions (SMD from 0.37 to 1.42). A brief stimulus duration could pose challenges for both BD patients and HCs in correctly performing a FER task. However, the difficulties experienced by BD patients may be more evident with longer stimulus durations, where issues related to attention or information integration become more apparent. Conversely, studies that did not include a trial before the actual task showed greater differences in identifying facial expressions compared to those that allowed for a preliminary practice (SMD from −0.51 to −0.26). This suggests that the difficulties experienced by BD patients may be partially alleviated through training. Regarding the clinical characteristics of individuals with BD, the severity of manic symptoms was associated with greater difficulty in identifying facial expressions (SMD from −0.35 to −1.02). Patients with more severe manic symptoms exhibit greater distractibility and impulsivity [39], which may explain this finding.

Considering comparisons with FDRs, we did not find significant differences. Although not manifesting the pathology, FDRs share genetic heritage with their family members and thus genetic risk factors. Therefore, our results support the hypothesis that difficulties in AC may constitute an endophenotype of BD [6], as observed in previous reports about AC domains such as emotion regulation [9]. However, studies comparing these two populations are very scarce. Additionally, FDRs of individuals diagnosed with other psychiatric conditions, such as schizophrenia, have also shown FER deficits when compared to HCs [40], raising the question of whether these deficits could be transdiagnostic endophenotypes rather than illness-specific ones [41]. Hence, further research is necessary to draw more robust conclusions.

To the best of our knowledge, this is the first and most comprehensive systematic review and meta-analysis focusing on FER in individuals diagnosed with BD compared to FDRs and HCs. By synthesizing data from numerous studies conducted across different countries and age groups while controlling for several predictors, our analysis provides comprehensive insights into the challenges faced by individuals with BD in perceiving and interpreting facial expressions. From a clinical standpoint, these findings support a shift toward personalized, multidomain treatment strategies in BD. FER performance should be systematically assessed as part of comprehensive cognitive profiling at baseline and follow-up to identify individuals who may benefit from integrated interventions. The presence of FER deficits in both patients and unaffected FDRs suggests they may represent a cognitive endophenotype of BD, offering a potential biomarker for early identification and risk stratification in clinical settings. The observed association between manic symptom severity and FER impairments further suggests that difficulties in emotion recognition intensify during periods of mood instability, underscoring the importance of monitoring social cognitive functioning when designing individualized treatment plans. By integrating FER assessment into routine cognitive profiling, clinicians can implement more precise treatment sequencing, where mood stabilization serves also as a foundation for subsequent cognitive interventions aimed at improving interpersonal functioning and quality of life. Similarly, our findings offer valuable directions for future research, suggesting specific areas requiring exploration in individual studies. For example, our observation of practice’s positive impact supports the development of targeted interventions aimed at improving cognitive functions. This highlights the potential for tailored interventions considering also the specific deficits identified in our meta-analysis. While various interventions targeting specific domains of AC have been proposed in different populations [42–44], we believe in a therapeutic approach aimed at enhancing AC as a cohesive whole rather than focusing solely on discrete aspects such as emotion regulation, impulsivity, or reward processing. Additionally, given that these aspects are common even in young people and individuals at risk, we believe that AC should also be integrated into early intervention programs to address vital aspects necessary for individuals’ growth within their social environment.

The present work has some limitations. First, affective state of participants with BD was not always reported, limiting our speculation on the role of affective symptomatology in FER. However, we addressed this aspect through meta-regressions related to both the severity of affective symptomatology and the percentage of individuals in a specific mood state, finding a relationship between the severity of manic symptomatology and the accurate identification of facial emotions. Second, we could not control for the type of pharmacological therapy taken due to the heterogeneity of treatments and the limited availability of information. Medication could serve as an important confounder that limits the generalizability of our results [45], although the majority of our sample was euthymic and likely on stable therapy. Third, individuals with BD often have comorbidities with other psychiatric disorders [46], which may influence differences in FER. However, most studies employed (semi)structured diagnostic interviews, enhancing the reliability of reported information. Fourth, although we did not find significant differences between individuals with BD and FDRs, it would be important to compare FDRs with HCs to further test the endophenotype hypothesis. While this comparison was not among the objectives of our study, future research should consider it to provide a better understanding. Fifth, we observed publication bias in some comparisons. Nonetheless, this review searched four databases and used a search strategy without time, language, or age restrictions, maximizing the likelihood of capturing all relevant published studies.

Our analysis provide evidence that people with BD exhibit extensive impairments in FER when compared to HCs, across all emotional categories. These differences are not observed when comparing individuals with BD to their FDRs, suggesting that FER impairments could be considered as an endophenotype of BD. Our findings can guide the development of new treatments for individuals with BD that target AC to enhance cognitive functions, promote social functioning, and improve the overall quality of life.

## Supporting information

10.1192/j.eurpsy.2025.10147.sm001 De Prisco et al. supplementary material De Prisco et al. supplementary material

## Supplementary material

The supplementary material for this article can be found at http://doi.org/10.1192/j.eurpsy.2025.10147.

## Open practices statement

The dataset used for this research is available on request.
