# Supplementary material for: Facial emotion recognition deficits in bipolar disorder: A systematic review and meta-analysis
Source: Eur Psychiatry. 2026 Jan 12;69(1):e16. doi: 10.1192/j.eurpsy.2025.10147 (PMC12925675; doi:10.1192/j.eurpsy.2025.10147)
Supplement: De Prisco et al. supplementary material [file S0924933825101478sup001.docx]

**Facial emotion recognition deficits in bipolar disorder: a systematic review and meta-analysis**

Michele De Prisco ^1,2,3,4^, Vincenzo Oliva ^1,2,3,4^, Chiara Possidente ^5^, Giovanna Fico ^1,2,3^, Laura Montejo ^1,2,3,4^, Lydia Fortea ^1,3,6^, Hanne Lie Kjærstad ^7^, Kamilla Woznica Miskowiak ^7,8^, Gerard Anmella ^1,2,3,4^, Diego Hidalgo-Mazzei ^1,2,3,4^, Alessandro Miola ^9,10^, Michele Fornaro ^11^, Andrea Murru ^1,2,3^, Eduard Vieta ^1,2,3,4*^, Joaquim Radua ^1,3,4,6^

^1^ Department of Medicine, Faculty of Medicine and Health Sciences, Institute of Neurosciences (UBNeuro), University of Barcelona (UB), C. Casanova, 143, 08036 Barcelona, Catalonia, Spain.

^2^ Bipolar and Depressive Disorders Unit, Hospìtal Clinic de Barcelona. c. Villarroel, 170, 08036 Barcelona, Catalonia, Spain.

^3^ Institut d’Investigacions Biomèdiques August Pi i Sunyer (IDIBAPS), c. Villarroel, 170, 08036 Barcelona, Catalonia, Spain.

^4^ Centro de Investigación Biomédica en Red de Salud Mental (CIBERSAM), Instituto de Salud Carlos III, Madrid, Spain

^5^ Department of Biomedical and Neuromotor Sciences, University of Bologna, Bologna, Italy

^6^ Imaging of Mood- and Anxiety-Related Disorders (IMARD) Group, Barcelona, Catalonia, Spain

^7^ Neurocognition and Emotion in Affective Disorder Centre (NEAD), Psychiatric Centre Copenhagen, Frederiksberg Hospital, Denmark

^8^ Department of Psychology, University of Copenhagen, Denmark

^9^ Department of Neuroscience, University of Padova, Padua, Italy;

^10^ Department Psychiatry and Psychology, Mayo Clinic, Rochester, MN, USA;

^11^ Section of Psychiatry, Department of Neuroscience, Reproductive Science and Odontostomatology Federico II University of Naples, Naples, Italy

* Corresponding Author

Prof. Eduard Vieta, PhD

Bipolar and Depressive Disorders Unit,

Institute of Neuroscience

IDIBAPS CIBERSAM

Hospital Clinic, University of Barcelona,

170 Villarroel St 12-0, 08036 Barcelona, Catalonia, Spain.

Email: [evieta@clinic.cat](mailto:evieta@clinic.cat)

[APPENDIX I 3](#_Toc211446900)

[Abbreviations used throughout the document 3](#_Toc211446901)

[Deviations from the protocol 4](#_Toc211446902)

[MOOSE Checklist 5](#_Toc211446903)

[APPENDIX II 7](#_Toc211446904)

[Search strategy 7](#_Toc211446905)

[Graphical representation of the three-level scheme used 9](#_Toc211446906)

[APPENDIX III 10](#_Toc211446907)

[Excluded studies, with reasons 10](#_Toc211446908)

[APPENDIX IV 25](#_Toc211446909)

[Studies included only at a systematic review level 25](#_Toc211446910)

[Characteristics of the tasks adopted by the included studies 28](#_Toc211446911)

[APPENDIX V 34](#_Toc211446912)

[Quality according to the Newcastle-Ottawa Scale of included cross-sectional studies 34](#_Toc211446913)

[Quality according to the Newcastle-Ottawa Scale of included longitudinal studies 37](#_Toc211446914)

[APPENDIX VI 38](#_Toc211446915)

[Main analysis 38](#_Toc211446916)

[Meta-regression analyses 39](#_Toc211446917)

[Sensitivity analyses, leave-one-out sensitivity analysis 47](#_Toc211446918)

[Sensitivity analyses, only good quality studies 64](#_Toc211446919)

[Sensitivity analyses, removing those studies whose lower-level data were calculated from higher-level information 67](#_Toc211446920)

[Publication bias 68](#_Toc211446921)

# APPENDIX I

## Abbreviations used throughout the document

***AHRQ***, Agency for Healthcare Research and Quality; ***BD***, Bipolar Disorder; ***BLERT***, Bell-Lysaker Emotion Recognition Test; ***CAFPS***, Chinese affective facial picture system; ***CIs***, Confidence Intervals; ***CANTAB***, Cambridge Neuropsychological Test Automated Battery; ***Cohn-Kanade***, Cohn-Kanade Action Unit-Coded Facial Expression Database; ***DANVA***, Diagnostic Analysis of Non-Verbal Accuracy; ***DARE***, Dynamic Affect Recognition Evaluation Task; ***DIGS*** - Diagnostic Interview for Genetic Studies; ***DIP*** - The Diagnostic Interview for Psychoses; ***DSM*** - Diagnostic and Statistical Manual of Mental Disorders; ***EEMT***, Emotional Expression Multimorph Task; ***EK***, Ekman 60 Faces Test; ***ER-40***, Penn Emotion Recognition-40; ***ER-96***, Penn Emotion Recognition-96; ***FDRs***, First-degree Relatives; ***FEDT***, Facial Emotion Discrimination Test; ***FEED***, Facial Expression and Emotions Database; ***FEIT***, Facial Emotion Identification Test; ***FEEL***, Facial Expression Emotion Labeling; ***FEEST***, Facial Expressions of Emotion: Stimuli and Tests; ***FEPT***, Facial Emotion Perception Test; ***FER***, Facial Emotion Recognition; ***FERT***, Facial Expression Recognition Task; ***FOE***, The Face of Emotions; ***HCs***, Healthy Controls; ***ICD-10*** - International Classification of Diseases; ***ISI***, Interstimulus Interval; ***JACFEE*** - Japanese and Caucasian Facial Expressions of Emotion; ***KAMT***, Kinney’s Affect Matching Test; ***KDEF***, Karolinska Directed Emotional Faces; ***K-SADS-PL*** - Kiddie Schedule for Affective Disorders and Schizophrenia, present and lifetime version; ***Mini-SEA***, mini-Social Cognition and Emotional Assessment; ***METT***, Micro-expression Training Tool; ***MIMI***, MIMI Facial Expression Database; ***NA***, Not available; ***NOS*** - Newcastle-Ottawa Scale; ***PEAT***, Penn Emotion Acuity Test; **PIs**, Prediction Intervals; ***POFA***, Pictures of Facial Affect; ***SCAN*** - Schedules for Clinical Assessment in Neuropsychiatry; ***SCID*** - Structured Clinical Interview for DSM Disorders; ***SCID-P*** -Structured Clinical Interview for DSM Disorders-Patient version; ***SETT***, Subtle Expression Training Tool; ***SMD***, Standardized Mean Difference; ***TASIT***, The Awareness of Social Inference Test; ***TRENDS***, Tool for Recognition of Emotions in Neuropsychiatric Disorders; ***VERT-K***, Vienna Emotion Recognition Tasks; ***YMRS***, Young Mania Rating Scale; ***WASH-U-KSADS*** - Washington University Schedule for Affective Disorders and Schizophrenia.

## Deviations from the protocol

In the original protocol:

1. We planned to examine differences between people with BD and clinical populations, unaffected FDRs, and HCs.

However, due to the large number of results, we decided to present these comparisons in two different research reports to allow for a deeper and more focused discussion and clearer presentation of results. The comparisons between people with BD and other clinical populations have been previously published (doi: 10.1016/j.pnpbp.2023.110847).

2. We planned to perform a cumulative analysis to examine the effect of the publication year on our results.

However, we decided to use the meta-regressions instead to explore this association to provide the reader with a more uniform presentation of our results.

3. We planned to conduct meta-regression analyses based on the following predictors: primary, or secondary outcome, mean age, % of females, % of euthymic, % of BD-I, % of depressed, % of (hypo)manic, symptoms severity scale, age at onset, and duration of illness.

However, we decided to add additional predictors (years of education, duration of the stimulus, duration of the inter-stimulus interval, maximum emotion intensity, minimum emotion intensity, presence of morphing, number of emotions considered in the whole task, number of stimuli, presence of practice, and presence of neutral faces), to better explore the heterogeneity in our analyses.

4. We planned to conduct sensitivity analyses by i) removing one study at a time from the analysis and ii) considering only good-quality studies.

However, we decided to conduct another sensitivity analysis by removing those studies whose lower-level data were calculated from higher-level information, to further explore the robustness of our results.

## MOOSE Checklist

**eTable n. 1 - MOOSE Checklist**

| **Item No** | **Recommendation** | **Reported on Page No** |
| --- | --- | --- |
| Reporting of background should include | | |
| 1 | Problem definition | 3 |
| 2 | Hypothesis statement | 3-4 |
| 3 | Description of study outcome(s) | 4 |
| 4 | Type of exposure or intervention used | 3-4 |
| 5 | Type of study designs used | 5 |
| 6 | Study population | 3-4 |
| Reporting of search strategy should include | | |
| 7 | Qualifications of searchers (eg, librarians and investigators) | Supplement (Appendix II) |
| 8 | Search strategy, including time period included in the synthesis and key words | 5, Supplement (Appendix II) |
| 9 | Effort to include all available studies, including contact with authors | 5 |
| 10 | Databases and registries searched | 5 |
| 11 | Search software used, name and version, including special features used (eg, explosion) | Supplement (Appendix II) |
| 12 | Use of hand searching (eg, reference lists of obtained articles) | 5 |
| 13 | List of citations located and those excluded, including justification | Supplement (Appendix III) |
| 14 | Method of addressing articles published in languages other than English | 5 |
| 15 | Method of handling abstracts and unpublished studies | 5 |
| 16 | Description of any contact with authors | NA |
| Reporting of methods should include | | |
| 17 | Description of relevance or appropriateness of studies assembled for assessing the hypothesis to be tested | 5 |
| 18 | Rationale for the selection and coding of data (eg, sound clinical principles or convenience) | 5 |
| 19 | Documentation of how data were classified and coded (eg, multiple raters, blinding and interrater reliability) | 5 |
| 20 | Assessment of confounding (eg, comparability of cases and controls in studies where appropriate) | 6-7 |
| 21 | Assessment of study quality, including blinding of quality assessors, stratification or regression on possible predictors of study results | 5-7 |
| 22 | Assessment of heterogeneity | 6-7 |
| 23 | Description of statistical methods (eg, complete description of fixed or random effects models, justification of whether the chosen models account for predictors of study results, dose-response models, or cumulative meta-analysis) in sufficient detail to be replicated | 6-7 |
| 24 | Provision of appropriate tables and graphics | Figure 1-2, Table 1-3 |
| Reporting of results should include | | |
| 25 | Graphic summarizing individual study estimates and overall estimate | Figure 2, Table 2 |
| 26 | Table giving descriptive information for each study included | Table 1 |
| 27 | Results of sensitivity testing (eg, subgroup analysis) | 9-10, Supplement (Appendix VI) |
| 28 | Indication of statistical uncertainty of findings | 8-10, Table 2, Supplement (Appendix VI) |
| Reporting of discussion should include | | |
| 29 | Quantitative assessment of bias (eg, publication bias) | 10, Supplement (Appendix VI) |
| 30 | Justification for exclusion (eg, exclusion of non-English language citations) | 5 |
| 31 | Assessment of quality of included studies | 8, Supplement (Appendix V) |
| Reporting of conclusions should include | | |
| 32 | Consideration of alternative explanations for observed results | 11-14 |
| 33 | Generalization of the conclusions (ie, appropriate for the data presented and within the domain of the literature review) | 11-14 |
| 34 | Guidelines for future research | 11-14 |
| 35 | Disclosure of funding source | 15-16 |

# APPENDIX II

## Search strategy

Two investigators (MDP, VO) independently searched the databases. The software RAYYAN (<https://www.rayyan.ai>) was used for the screening process.

**PubMed/MEDLINE (inception to 28.03.24: 686 results)**

("surprise"[Title/Abstract] OR "fearful faces"[Title/Abstract] OR "disgust"[Title/Abstract] OR "happy faces"[Title/Abstract] OR "sad faces"[Title/Abstract] OR "angry faces"[Title/Abstract] OR "facial"[Title/Abstract] OR "facial expression"[Title/Abstract] OR "facial emotion"[Title/Abstract] OR "facial expressions"[Title/Abstract] OR "facial emotions"[Title/Abstract] OR "facial recognition"[MeSH Major Topic]) AND ("bipolar"[Title/Abstract] OR "bipolar disorder"[Title/Abstract] OR "mania"[Title/Abstract] OR "hypomania"[Title/Abstract] OR "manic"[Title/Abstract] OR "hypomanic"[Title/Abstract] OR "bipolar depression"[Title/Abstract] OR "euthymia"[Title/Abstract] OR "euthymic"[Title/Abstract] OR "bipolar disorder"[MeSH Terms])

**Scopus (inception to 28.03.24: 959 results)**

(TITLE-ABS(surprise) OR TITLE-ABS("fearful faces") OR TITLE-ABS(disgust) OR TITLE-ABS("happy faces") OR TITLE-ABS("sad faces") OR TITLE-ABS("angry faces") OR TITLE-ABS(facial) OR TITLE-ABS("facial expression") OR TITLE-ABS("facial emotion") OR TITLE-ABS("facial expressions") OR TITLE-ABS("facial emotions") OR INDEXTERMS("facial recognition")) AND (TITLE-ABS(bipolar) OR TITLE-ABS("bipolar disorder") OR TITLE-ABS(mania) OR TITLE-ABS(hypomania) OR TITLE-ABS(manic) OR TITLE-ABS(hypomanic) OR TITLE-ABS("bipolar depression") OR TITLE-ABS(euthymia) OR TITLE-ABS(euthymic) OR INDEXTERMS("bipolar disorder"))

**EMBASE (inception to 28.03.24: 1,237 results)**

(surprise:ti,ab OR 'fearful faces':ti,ab OR disgust:ti,ab OR 'happy faces':ti,ab OR 'sad faces':ti,ab OR 'angry faces':ti,ab OR facial:ti,ab OR 'facial expression':ti,ab OR 'facial emotion':ti,ab OR 'facial expressions':ti,ab OR 'facial emotions':ti,ab OR 'facial recognition'/exp/mj) AND (bipolar:ti,ab OR 'bipolar disorder':ti,ab OR mania:ti,ab OR hypomania:ti,ab OR manic:ti,ab OR hypomanic:ti,ab OR 'bipolar depression':ti,ab OR euthymia:ti,ab OR euthymic:ti,ab OR 'bipolar disorder'/exp)

**PsycINFO (inception to 28.03.24: 496 results)**

(TI(surprise) OR TI(fearful faces) OR TI(disgust) OR TI(happy faces) OR TI(sad faces) OR TI(angry faces) OR TI(facial) OR TI(facial expression) OR TI(facial emotion) OR TI(facial expressions) OR TI(facial emotions) OR TI(facial recognition) OR AB(surprise) OR AB(fearful faces) OR AB(disgust) OR AB(happy faces) OR AB(sad faces) OR AB(angry faces) OR AB(facial) OR AB(facial expression) OR AB(facial emotion) OR AB(facial expressions) OR AB(facial emotions) OR AB(facial recognition)) AND (TI(bipolar) OR TI(bipolar disorder) OR TI(mania) OR TI(hypomania) OR TI(manic) OR TI(hypomanic) OR TI(bipolar depression) OR TI(euthymia) OR TI(euthymic) OR AB(bipolar) OR AB(bipolar disorder) OR AB(mania) OR AB(hypomania) OR AB(manic) OR AB(hypomanic) OR AB(bipolar depression) OR AB(euthymia) OR AB(euthymic)

## Graphical representation of the three-level scheme used

**eFigure n.1 - Graphical representation of the three-level scheme used**

# APPENDIX III

## Excluded studies, with reasons

**eTable n. 2 - Excluded studies with reasons**

| **Author, year** | **Reason** |
| --- | --- |
| Almeida, 2009 | Same sample of another study |
| Almeida, 2009 | Same sample of another study |
| Almeida, 2017 | Wrong publication type |
| Almeida, 2011 | Wrong publication type |
| Anand, 2014 | Wrong publication type |
| Andrews, 2023 | Unrelated to our research question |
| Asch, 2022 | Wrong publication type |
| Bae, 2021 | Wrong publication type |
| Bauer, 2017 | Wrong publication type |
| Bauer, 2017 | Wrong publication type |
| Bauer, 2018 | Wrong publication type |
| Behere, 2016 | Wrong publication type |
| Berchio, 2019 | Unrelated to our research question |
| Bertocci, 2012 | Unrelated to our research question |
| Bio, 2017 | Wrong publication type |
| Bio, 2013 | Wrong publication type |
| Bio, 2012 | Wrong publication type |
| Bjertrup, 2019 | Wrong publication type |
| Blond, 2012 | Wrong publication type |
| Blumberg, 2005 | Unrelated to our research question |
| Broch-Due, 2017 | Wrong publication type |
| Brotman, 2012 | Wrong publication type |
| Brotman, 2014 | Wrong publication type |
| Brotman, 2011 | Wrong publication type |
| Brotman, 2014 | Unrelated to our research question |
| Chen, 2006 | Unrelated to our research question |
| Corrigan, 2020 | Wrong publication type |
| David, 2013 | Wrong publication type |
| Degabriele, 2009 | Wrong publication type |
| Degabriele, 2011 | Unrelated to our research question |
| Dernovsek, 2010 | Wrong publication type |
| Diler, 2013 | Unrelated to our research question |
| Diler, 2013 | Unrelated to our research question |
| Dima, 2017 | Wrong publication type |
| Dima, 2013 | Same sample of another study |
| Espinos, 2018 | Wrong publication type |
| Ford, 2012 | Wrong publication type |
| Fournier, 2013 | Wrong publication type |
| Fournier, 2011 | Wrong publication type |
| Frandsen, 2021 | Wrong publication type |
| Frangou, 2010 | Wrong publication type |
| Frangou, 2012 | Wrong publication type |
| Frangou, 2012 | Wrong publication type |
| Garrett, 2012 | Unrelated to our research question |
| Getz, 2004 | Wrong publication type |
| Goghari, 2011 | Wrong publication type |
| Green, 2010 | Wrong publication type |
| Gruber, 2024 | Wrong publication type |
| Haldane, 2009 | Wrong publication type |
| Hanford, 2011 | Wrong publication type |
| Hassel, 2010 | Wrong publication type |
| Hassel, 2009 | Same sample of another study |
| Hummer, 2013 | Wrong outcomes |
| Jensen, 2021 | Same sample of another study |
| Kempton, 2010 | Wrong publication type |
| Kieseppä, 2005 | Wrong outcomes |
| Kim, 2011 | Wrong publication type |
| Kim, 2012 | Wrong outcomes |
| Kjærstad, 2021 | Wrong publication type |
| Kjærstad, 2020 | Same sample of another study |
| Kjærstad, 2020 | Unrelated to our research question |
| Kjærstad, 2023 | Unrelated to our research question |
| Koussis, 2023 | Wrong population |
| Lackner, 2016 | Wrong publication type |
| Ladouceur, 2010 | Wrong publication type |
| Ladouceur, 2011 | Wrong outcomes |
| Ladouceur, 2010 | Wrong publication type |
| Lahera, 2015 | Wrong publication type |
| Lee, 2013 | Wrong publication type |
| Lee, 2011 | Wrong publication type |
| Leibenluft, 2009 | Wrong publication type |
| Leibenluft, 2015 | Wrong publication type |
| Lelli-Chiesa, 2010 | Wrong publication type |
| Lennox, 2004 | Wrong outcomes |
| Leyman, 2009 | Wrong outcomes |
| Li, 2019 | Same sample of another study |
| Liu, 2023 | Wrong outcomes |
| Long, 2012 | Wrong publication type |
| Lowes, 2013 | Wrong publication type |
| Ma, 2018 | Wrong outcomes |
| Manelis, 2020 | Wrong outcomes |
| Marchand, 2011 | Wrong outcomes |
| Marin, 2017 | Wrong publication type |
| McClure, 2003 | Same sample of another study |
| Meluken, 2017 | Wrong population |
| Menkes, 2022 | Wrong publication type |
| Monferrer, 2023 | Wrong publication type |
| Mullin, 2012 | Wrong outcomes |
| Mullin, 2009 | Wrong publication type |
| Narayanaswamy, 2013 | Wrong publication type |
| Olsavsky, 2012 | Wrong outcomes |
| Osório, 2018 | Wrong publication type |
| Passarotti, 2012 | Wrong outcomes |
| Passarotti, 2010 | Wrong outcomes |
| Passarotti, 2011 | Wrong outcomes |
| Pavuluri, 2010 | Wrong publication type |
| Perlman, 2013 | Wrong outcomes |
| Peters, 2016 | Wrong publication type |
| Phillips, 2009 | Wrong publication type |
| Phillips, 2011 | Wrong publication type |
| Phillips, 2011 | Wrong publication type |
| Priyesh, 2018 | Wrong publication type |
| Quide, 2018 | Wrong publication type |
| Rai, 2015 | Wrong publication type |
| Segar, 2021 | Wrong publication type |
| Almeida, 2010 | Wrong publication type |
| Rich, 2008 | Unrelated to our research question |
| Rootes-Murdy, 2017 | Wrong publication type |
| Rosenfeld, 2014 | Unrelated to our research question |
| Rossell, 2014 | Wrong publication type |
| Rowland, 2014 | Wrong publication type |
| Ruberto, 2010 | Wrong publication type |
| Russo, 2013 | Wrong publication type |
| Sapin, 1987 | No DSM/ICD criteria diagnosis |
| Saxena, 2017 | Wrong publication type |
| Schaefer, 2010 | Wrong publication type |
| Shankman, 2013 | Retracted article |
| Schenkel, 2009 | Wrong publication type |
| Schenkel, 2015 | Wrong publication type |
| Schenkel, 2011 | Wrong publication type |
| Simonetti, 2019 | Unrelated to our research question |
| Simonetti, 2023 | Unrelated to our research question |
| Sinha, 2018 | Wrong publication type |
| Steed, 2005 | Wrong publication type |
| Surguladze, 2010 | Unrelated to our research question |
| Tao, 2023 | Unrelated to our research question |
| Tehrani-Doost, 2013 | Wrong publication type |
| Thomas, 2014 | Unrelated to our research question |
| Thomas, 2012 | Unrelated to our research question |
| Tseng, 2016 | Unrelated to our research question |
| Unoka, 2011 | Wrong population |
| Van Rheenen, 2015 | Wrong publication type |
| Van Rheenen, 2017 | Wrong publication type |
| Van Rheenen, 2016 | Same sample of another study |
| Van Rheenen, 2014 | Wrong publication type |
| Vederman, 2010 | Wrong publication type |
| Vierck, 2012 | Wrong publication type |
| Wang, 2023 | Unrelated to our research question |
| Wiggins, 2016 | Same sample of another study |
| Wynn, 2011 | Wrong publication type |
| Yalcin, 2010 | Wrong publication type |
| Yılmaz, 2019 | Wrong population |
| Zeni, 2018 | Wrong publication type |

**References**

Almeida JR, Mechelli A, Hassel S, Versace A, Kupfer DJ, Phillips ML. Abnormally increased effective connectivity between parahippocampal gyrus and ventromedial prefrontal regions during emotion labeling in bipolar disorder. Psychiatry Research: Neuroimaging. 2009;174(3):195-201.

Almeida JR, Versace A, Mechelli A, et al. Abnormal amygdala-prefrontal effective connectivity to happy faces differentiates bipolar from major depression. Biol Psychiatry. Sep 1 2009;66(5):451-9. doi:10.1016/j.biopsych.2009.03.024

Almeida, J., Mourao-Miranda, J., Versace, A., Mechelli, A., Kupfer, D., & Phillips, M. (2017). Cerebral blood flow and connectivity differences between bipolar depression and unipolar depression.

Almeida, J. R. C. (2011). Connectivity and Cerebral Blood Flow Differences Between Bipolar Depression and Unipolar Depression. Biological psychiatry,

Anand, A. (2014). Neural Correlates of Sub-threshold Bipolarity. Biological psychiatry,

Andrews, C. M., Menkes, M. W., Suzuki, T., Lasagna, C. A., Chun, J., O'Donnell, L., Grove, T., McInnis, M. G., Deldin, P. J., & Tso, I. F. (2023). Reduced theta-band neural oscillatory activity during affective cognitive control in bipolar I disorder. Journal of psychiatric research, 158, 27-35.

Asch, R., Cool, R., Holmes, S., Davis, M., Carson, R., Worhunsky, P., Blumberg, H., & Esterlis, I. (2022). Using Multimodal Neuroimaging to Differentiate Depression in BD From MDD. Neuropsychopharmacology,

Bae, M. J., Jin, B. H., Rim, H.-D., & Won, S. (2021). Comparison of face emotional recognition deficits in remitted schizophrenia and euthymic bipolar I disorder. ASIA-PACIFIC PSYCHIATRY,

Bauer, I., Ramakrishnan, N., Ursu, S., Saxena, K., Zunta-Soares, G., Phillips, M., Spiker, D., & Soares, J. (2017a). 875. Differences in Neural Activation during Implicit Facial Emotion Processing in Youth and Adults with Bipolar Disorder. Biological psychiatry, 81(10), S353-S354.

Bauer, I. E., Ramakrishnan, N., Saxena, K., Ursu, S., Spiker, D., Kahlon, R., Arvind, R. P., Zunta-Soares, G., Phillips, M. L., & Kazimi, I. (2017b). Functional Activation During an Implicit Emotional Face Processing Task in Children and Adolecents With Bipolar Disorder and Unaffected Offspring of Bipolar Parents. 64th Annual Meeting,

Bauer, I. E., Zeni, C. P., Kazimi, I. F., Zunta-Soares, G., Kahlon, R. S., Saxena, K., Soares, J. C., Tannous, J., & Irungu, B. (2018). Brain Activation in Response to Fearful and Happy Stimuli: A Marker of Bipolar Disorder Across the Lifespan. 65th Annual Meeting,

Behere, R. V., UmeshTonse, Praharaj, S. K., & Sharma, P. (2016). Hemi Facial Emotion Recognition in Schizophrenia Versus Bipolar Disorder. INDIAN JOURNAL OF PSYCHIATRY,

Berchio, C., Küng, A.-L., Kumar, S., Cordera, P., Dayer, A. G., Aubry, J.-M., Michel, C. M., & Piguet, C. (2019). Eye-gaze processing in the broader bipolar phenotype revealed by electrical neuroimaging. Psychiatry Research: Neuroimaging, 291, 42-51.

Bertocci, M., Bebko, G., Mullin, B., Langenecker, S., Ladouceur, C., Almeida, J., & Phillips, M. L. (2012). Abnormal anterior cingulate cortical activity during emotional n-back task performance distinguishes bipolar from unipolar depressed females. Psychological medicine, 42(7), 1417-1428.

Bio, D., Monteiro, R., Soeiro-de-Souza, M., Moreno, D., & Moreno, R. (2013). The association between family functioning and childhood trauma and cognition in patients with bipolar disorder type I. Bipolar disorders,

Bio, D., Soeiro-de-Souza, M., Moreno, D., & Moreno, R. (2012). COMT allele (Met (158)) modulates facial emotion recognition in bipolar disorder I mood episodes and in healthy controls. Bipolar disorders,

Bio, D., Soeiro-de-Souza, M., & Moreno, R. (2017). The association between childhood trauma, cognitive and brain abnormalities in bipolar I patients. Bipolar disorders,

Bjertrup, A., Jensen, M. B., Schjødt, M. S., Moszkowicz, M., Vinberg, M., Mikkelsen, R. L., Kjærbye-Thygesen, A., Frøkjær, V., Kessing, L. V., & Væver, M. S. (2019). Hormones, Emotional processing and prepartum Attachment in Pregnant women with Affective Disorders (HEAPAD). European Neuropsychopharmacology,

Blond, B. (2012). Functional Magnetic Resonance Imaging Of State And Trait Abnormalities In Bipolar Disorder.

Blumberg, H. P., Donegan, N. H., Sanislow, C. A., Collins, S., Lacadie, C., Skudlarski, P., Gueorguieva, R., Fulbright, R. K., McGlashan, T. H., & Gore, J. C. (2005). Preliminary evidence for medication effects on functional abnormalities in the amygdala and anterior cingulate in bipolar disorder. Psychopharmacology, 183, 308-313.

Broch-Due, I., Kjærstad, H. L., Kessing, L. V., & Miskowiak, K. W. (2017). Increased facial expressiveness in bipolar patients when viewing neutral images: A pilot study. Bipolar disorders,

Brotman, M., Tseng, W.-L., Olsavsky, A., Fromm, S., Muhrer, E., Rutenberg, J., Deveney, C., Adleman, N., Zarate, C., & Pine, D. (2014a). Fronto-limbic-striatal dysfunction in pediatric and adult patients with bipolar disorder: impact of face emotion and attentional demands. Psychological medicine, 44(8), 1639-1651.

Brotman, M. A., Bones, B. L., Olsavsky, A. K., Adleman, N. E., Deveney, C. M., Dickstein, D. P., Pine, D. S., & Leibenluft, E. (2012). Emotional face encoding in youth at risk for bipolar disorder: A preliminary fMRI study. Neurropsychopharmacology, 38, S259.

Brotman, M. A., Deveney, C. M., Thomas, L. A., Hinton, K. E., Yi, J. Y., Pine, D. S., & Leibenluft, E. (2014b). Parametric modulation of neural activity during face emotion processing in unaffected youth at familial risk for bipolar disorder. Bipolar disorders, 16(7), 756-763.

Brotman, M. A., Olsavsky, A. K., Rutenberg, J. G., Muhrer, E. J., Fromm, S. J., Zarate Jr, C. A., Pine, D. S., & Leibenluft, E. (2011). Amygdala and prefrontal dysfunction during face processing in pediatric and adult patients with bipolar disorder: Role of face emotion and attentional demands. Neuropsychipharmacology, 36, S387.

Chen, C.-H., Lennox, B., Jacob, R., Calder, A., Lupson, V., Bisbrown-Chippendale, R., Suckling, J., & Bullmore, E. (2006). Explicit and implicit facial affect recognition in manic and depressed states of bipolar disorder: a functional magnetic resonance imaging study. Biological psychiatry, 59(1), 31-39.

Corrigan, A. B., K. Gunning, F. (2020). Aging, attentional biases, and emotional regulation strategies in bipolar disorder. Bipolar disorders, 22, 79.

David, D., Bio, D., Soeiro-de-Souza, M., & Moreno, R. (2013). Facial emotion recognitions and its correlation to executive functions in young bipolar I patients. Bipolar disorders,

Degabriele, R., Lagopoulos, J., Coulston, C., & Malhi, G. (2009). Facing potential bipolar disorder? N170 event-related potential variances during emotional face processing in bipolar disorder. Bipolar disorders.

Degabriele, R., Lagopoulos, J., & Malhi, G. (2011). Neural correlates of emotional face processing in bipolar disorder: an event-related potential study. Journal of affective disorders, 133(1-2), 212-220.

Dernovsek, M., Novak, T., & Sprah, L. (2010). PW01-09-Assessment of cognitive functioning within different emotional contexts in the group of euthymic bipolar patients. European Psychiatry, 25(S1), 1-1.

Diler, R. S., de Almeida, J. R. C., Ladouceur, C., Birmaher, B., Axelson, D., & Phillips, M. (2013a). Neural activity to intense positive versus negative stimuli can help differentiate bipolar disorder from unipolar major depressive disorder in depressed adolescents: a pilot fMRI study. Psychiatry Research: Neuroimaging, 214(3), 277-284.

Diler, R. S., Ladouceur, C. D., Segreti, A., Almeida, J. R., Birmaher, B., Axelson, D. A., Phillips, M. L., & Pan, L. A. (2013b). Neural correlates of treatment response in depressed bipolar adolescents during emotion processing. Brain imaging and behavior, 7, 227-235.

Dima, D., Breen, G., & Frangou, S. (2017). The relationship between polygenic risk score for bipolar disorder and brain function during a facial affect labelling paradigm. European neuropsychopharmacology,

Dima, D., Jogia, J., Collier, D., Vassos, E., Burdick, K. E., & Frangou, S. (2013). Independent modulation of engagement and connectivity of the facial network during affect processing by CACNA1C and ANK3 risk genes for bipolar disorder. JAMA psychiatry, 70(12), 1303-1311.

Espinos, U. (2018). Facial emotion recognition in bipolar disorder. European Psychiatry,

Ford, K. A., Neufeld, R., Mitchell, D., Theberge, J., Williamson, P., & Osuch, E. (2012). Networks of Functional Brain Activation Differentiate Unipolar Major Depressive Disorder and Bipolar Disorders. Biological psychiatry,

Fournier, J. C., Chase, H. W., Almeida, J., & Phillips, M. L. (2013). Six-Month Stability in FMRI Markers of Emotion Processing in Depressed and Healthy Control Participants. Biological psychiatry,

Fournier, J. C., Keener, M. T., Mullin, B. C., Almeida, J. R., & Phillips, M. L. (2011). Responses to Particular Negative Emotional Stimuli Discriminate Bipolar from Unipolar Depression. Biological psychiatry,

Frandsen, S., DelDonno, S., Dillahunt, A., Pocius, S., Schubert, B., Kassel, M., Bessette, K., Thomas, L., Stange, J., & Jenkins, L. (2021). Sex Differences in Neural Activation During Emotional Processing of Subliminal and Supraliminal Faces. Biological psychiatry, 89(9), S178.

Frangou, S. (2010). The Functional Consequences of Risk Genes for Bipolar Disorder on Neural Function. Biological psychiatry, 67(9), 15S-16S.

Frangou, S. (2012a). CACNA1C as a Genetic Modifier of Brain Volume, Connectivity and Function. Biological psychiatry,

Frangou, S. (2012b). The effect of CACNA1C rs1006737 polymorphism on brain structure, function and connectivity in BD. Bipolar disorders, 14, 28-29.

Garrett, A. S., Reiss, A. L., Howe, M. E., Kelley, R. G., Singh, M. K., Adleman, N. E., Karchemskiy, A., & Chang, K. D. (2012). Abnormal amygdala and prefrontal cortex activation to facial expressions in pediatric bipolar disorder. Journal of the American Academy of Child & Adolescent Psychiatry, 51(8), 821-831.

Getz, G. E. (2004). Social functioning and facial affect recognition deficits in mood disorders. University of Cincinnati.

Goghari, V. M., & Sponheim, S. R. (2011). Differential Deficits in Facial Emotion Recognition in Schizophrenia and Bipolar Disorder. Biological psychiatry,

Green, M. J., Sparks, A., Lino, B. J., McDonald, S., & Mitchell, P. B. (2010). Social cognitive endophenotypes in bipolar disorder and schizophrenia? Bipolar disorders, 12, 23.

Gruber, J., Fischer, J., Page-Gould, E., & Johnson, S. L. (2024). Too close for comfort? Social distance and emotion perception in remitted bipolar I disorder. Journal of Social and Clinical Psychology, 43(1), 1-27.

Haldane, M., Kempton, M., & Frangou, S. (2009). Ventral prefrontal function mediates resileince to bipolar disorder: An fMRI study of BD patients and their unaffected siblings. European Psychiatry, 24(S1), 1-1.

Hanford, L., Frey, B., Hall, G., & Sassi, R. B. (2011). Emotional Conflict in Offspring of Bipolar Parents-Preliminary fMRI Findings. Biological psychiatry,

Hassel, S., Almeida, J., Versace, A., Kupfer, D. J., & Phillips, M. L. (2010). Bipolar and Unipolar Depression are Distinguished by Patterns of Abnormal Occipital Activity to Happy and Sad Facial Expressions. Biological psychiatry,

Hassel, S., Almeida, J. R., Frank, E., Versace, A., Nau, S. A., Klein, C. R., Kupfer, D. J., & Phillips, M. L. (2009). Prefrontal cortical and striatal activity to happy and fear faces in bipolar disorder is associated with comorbid substance abuse and eating disorder. Journal of affective disorders, 118(1-3), 19-27.

Hummer, T. A., Hulvershorn, L. A., Karne, H. S., Gunn, A. D., Wang, Y., & Anand, A. (2013). Emotional response inhibition in bipolar disorder: a functional magnetic resonance imaging study of trait-and state-related abnormalities. Biological psychiatry, 73(2), 136-143.

Jensen, M. B., Kjærstad, H. L., Coello, K., Stanislaus, S., Melbye, S., Sletved, K. O., Vinberg, M., Kessing, L. V., & Miskowiak, K. W. (2021). Affective and non-affective cognition in patients with bipolar disorder type I and type II in full or partial remission: associations with familial risk. Journal of affective disorders, 283, 207-215.

Kempton, M., Dima, D., Roiser, J., Stephan, K., Friston, K. J., & Frangou, S. (2010). Effective Connectivity within the Network of Fearful Facial Affect Recognition in Patients with Bipolar Disorder Compared to Healthy Controls.

Kieseppä, T., Tuulio-Henriksson, A., Haukka, J., Van Erp, T., Glahn, D., Cannon, T. D., Partonen, T., Kaprio, J., & Lönnqvist, J. (2005). Memory and verbal learning functions in twins with bipolar-I disorder, and the role of information-processing speed. Psychological medicine, 35(2), 205-215.

Kim, P., Arizpe, J., Rosen, B. H., Razdan, V., Haring, C. T., Jenkins, S. E., Deveney, C. M., Brotman, M. A., Blair, R. J. R., & Pine, D. S. (2013). Impaired fixation to eyes during facial emotion labelling in children with bipolar disorder or severe mood dysregulation. Journal of Psychiatry and Neuroscience, 38(6), 407-416.

Kim, P., Thomas, L. A., Rosen, B. H., Moscicki, A. M., Brotman, M. A., Zarate, J., Carlos A, Blair, R. J. R., Pine, D. S., & Leibenluft, E. (2012). Differing amygdala responses to facial expressions in children and adults with bipolar disorder. American Journal of Psychiatry, 169(6), 642-649.

Kim, T. S., Lee, S. Y., Ha, R. Y., Kim, E., Kyoon, S., Ha, K., & Cho, H.-S. (2011). Emotional priming with facial exposures in euthymic patients with bipolar disorder. The Journal of nervous and mental disease, 199(12), 971-977.

Kjærstad, H. L., Eikeseth, F. F., Vinberg, M., Kessing, L. V., & Miskowiak, K. (2021). Neurocognitive heterogeneity in patients with bipolar disorder and their unaffected relatives: associations with emotional cognition. Psychological medicine, 51(4), 668-679.

Kjærstad, H. L., Mistarz, N., Coello, K., Stanislaus, S., Melbye, S. A., Harmer, C. J., Vinberg, M., Miskowiak, K., & Kessing, L. V. (2020). Aberrant cognition in newly diagnosed patients with bipolar disorder and their unaffected relatives. Psychological medicine, 50(11), 1808-1819.

Kjærstad, H. L., Jørgensen, C. K., Broch-Due, I., Kessing, L. V., & Miskowiak, K. (2020). Eye gaze and facial displays of emotion during emotional film clips in remitted patients with bipolar disorder. European Psychiatry, 63(1), e29.

Kjærstad, H. L., Varo, C., Meluken, I., Vieta, E., Vinberg, M., Kessing, L. V., & Miskowiak, K. W. (2023). Emotional cognition subgroups in unaffected first-degree relatives of patients with mood disorders. Psychological Medicine, 53(6), 2328-2338.

Koussis, N. C., Burgher, B., Jeganathan, J., Scott, J. G., Cocchi, L., & Breakspear, M. (2023). Cognitive Control System Gates Insula Processing of Affective Stimuli in Early Psychosis. Schizophrenia Bulletin, sbad010.

Lackner, N., Birner, A., Bengesser, S., Fellendorf, F., Platzer, M., Queissner, R., Kapfhammer, H., Reininghaus, B., Weiss, E., & Wallner-Liebmann, S. (2016). Social cognition deficits in bipolar disorder–associations with overweight and obesity. European Neuropsychopharmacology, 2(26), S438-S439.

Ladouceur, C. D., Farchione, T., Diwadkar, V., Pruitt, P., Radwan, J., Axelson, D. A., Birmaher, B., & Phillips, M. L. (2011). Differential patterns of abnormal activity and connectivity in the amygdala–prefrontal circuitry in bipolar-I and bipolar-NOS youth. Journal of the American Academy of Child & Adolescent Psychiatry, 50(12), 1275-1289. e1272.

Ladouceur, C. D., Farchione, T., Pruitt, P., Diwadkar, V., Axelson, D., Birmaher, B., & Phillips, M. (2010a). Reduced Lateral Prefrontal Cortex Activation During an Emotional Working Memory Task in Pediatric Bipolar Disorder. Biological psychiatry,

Ladouceur, C., Pruitt, P., Diwadkar, V., Farchione, T., Axelson, D., Birmaher, B., Radwan, J., Phillips, M. (2010b). Differential patterns of abnormal neural activity and functional connectivity to emotional faces in bipolar and bipolar NOS youth. Poster Session I - Neuropsychopharmacology, 35(1), S78-S187. https://doi.org/10.1038/npp.2010.216

Lahera, G. (2015). Social cognitive impairments in schizophrenia and bipolar disorder. 5th European Conference on Schizophrenia Research: bridging gaps—improving outcomes.

Lee, J., Altshuler, L., Glahn, D. C., Miklowitz, D. J., Ochsner, K., & Green, M. F. (2013). Social and nonsocial cognition in bipolar disorder and schizophrenia: relative levels of impairment. American Journal of Psychiatry, 170(3), 334-341.

Lee, J. W., Wynn, J. K., Altshuler, L., Glahn, D., Horan, W. P., Green, M. F. (2011). Social cognitive performance profile of bipolar disorder and schizophrenia. ACNP 50th Annual Meeting Poster Abstracts-Neuropsychopharmacology, 36, S75-S197.

Leibenluft, E. (2009). Behavioral Endophenotypes in Bipolar Disorder. Biological psychiatry,

Leibenluft, E. (2015). Do the Neural Mechanisms Mediating Irritability Differ Across Diagnoses? Neuropsychopharmacology,

Lelli-Chiesa, G., Kempton, M., Jogia, J., & Frangou, S. (2010). P02-152-Dissociable limbic and prefrontal neural responses to sad facial expressions mediate disease expression and resilience in bipolar disorder. European Psychiatry, 25(S1), 1-1.

Lennox, B., Jacob, R., Calder, A., Lupson, V., & Bullmore, E. (2004). Behavioural and neurocognitive responses to sad facial affect are attenuated in patients with mania. Psychological medicine, 34(5), 795-802.

Leyman, L., De Raedt, R., & Koster, E. (2009). Attentional biases for emotional facial stimuli in currently depressed patients with bipolar disorder. International journal of clinical and health psychology, 9(3), 393-410.

Li L, Ji E, Tang F, et al. Abnormal brain activation during emotion processing of euthymic bipolar patients taking different mood stabilizers. Brain imaging and behavior. 2019;13:905-913.

Liu, S., Jia, Y., Liu, X., Ma, R., Zheng, S., Zhu, H., Yin, M., & Jia, H. (2023). Variation in self and familiar facial recognition in bipolar disorder patients at different clinical stages. Acta Psychologica, 235, 103903.

Long, E. A. (2012). Facial Affect Recognition and Interpretation in Adolescents At Risk for Developing Bipolar Disorder. University of Cincinnati.

Lowes, A. S., Jacobs, R. H., & Pavuluri, M. N. (2013). Do Youth with ADHD Have Similar or Different Face-Processing Deficits When Compared to Youth with PBD? Biological psychiatry,

Ma, G., Wang, C., Jia, Y., Wang, J., Zhang, B., Shen, C., Fan, H., Pan, B., & Wang, W. (2018). Electrocardiographic and electrooculographic responses to external emotions and their transitions in bipolar I and II disorders. International journal of environmental research and public health, 15(5), 884.

Manelis, A., Iyengar, S., Swartz, H. A., & Phillips, M. L. (2020). Prefrontal cortical activation during working memory task anticipation contributes to discrimination between bipolar and unipolar depression. Neuropsychopharmacology, 45(6), 956-963.

Marchand, W. R., Lee, J. N., Garn, C., Thatcher, J., Gale, P., Kreitschitz, S., Johnson, S., & Wood, N. (2011). Aberrant emotional processing in posterior cortical midline structures in bipolar II depression. Progress in Neuro-Psychopharmacology and Biological Psychiatry, 35(7), 1729-1737.

Marin, A., Munoz, D., & Soncin, S. (2017). Can eye movements differentiate between adult patients with ADHD and bipolar disorder? 6th World Congress on ADHD: From Child to Adult Disorder.

McClure, E. B., Pope, K., Hoberman, A. J., Pine, D. S., & Leibenluft, E. (2003). Facial expression recognition in adolescents with mood and anxiety disorders. American Journal of Psychiatry, 160(6), 1172-1174.

Meluken, I., Ottesen, N. M., Vedel, K. L., Maj, V., & Woznica, M. K. (2017). Nerocognitive response to emotional faces in monozygotic twins: association with genetic risk for affective disorder. Bipolar disorders,

Menkes, M. W., Andrews, C. M., Suzuki, T., Chun, J., O'Donnell, L., Grove, T., Deng, W., McInnis, M. G., Deldin, P. J., & Tso, I. F. (2022). Event-related potential correlates of affective response inhibition in bipolar I disorder: Comparison with schizophrenia. Journal of affective disorders, 309, 131-140.

Monferrer, M., García, A. S., Ricarte, J. J., Montes, M. J., Fernández-Caballero, A., & Fernández-Sotos, P. (2023). Dynamic virtual faces demonstrate deterioration in the recognition of facial emotion in bipolar disorder patients. Current Psychology, 1-12.

Mullin, B., Versace, A., Ladouceur, C., & Phillips, M. (2009). Increased dorsolateral prefrontal cortex activation in euthymic bipolar adults during performance of an emotional working memory task. Bipolar disorders,

Mullin, B. C., Perlman, S. B., Versace, A., de Almeida, J. R., LaBarbara, E. J., Klein, C., Ladouceur, C. D., & Phillips, M. L. (2012). An fMRI study of attentional control in the context of emotional distracters in euthymic adults with bipolar disorder. Psychiatry Research: Neuroimaging, 201(3), 196-205.

Narayanaswamy, J. (2013). A study of emotion processing strategies in bipolar disorder: novel evidence for the endophenotype status of labeling deficit. Bipolar disorders,

Olsavsky, A. K., Brotman, M. A., Rutenberg, J. G., Muhrer, E. J., Deveney, C. M., Fromm, S. J., Towbin, K., Pine, D. S., & Leibenluft, E. (2012). Amygdala hyperactivation during face emotion processing in unaffected youth at risk for bipolar disorder. Journal of the American Academy of Child & Adolescent Psychiatry, 51(3), 294-303.

Osório, F. d. L., & Dualibe, A. (2018). Recognition of facial emotion: a possible endophenotype for bipolar disorder? European Psychiatry, 48, S253.

Passarotti, A. M., Ellis, J., Wegbreit, E., Stevens, M. C., & Pavuluri, M. N. (2012). Reduced functional connectivity of prefrontal regions and amygdala within affect and working memory networks in pediatric bipolar disorder. Brain connectivity, 2(6), 320-334.

Passarotti, A. M., Sweeney, J. A., & Pavuluri, M. N. (2010). Emotion processing influences working memory circuits in pediatric bipolar disorder and attention-deficit/hyperactivity disorder. Journal of the American Academy of Child & Adolescent Psychiatry, 49(10), 1064-1080.

Passarotti, A. M., Sweeney, J. A., & Pavuluri, M. N. (2011). Fronto-limbic dysfunction in mania pre-treatment and persistent amygdala over-activity post-treatment in pediatric bipolar disorder. Psychopharmacology, 216, 485-499.

Pavuluri, M., Passarotti, A., Fitzgerald, J., & Sweeney, J. (2010). Differential impact of risperidone and divalproex in modulating negative and positive emotions during an affective working memory task in pediatric mania. American College of Neuropsychopharmacology 29th Annual meeting. , Miami, Florida.

Perlman, S. B., Fournier, J. C., Bebko, G., Bertocci, M. A., Hinze, A. K., Bonar, L., Almeida, J. R., Versace, A., Schirda, C., & Travis, M. (2013). Emotional face processing in pediatric bipolar disorder: evidence for functional impairments in the fusiform gyrus. Journal of the American Academy of Child & Adolescent Psychiatry, 52(12), 1314-1325. e1313.

Phillips, M. L. (2009). Abnormal structural and functional orbitomedial prefronto-limbic connectivity during emotion processing in adult bipolar disorder Clinical Application of Cognitive and Affective Neuroscience to Developmental Problems,

Phillips, M. L. (2011a). Differential Patterns of Emotion Regulation Neural Circuitry Abnormalities Distinguish Bipolar and Unipolar Depression. Biological psychiatry,

Phillips, M. L. (2011b). Neuroimaging Biomarkers of Future Bipolar Disorder in Clinically and Genetically At Risk Youth. Biological psychiatry,

Priyesh, C., Nayak, K., Suryavanshi, C., Bhandary, R., Behere, R., Sasidharan, A., & Kamath, A. (2018). ERP based Differences in Facial Emotion Recognition. https://doi.org/10.13140/RG.2.2.15277.74724

Quide, Y., Cohen-Woods, S., O’Reilly, N., Carr, V., Elzinga, B., & Green, M. (2018). S59. Childhood trauma is associated with social cognition and schizotypal personality traits in psychotic and healthy populations. Schizophrenia Bulletin, 44(Suppl 1), S347-S347.

Rai, N., Narayan, S., Sawa, A., Lin, S. Y., McLean, C. K., Hipolito, M. S., Cascella, N., Nurnberger, J. J., Ishizuka, K., & Nwulia, E. (2015). Olfactory Neuronal Expression of CRMP1 Is Associated with Odor and Emotional Processing. Biological psychiatry,

Rich, B. A., Fromm, S. J., Berghorst, L. H., Dickstein, D. P., Brotman, M. A., Pine, D. S., & Leibenluft, E. (2008). Neural connectivity in children with bipolar disorder: impairment in the face emotion processing circuit. Journal of Child Psychology and Psychiatry, 49(1), 88-96.

Rootes-Murdy, K., Glazer, K., Goes, F., Mondimore, F., Zandi, P., DePaulo Jr, J. R., Bakker, A., & Mahon, P. (2017). fMRI Correlates of Lithium Response: A Pilot Study in Bipolar Disorder. Neuropsychopharmacology,

Rosenfeld, E. S., Pearlson, G. D., Sweeney, J. A., Tamminga, C. A., Keshavan, M. S., Nonterah, C., & Stevens, M. C. (2014). Prolonged hemodynamic response during incidental facial emotion processing in inter-episode bipolar I disorder. Brain imaging and behavior, 8, 73-86.

Rossell, S. L., Van Rheenen, T. E., Joshua, N. R., O’Regan, A., & Gogos, A. (2014). Investigating facial affect processing in psychosis: A study using the Comprehensive Affective Testing System. Schizophrenia research, 157(1-3), 55-59.

Rowland, J. E., O'Reilly, N., Girshkin, L., Mitchell, P. B., Carr, V. J., & Green, M. J. (2014). Poster# T202 SOCIAL COGNITION IN NEUROCOGNITIVE SUBTYPES OF PSYCHOTIC DISORDERS. Schizophrenia research(153), S361.

Ruberto, G., Lelli-Chiesa, G., Vassos, E., Maierú, M., Tatarelli, R., Girardi, P., Collier, D., Kempton, M. J., & Frangou, S. (2010). P01-89 - Preliminary Analysis of the Functional Consequences of the CACNA1C Gene Polymorphism on Emotional Processing in Bipolar Disorder. European Psychiatry, 25(S1), 25-E308, Article 25-e308. https://doi.org/10.1016/S0924-9338(10)70308-4

Russo, J. M., Spencer, L., Wang, F., & Blumberg, H. P. (2013). Amygdala Responses to Emotional Faces: A Possible Marker of Risk for Bipolar Disorder in Adolescents. Biological psychiatry,

Sapin, L. R., Berrettini, W. H., Nurnberger, J. I., & Rothblat, L. A. (1987). Mediational factors underlying cognitive changes and laterality in affective illness. Biological psychiatry, 22(8), 979-986. https://doi.org/https://doi.org/10.1016/0006-3223(87)90007-2

Saxena, K., & Soares, J. C. (2017). The Story of Risk and Resilience in Youth With Bipolar Disorder, High-Risk Youth, and Low-Risk Youth: Converging Evidence From Electroencephalogram, Neuroanatomy, Gene-Environment Interaction, and Brain Activation. 64th Annual Meeting,

Schaefer, K. L., Luckenbaugh, D. A., Baumann, J. C., & Zarate, C. A. (2010). Face Emotion in Adult Bipolar and Unipolar Depression and Controls. Biological psychiatry,

Shankman SA, Katz AC, Passarotti AM, Pavuluri MN. RETRACTED: Deficits in emotion recognition in pediatric bipolar disorder: The mediating effects of irritability. Elsevier; 2013.

Schenkel, L., Sweeney, J., & Pavuluri, M. (2009). Impact of emotional valiance on facial affect recognition and memory in pediatric bipolar disorder. Bipolar disorders,

Schenkel, L., Walders, K., Towne, T., LaJudice, C., Herbert, A., & Pelz, J. (2011). Atypical visual scanning of emotional faces in pediatric bipolar disorder using high speed remote eye tracking. Bipolar disorders,

Schenkel, L. S., Towne, T. L., Herbert, A. M., & Pelz, J. B. (2015). Errors in identifying emotion in body postures and facial expressions among youth with pediatric bipolar disorder. Bipolar disorders, 17, 107.

Simonetti, A., Lijffijt, M., Kahlon, R. S., Gandy, K., Arvind, R. P., Amin, P., Arciniegas, D. B., Swann, A. C., Soares, J. C., & Saxena, K. (2019). Early and late cortical reactivity to passively viewed emotional faces in pediatric bipolar disorder. Journal of affective disorders, 253, 240-247. <https://doi.org/https://doi.org/10.1016/j.jad.2019.04.076>

Simonetti, A., Lijffijt, M., Kurian, S., Saxena, J., Janiri, D., Mazza, M., ... & Soares, J. C. (2023). Neuroanatomical Correlates of the Late Positive Potential in Youth with Pediatric Bipolar Disorder. Current Neuropharmacology, 21(7), 1617.

Sinha, S. K. (2018). Neural Correlate of Emotion Recognition in Bipolar Disorder and Schizophrenia: A Comparative Study Central Institute of Psychiatry (India)].

Steed, M. A. (2005). Facial affect recognition in bipolar disorder: A functional magnetic resonance imaging study. University of Cincinnati.

Surguladze, S. A., Marshall, N., Schulze, K., Hall, M. H., Walshe, M., Bramon, E., Phillips, M. L., Murray, R. M., & McDonald, C. (2010). Exaggerated neural response to emotional faces in patients with bipolar disorder and their first-degree relatives. Neuroimage, 53(1), 58-64. https://doi.org/https://doi.org/10.1016/j.neuroimage.2010.05.069

Tao, P., Dai, Z., Shao, J., Tang, H., Zhang, S., Yao, Z., & Lu, Q. (2023). Gamma band VMPFC-PreCG.L connection variation after the onset of negative emotional stimuli can predict mania in depressive patients. Journal of Psychiatric Research, 158, 165-171. https://doi.org/https://doi.org/10.1016/j.jpsychires.2022.12.026

Tehrani-Doost, M., Bozorg, B., Shahrivar, Z., Fata, L., & Mohammadzadeh, A. (2013). The ability to recognize emotional faces and its relation to response inhibition and decision making in adolescents with bipolar disorder. Eur Child Adolesc Psychiatry 22, S141.

Thomas, L. A., Brotman, M. A., Bones, B. L., Chen, G., Rosen, B. H., Pine, D. S., & Leibenluft, E. (2014). Neural circuitry of masked emotional face processing in youth with bipolar disorder, severe mood dysregulation, and healthy volunteers. Developmental Cognitive Neuroscience, 8, 110-120. https://doi.org/https://doi.org/10.1016/j.dcn.2013.09.007

Thomas, L. A., Brotman, M. A., Muhrer, E. J., Rosen, B. H., Bones, B. L., Reynolds, R. C., Deveney, C. M., Pine, D. S., & Leibenluft, E. (2012). Parametric Modulation of Neural Activity by Emotion in Youth With Bipolar Disorder, Youth With Severe Mood Dysregulation, and Healthy Volunteers. Archives of General Psychiatry, 69(12), 1257-1266. https://doi.org/10.1001/archgenpsychiatry.2012.913

Tseng, W.-L., Thomas, L. A., Harkins, E., Stoddard, J., Zarate, C. A., Pine, D. S., Leibenluft, E., & Brotman, M. A. (2016). Functional connectivity during masked and unmasked face emotion processing in bipolar disorder. Psychiatry Research: Neuroimaging, 258, 1-9. https://doi.org/https://doi.org/10.1016/j.pscychresns.2016.10.006

Unoka, Z., Fogd, D., Füzy, M., & Csukly, G. (2011). Misreading the facial signs: Specific impairments and error patterns in recognition of facial emotions with negative valence in borderline personality disorder. Psychiatry research, 189(3), 419-425. https://doi.org/https://doi.org/10.1016/j.psychres.2011.02.010

Van Rheenen, T. (2015). Behavioural indices of emotion processing in bipolar disorder 17th Annual Conference of the International Society for Bipolar Disorders,

Van Rheenen, T., & Rossell, S. (2016). Facial Emotion Recognition Impairments in Bipolar Disorder. A Cognitive Problem? Journal of the International Neuropsychological Society, 22(6), 583-585. https://doi.org/10.1017/S1355617716000394

Van Rheenen, T. E., Joshua, N., Castle, D. J., & Rossell, S. L. (2017). Configural and Featural Face Processing Influences on Emotion Recognition in Schizophrenia and Bipolar Disorder. Journal of the International Neuropsychological Society, 23(3), 287-291. https://doi.org/10.1017/S1355617716001211

Van Rheenen, T. E., & Rossell, S. L. (2014). Multimodal Emotion Integration in Bipolar Disorder: An Investigation of Involuntary Cross-Modal Influences between Facial and Prosodic Channels. Journal of the International Neuropsychological Society, 20(5), 525-533. https://doi.org/10.1017/S1355617714000253

Vederman, A. C., Leon, H. M., Wright, S. L., Haase, B., Huq, N., Hazlett, K. E., Giordani, B., Schteingart, D. E., Starkman, M. N., & Rapport, L. A. (2010). Visual Affect Recognition Accuracy Differences by Psychiatric Illness. Biological psychiatry,

Vierck, E., Porter, R., & Joyce, P. (2012). Effects of personality and gender on recognition of emotions in bipolar disorder. Bipolar disorders.

Wang, L.-j., Cheng, T.-h., Geng, J.-s., Yang, J., Liu, C., Zhu, G.-h., Luo, J.-c., Wang, G.-z., he Zhu, X., & Wang, Y. (2023). Comparisons of facial emotion recognition in different social contexts among patients with schizophrenia, major depressive disorder and bipolar disorder. Asian Journal of Psychiatry, 83, 103566.

Wiggins, J. L., Brotman, M. A., Adleman, N. E., Kim, P., Oakes, A. H., Reynolds, R. C., Chen, G., Pine, D. S., & Leibenluft, E. (2016). Neural correlates of irritability in disruptive mood dysregulation and bipolar disorders. American Journal of Psychiatry, 173(7), 722-730.

Wynn, J. K., Mathis, K. I., Jahshan, C., Altshuler, L., Glahn, D., & Green, M. F. (2011). Facial processing in bipolar disorder and schizophrenia: An event-related potential study Neuropsychopharmacology,

Yalcin, N., Hofer, A., Baumgartner, S., Benecke, C., Biedermann, F., Deisenhammer, E., Hausmann, A., Hörtnagl, C., Kemmler, G., & Muehlbacher, M. (2010). Facial affect recognition in remitted patients with schizophrenia and bipolar disorder I. Schizophrenia research, 117(2-3), 523-524.

Yılmaz, O., Mırçık, A. B., Kunduz, M., Çombaş, M., Öztürk, A., Deveci, E., & Kırpınar, İ. (2019). Effects of cognitive behavioral therapy, existential psychotherapy and supportive counselling on facial emotion recognition among patients with mild or moderate depression. Psychiatry Investigation, 16(7), 491.

Zeni, C. P., & Singh, M. K. (2018). Advances in Pediatric Bipolar Offspring Research. 65th Annual Meeting.

# APPENDIX IV

## Studies included only at a systematic review level

**Bio et al. (2013)** conducted a study comparing adults with BD and HCs in an identification FER task, assessing the accuracy of emotion recognition within these groups. The data presented in Table 1 indicate that individuals with BD demonstrated higher scores in recognizing facial emotions compared to HCs. However, this finding contradicts the information reported in the main text, results section, where it is suggested that FER scores were lower in the BD group. Given this inconsistency, we opted to exclude this paper from the meta-analysis and only include it in the systematic review.

**Brotman et al. (2008)** conducted a study comparing children/adolescents with BD, FDRs, and HCs in an identification FER task, measuring the number of morphs required before a response or correct emotion recognition within these groups. Both individuals with BD and FDRs required a higher emotional intensity before responding compared to HCs. Similarly, compared to HCs, both individuals with BD and FDRs required a higher emotional intensity before correctly identifying the emotion. As this study was the sole provider of this outcome measure, it was not included in the meta-analysis but was only included into the systematic review.

**David et al. (2014)** conducted a study comparing adults with BD and HCs in an identification FER task, assessing the accuracy of emotion recognition within these groups. Although raw data regarding the performance of these populations was not provided, the authors reported that both people with BD experiencing a manic episode or in euthymia performed worse than HCs in emotion recognition, while people with BD experiencing a depressive episode did not differ from HCs. Due to the unavailability of data for conversion into a format suitable for meta-analysis, this study was excluded from the meta-analysis but included solely in the systematic review.

**Fulford et al. (2014)** conducted a study comparing adults with BD and HCs in an identification FER task, assessing the accuracy of emotion recognition within these groups. Although raw data regarding the performance of these populations was not provided, the authors reported no significant differences in facial emotion-labeling accuracy between individuals with BD and HCs. Due to the unavailability of data for conversion into a format suitable for meta-analysis, this study was excluded from the meta-analysis but included solely in the systematic review.

**Furlong et al. (2022)** conducted a study comparing adults with BD and HCs in an identification FER task, examining the accuracy and reaction time of emotion recognition within these groups. During the FER task, participants were presented with a target word (e.g., "angry") at the beginning of each block and instructed to identify stimuli matching the target word (i.e., emotional faces) and stimuli not matching the target word (i.e., black and white photographs of houses or cars with perceptual similarities to faces). Individuals with BD exhibited significantly lower accuracy in recognizing fearful and neutral faces. Since this study employed a unique FER task that was not directly comparable to others, it was excluded from the meta-analysis but included solely in the systematic review.

**Kaufmann et al. (2017)** conducted a study comparing adults with BD and HCs in a discrimination FER task. Raw data regarding the performance of these populations was not provided. Due to the unavailability of data for conversion into a format suitable for meta-analysis, this study was excluded from the meta-analysis but included solely in the systematic review.

**Hassel et al. (2008)** conducted a study comparing adults with BD and HCs in an identification FER task, focusing on the accuracy of emotion recognition within these groups. Although raw data regarding the performance of these populations was not provided, the authors reported no significant differences in facial emotion-labeling accuracy between individuals with BD and HCs. Due to the unavailability of data for conversion into a format suitable for meta-analysis, this study was excluded from the meta-analysis but included solely in the systematic review.

**Murao-Miranda et al. (2012)** conducted a study comparing adults with BD and HCs in an identification FER task. The authors employed a Gaussian process classifier to discern the pattern of whole-brain activity for varying intensity facial expressions and reported the accuracies of the models for each population. However, due to the lack of data in a format suitable for meta-analysis, this study was excluded from the meta-analysis but retained solely in the systematic review.

**Lembke et al. (2002)** conducted a study comparing adults with BD and HCs in an identification FER task, with a focus on the accuracy of emotion recognition within these groups. While raw data concerning the performance of these populations was not available, the authors reported that individuals with BD exhibited lower abilities in emotion recognition compared to HCs. However, due to the absence of data suitable for conversion into a meta-analyzable format, this study was excluded from the meta-analysis but retained solely in the systematic review.

**Ruihua et al. (2021)** conducted a study comparing adults with BD and HCs in an identification FER task, focusing on the reaction time of emotion recognition within these groups. During the FER task, participants were instructed to identify a small set of emotions (e.g., happiness and sadness) assigned to specific keyboard keys (e.g., 1 or 2). Each block presented two different faces for a brief duration, and participants were required to press the button corresponding to the emotion they perceived. Individuals with BD demonstrated significantly longer reaction times in recognizing happy-sad and happy-angry faces. Because this study used a unique FER task not directly comparable to others, it was excluded from the meta-analysis but included solely in the systematic review.

**Schenkel et al. (2007)** conducted a study comparing children/adolescents with BD and HCs in an identification FER task, focusing on the accuracy of emotion recognition within these groups. During the FER task, participants were presented with faces expressing emotions at varying intensities and were tasked with labeling the degree of the emotion. Due to the unique nature of the FER task employed in this study, which is not directly comparable to others, it was excluded from the meta-analysis but included solely in the systematic review.

**Sinha et al. (2020)** conducted a study comparing adults with BD and HCs in an identification FER task. Raw data regarding the performance of these populations was not provided. Due to the unavailability of data for conversion into a format suitable for meta-analysis, this study was excluded from the meta-analysis but included solely in the systematic review.

**Wegbreit et al. (2015)** conducted a study comparing mixed age groups with BD and HCs in an identification FER task, focusing on the number of errors made during an emotion recognition task. Since this study was the only one providing this outcome measure for this age group, it was not incorporated into the meta-analysis but was exclusively included in the systematic review.

## Characteristics of the tasks adopted by the included studies

**eTable n. 3 - Detailed description of the emotion recognition task adopted by the included studies**

| **Study, year** | **Emotion recognition atlas and/or tool** | **Facial emotions showed** | **Neutral** | **Number of stimuli (duration /ISI)** | **Presence of practice** | **Number of posers (M/F ratio)** | **Emotion intensity (face morphing)** |
| --- | --- | --- | --- | --- | --- | --- | --- |
| ***Facial Emotion Discrimination*** | | | | | | | |
| Addington et al., 1998 | POFA/Facial affect matching task | Anger, disgust, fear, happiness, sadness, surprise | Yes | 42 | Yes (3) | 3 | 100% (no) |
| Bellack et al., 1996 | POFA, FOE | Anger, disgust, fear, happiness, sadness, surprise | No | 15 (15 sec) | No | NA (balanced) | 100% (no) |
| Benito et al., 2013 | POFA, FOE | Anger, disgust, happiness, sadness, shame, surprise | No | 30 | No | NA | 100% (no) |
| Bozikas et al., 2006 | KAMT | Anger, disgust, fear, happiness, sadness, surprise | No | 30 | No | 7 (3/4) | 100% (no) |
| Burger et al., 2017 | NimStim database | Anger, fear, happiness | No | 48 (2 sec/3 sec) | No | NA (balanced) | 100% (no) |
| Darker et al., 2021 | MIMI, FEED | Disgust, fear | No | 60 (2 sec) | No | 6 (3/3) | From 33% to 100% in five steps (yes) |
| Fernandes et al., 2016 | ER-40 | Happiness, sadness | Yes | 40 | No | NA (balanced) | 100% (no) |
| Foland-Ross et al., 2012 | Emotion matching task | NA | NA | 16 (4 sec) | Yes | NA | 100% (no) |
| Getz et al., 2003 | POFA | Anger, disgust, fear, happiness, sadness, surprise | No | 36 (0.75 sec) | No | 3 (1/2) | 100% (no) |
| Hulvershorn et al., 2012 | NimStim database, 3-minute emotion-matching task | Anger, fear | No | 15 | Yes | NA | 100% (no) |
| Hwang et al., 2021 | Emotion perception test | Pleasant, unpleasant | Yes | 108 | No | NA | 100% (no) |
| Kaufmann et al., 2017 | Face recognition task | Positive, negative | No | 24 (5 sec/0sec) | No | NA | 100% (no) |
| Lahera et al., 2015 | FEDT | Anger, disgust, happiness, sadness, shame, surprise | No | 30 (15 sec) | No | NA | 100% (no) |
| Li et al., 2022 | CAFPS | Anger, fear | No | 12 (5 sec) | No | NA | 100% (no) |
| Reddy et al., 2022 | TRENDS | Anger, disgust, fear, sadness | No | 32 (10 sec) | No | NA | 100% (no) |
| Rossell et al., 2014 | Comprehensive affective testing system | Anger, disgust, fear, happiness, sadness, surprise | Yes | 22 | No | 1 | 100% (no) |
| Schenkel et al., 2007 | University of Pennsylvania computerized neuropsychological tests | Happiness, sadness | Yes | 40 | Yes | NA | From 0% to 100% (no) |
| Ulusoy et al., 2020 | FEDT | Anger, fear, happiness, sadness, shame, surprise | No | 30 | No | NA | 100% (no) |
| Vaskinn et al., 2007 | POFA, FOE | Anger, disgust, happiness, sadness, shame, surprise | No | 30 | No | NA | 100% (no) |
| ***Facial Emotion Identification*** | | | | | | | |
| Addington et al., 1998 | POFA/Facial affect labelling task | Anger, disgust, fear, happiness, sadness, surprise | Yes | 21 (0.5 sec) | Yes (3) | 3 | 100% (no) |
| Almeida et al., 2010 | POFA/FEEST | Fear, happiness, sadness | Yes | 180 (2 sec/4.9 sec) | No | NA | 50%, 100% (no) |
| Altamura et al., 2016 | KDEF | Anger, happiness | Yes | 10 (4 sec/0.2 sec) | No | NA (balanced) | From 0% to 100% in 98 steps (yes) |
| Baez et al., 2013 | POFA/Emotional morphing | Anger, disgust, fear, happiness, sadness, surprise | Yes | 48 (0.5 sec) | No | NA | From 0% to 100% in twenty steps (yes) |
| Barbosa et al., 2023 | POFA/Facial expression recognition in Mini-SEA | Anger, disgust, fear, happiness, sadness, surprise | Yes | 35 | No | NA | 100% (no) |
| Bellack et al., 1996 | POFA, FOE | Anger, disgust, fear, happiness, sadness, surprise | No | 19 (15 sec/10 sec) | No | NA | 100% (no) |
| Benito et al., 2013 | POFA, FOE | Anger, disgust, happiness, sadness, shame, surprise | No | 19 | No | NA | 100% (no) |
| Benito et al., 2013 | ER-40 | Anger, fear, happiness, sadness | Yes | 40 | No | 40 (20/20) | 100% (no) |
| Bio et al., 2013 | POFA/EK | Anger, disgust, fear, happiness, sadness, surprise | No | 60 | Yes | 10 (4/6) | 100% (no) |
| Bjertrup et al., 2021 | POFA/FERT | Anger, disgust, fear, happiness, sadness, surprise | Yes | 250 (0.5 sec) | No | 10 | From 0% to 100% in ten steps (yes) |
| Bora et al., 2005 | Faces Test | Anger, disgust, distress, fear, happiness, sadness, surprise | No | 10 | No | 1 (0/1) | 100% (no) |
| Bozorg et al., 2014 | Cohn-Kanade/FERT | Anger, happiness, sadness | Yes | 100 (2.6 sec/1.5 sec) | Yes | 5 (3/2) | 100% (no) |
| Branco et al., 2018 | FERT | Anger, disgust, fear, happiness, sadness, surprise | Yes | 78 (0.2 sec, 0.5 sec, 1 sec) | No | 4 (2/2) | 100% (no) |
| Brotman et al., 2008 | DANVA | Anger, fear, happiness, sadness | No | 24 (2 sec) | No | NA | 100% (no) |
| Brotman et al., 2008 | POFA/EEMT | Anger, disgust, fear, happiness, sadness, surprise | Yes | NA | No | NA | From 0% to 100% in 39 steps (yes) |
| Darke et al., 2021 | MIMI, FEED | Disgust, fear | No | 60 (2 sec) | No | 6 (3/3) | From 33% to 100% in five steps (yes) |
| Daros et al., 2014 | PEAT | Happiness, sadness | Yes | 40 | Yes (5) | NA (balanced) | 100% (no) |
| David et al., 2014 | POFA | Anger, disgust, fear, happiness, sadness, surprise | 0 | 150 (5 sec) | Yes | NA | 50%, 70%, 90% (no) |
| de Siqueria Rotenberg et al., 2023 | P1vital Affective Faces | Anger, disgust, fear, happiness, sadness, surprise | Yes | 250 (0.5 sec) | No | NA | From 0% to 100% in ten steps (yes) |
| Derntl et al., 2009 | VERT-K | Anger, disgust, fear, happiness, sadness | Yes | 36 | No | 36 (18/18) | 100% (no) |
| Derntl et al., 2012 | 3D Facial Expression Task | Anger, disgust, fear, happiness, sadness | Yes | 30 (4 sec) | No | NA | 100% (no) |
| Dima et al., 2016 | POFA | Anger, fear, sadness | Yes | 60 (2 sec/ 5 sec) | No | 10 | 150% (no) |
| Fernandes et al., 2016 | ER-40 | Anger, fear, happiness, sadness | Yes | 40 | No | 8 (4/4) | 100% (no) |
| Foland-Ross et al., 2012 | Emotion labeling task | NA | NA | 16 (4 sec) | Yes | NA | 100% (no) |
| Fulford et al., 2014 | FEEST | Anger, fear, happiness, sadness | Yes | 44 (0.3 sec) | Yes | 4 | 25%, 50%, 75% (no) |
| Furlong et al., 2022 | POFA | Anger, fear, sadness | Yes | 96 (0.5 sec/4 sec) | Yes (1) | NA | 60% (no) |
| Getz et al., 2003 | POFA | Anger, disgust, fear, happiness, sadness, surprise | No | 18 (0.75 sec) | No | 3 (1/2) | 100% (no) |
| Goghari et al., 2013 | Pennsylvania emotive faces | Anger, fear, happiness, sadness | Yes | 60 | No | 60 (30/30) | 100% (no) |
| Golkhatmi et al., 2015 | POFA | Anger, disgust, fear, happiness, sadness, surprise | No | 36 | No | 6 (3/3) | 100% (no) |
| Gray et al., 2024 | POFA | Anger, disgust, fear, happiness, sadness, surprise | Yes | 6 (0.5 sec) | Yes | NA | From 0% to 100% in ten steps (yes) |
| Guyer et al., 2007 | DANVA | Anger, fear, happiness, sadness | No | 24 (2 sec) | No | 24 (12/12) | Low and high intensity (no) |
| Harmer et al., 2002 | POFA | Anger, disgust, fear, happiness, sadness, surprise | Yes | 250 (0.5 sec) | Yes | 10 | From 0% to 100% in ten steps (yes) |
| Hassel et al., 2008 | KDEF | Anger, disgust, fear, happiness, sadness | Yes | 45 | No | NA | 100% (no) |
| Hoertangl et al., 2011 | JACFEE, FEEL | Anger, disgust, fear, happiness, sadness, surprise | Yes | 42 (0.3 sec) | Yes (6) | NA | 100% (no) |
| Iakimova et al., 2016 | Radboud Faces database | Anger, disgust, fear, happiness, sadness, surprise | Yes | 42 (NA/1.5 sec) | No | 6 (3/3) | 100% (no) |
| Jogia et al., 2011 | POFA | Fear | Yes | 60 (2 sec/5 sec) | No | 10 (4/6) | 150% (no) |
| Khafif et al., 2023 | ER-40 | Anger, fear, happiness, sadness | Yes | 40 | No | 40 (20/20) | 100% (no) |
| Kim et al., 2013 | POFA | Anger, fear, happiness, sadness | Yes | 130 (2 sec/1.3 sec) | Yes (10) | 10 (6/4) | From 60% to 100% in three steps (yes) |
| Kjaerstad et al., 2022 | P1vital Affective Faces | Anger, disgust, fear, happiness, sadness, surprise | Yes | 250 (0.5 sec) | No | NA | From 0% to 100% in ten steps (yes) |
| Lahera et al., 2015 | FEIT | Anger, disgust, happiness, sadness, shame, surprise | No | 19 (15 sec) | No | NA | 100% (no) |
| Lahera et al., 2015 | ER-40 | Anger, fear, happiness, sadness | Yes | 40 | No | 40 (20/20) | 100% (no) |
| Lawlor-Savage et al., 2014 | 3D Facial Expression Task | Anger, fear, happiness, sadness | Yes | 240 (3 sec) | No | NA | 100% (no) |
| Lee et al., 2020 | ER-96 | Anger, fear, sadness, happiness | Yes | 30 | No | NA | 100% (no) |
| Lelli-Chiesa et al., 2011 | POFA | Sadness | Yes | 40 (2 sec/5 sec) | No | NA | 150% (no) |
| Lembke et al., 2002 | POFA | Anger, disgust, fear, happiness, sadness, surprise | No | 33 | No | NA | 100% (no) |
| Lescalier et al., 2015 | KDEF | Happiness, sadness | Yes | 126 (2 sec/0.5 sec) | No | 42 (21/21) | 100% (no) |
| MacPherson et al., 2021 | DANVA-2 | Anger, fear, happiness, sadness | No | 24 (2 sec) | No | 24 (12/12) | 100% (no) |
| Maila de Castro et al., 2015 | ER-40 | Anger, fear, happiness, sadness | Yes | 40 | No | 40 (20/20) | 100% (no) |
| Malhi et al., 2007 | POFA | Disgust, fear | Yes | 210 (2.75 sec/0.25 sec) | No | NA | 100% (no) |
| Martino et al., 2011 | POFA, EK | Anger, disgust, fear, happiness, sadness, surprise | No | 60 (5 sec) | No | NA | 100% (no) |
| McClure et al., 2005 | DANVA | Anger, fear, happiness, sadness | No | 24 (2 sec) | No | 24 (12/12) | 100% (no) |
| Millet et al., 2023 | CANTAB | Anger, disgust, fear, happiness, sadness, surprise | No | 180 (0.2 sec) | No | NA | 100% (no) |
| Miola et al., 2023 | NimStim database | Anger, disgust, sadness | Yes | 140 | No | NA | 100% (no) |
| Mourao-Miranda et al., 2012 | FEEST | Happiness | Yes | 60 (2 sec/4.9 sec) | No | NA | 50%, 100% (no) |
| Navarra-Ventura et al., 2021 | POFA | Anger, disgust, fear, happiness, sadness, surprise | No | 60 (5 sec) | No | NA | 100% (no) |
| Nigam et al., 2021 | TRENDS | Anger, disgust, fear, happiness, sadness, surprise | Yes | 52 | No | 4 (2/2) | 33%, and100% (no) |
| Pan et al., 2013 | DANVA-2 | Anger, fear, happiness, sadness | No | 24 (5 sec) | No | 24 | 100% (no) |
| Pavuluri et al., 2009 | Gur Faces | Anger, happiness | No | 48 (5 sec) | No | NA (balanced) | 100% (no) |
| Priyesh et al., 2022 | TRENDS | Anger, happiness | Yes | 192 (0.3 sec/0.6 sec) | Yes | NA | 100% (no) |
| Quidè et al., 2018 | TASIT | Anger, disgust, fear, happiness, sadness, surprise | No | 28 (37.5 sec) | Yes | 1 | 100% (no) |
| Quidè et al., 2018 | POFA | Anger, disgust, fear, happiness, sadness, surprise | No | 60 | No | NA | 100% (no) |
| Reddy et al., 2022 | TRENDS | Anger, disgust, fear, sadness | No | 32 (10 sec) | No | NA | 100% (no) |
| Robinson et al., 2015a | POFA | Anger, disgust, fear, happiness, sadness | Yes | 84 (1 sec) | Yes | 4 (2/2) | 20%, 40%, 60%, and 80% (no) |
| Robinson et al., 2015b | POFA | Anger, disgust, fear, happiness, sadness | Yes | 80 (1 sec) | Yes | 4 (2/2) | From 0% to 100% in twenty steps (yes) |
| Rossell et al., 2014 | Comprehensive Affective Testing System | Anger, disgust, fear, happiness, sadness, surprise | Yes | 16 | No | NA | 100% (no) |
| Rowland et al., 2013 | POFA | Anger, disgust, fear, happiness, sadness, surprise | No | 60 | No | NA | 100% (no) |
| Rowland et al., 2013 | TASIT | Anger, disgust, fear, happiness, sadness, surprise | No | 28 | No | 1 | 100% (no) |
| Rubin et al., 2022 | Cohn-Kanade | Anger, disgust, fear, happiness, sadness, surprise | Yes | 36 (8 sec) | Yes | NA | From 0% slowly transitioning into 100% (no) |
| Ruihua et al., 2021 | POFA | Anger, disgust, fear, happiness, sadness, surprise | Yes | 60 (0.2 sec/0.2 sec) | No | 10 (6/4) | 100% (no) |
| Ruocco et al., 2014 | ER-40 | Anger, fear, happiness, sadness | Yes | 40 | No | NA | 100% (no) |
| Ryan et al., 2013 | POFA | Anger, disgust, fear, happiness, sadness, surprise | No | 36 (1.5 sec/0.2 sec) | No | NA | 100% (no) |
| Sagar et al., 2013 | FEEST | Anger, disgust, fear, happiness, sadness, surprise | No | 60 (5 sec) | No | NA | 100% (no) |
| Schaefer et al., 2010 | POFA | Anger, disgust, fear, happiness, sadness, surprise | Yes | 36 (0.1 sec) | No | 6 (3/3) | From 0% to 100% in thirty-nine steps (yes) |
| Schenkel et al., 2012 | DANVA | Anger, happiness | Yes | 40 | No | NA | 33%, 66%, and 100% (no) |
| Schenkel et al., 2007 | University of Pennsylvania computerized neuropsychological tests | Happiness, sadness | Yes | 40 | Yes | NA | 33%, 66%, and 100% (no) |
| Schenkel et al., 2020 | DANVA-2 | Anger, fear, happiness, sadness | No | 48 (0.2 sec) | No | NA | 100% (no) |
| Seidel et al., 2012 | Gur Faces | Anger, disgust, fear, happiness, sadness | Yes | 60 (5 sec) | No | NA | 100% (no) |
| Seymour et al., 2013 | DANVA | Anger, fear, happiness, sadness | No | 48 (2 sec) | No | 48 (24/24) | 50%, 100% (no) |
| Shah et al., 2009 | POFA | Fear, happiness | Yes | 30 (2 sec/8 sec) | No | 10 (5/5) | 100% (no) |
| Sinha et al., 2020 | NA | Anger, fear, happiness, sadness | No | 20 (5 sec/2 sec) | No | NA | 100% (no) |
| Soeiro-de-Souza et al., 2012 | POFA | Anger, disgust, fear, happiness, sadness, surprise | No | 60 | No | 10 (6/4) | 100% (no) |
| Soeiro-de-Souza et al., 2012 | POFA | Anger, disgust, fear, happiness, sadness, surprise | No | 60 | No | 10 (6/4) | 100% (no) |
| Summers et al., 2006 | POFA | Anger, disgust, happiness, sadness, shame, surprise | Yes | 18 | No | NA | From 0% to 100% in twenty steps (yes) |
| Tesli et al., 2015 | POFA | Anger, fear, happiness | No | 48 (0.9 sec/0 sec) | No | NA | 100% (no) |
| Thaler et al., 2013 | BLERT | Anger, disgust, fear, happiness, sadness, surprise | Yes | 21 (10 sec) | No | 1 | 100% (no) |
| Ulusoy et al., 2020 | FEIT | Anger, fear, happiness, sadness, shame, surprise | No | 19 (15 sec/10 sec) | No | NA | 100% (no) |
| Van Rheenen et al., 2014 | POFA | Anger, fear, happiness, sadness | No | 40 (1.5 sec/1.5 sec) | No | 10 (5/5) | From 25% to 100% in four steps (yes) |
| Van Rheenen et al., 2014 | POFA | Anger, fear, happiness, sadness | Yes | 130 (2 sec/1.5 sec) | Yes | 10 (5/5) | 33%, 67%, 100% (no) |
| Van Rheenen et al., 2017 | POFA | Anger, fear, happiness, sadness | Yes | 50 (2 sec/1.5 sec) | Yes | 10 (5/5) | 100% (no) |
| Vaskinn et al., 2007 | POFA, FOE | Anger, disgust, happiness, sadness, shame, surprise | No | 19 | No | NA | 100% (no) |
| Vederman et al., 2012 | NA | Anger, fear, happiness, sadness | Yes | 52 (0.3 sec/2.7 sec) | No | NA | 100% (no) |
| Venn et al., 2004 | POFA | Anger, disgust, fear, happiness, sadness, surprise | No | 36 | Yes | NA | From 0% to 150% in eighteen steps (yes) |
| Versace et al., 2010 | POFA | Happiness, sadness | Yes | 120 | No | NA | 50%, 100% (no) |
| Vierck et al., 2015 | POFA | Anger, disgust, fear, happiness, sadness | Yes | 110 (0.5 sec) | No | NA | From 10% to 90% in five steps (yes) |
| Wegbreit et al., 2015 | DANVA-2 | Anger, fear, happiness, sadness | No | 24 (2 sec) | No | 48 (24/24) | 50%, 100% (no) |
| Wiggins et al., 2017 | POFA | Anger, fear, happiness | Yes | 420 (4 sec) | No | NA | From 0% to 100% in four steps (yes) |
| Wynn et al., 2013 | POFA | Anger, fear, happiness, sadness, shame, surprise | No | 108 (0.5 sec, 1 sec) | No | 36 (18/18) | 100% (no) |
| Yalcin-Siedentopf et al., 2014 | FEEL | Anger, disgust, fear, happiness, sadness, surprise | No | 42 (0.3 sec) | No | NA | 100% (no) |
| Zhang et al., 2018 | NA | Anger, happiness, sadness | Yes | NA (0.5 sec, 1.5 sec) | No | NA | 100% (no) |

# APPENDIX V

## Quality according to the Newcastle-Ottawa Scale of included cross-sectional studies

**eTable n. 4 - Detailed description of the quality of the included cross-sectional studies according to the Newcastle-Ottawa Scale**

| **Author, year** | **Representativeness of the sample (SELECTION)** | **Sample Size (SELECTION)** | **Non-respondents (SELECTION)** | **Ascerteinment of the exposure (SELECTION)** | **Comparability (COMPARABILITY)** | **Assessment of the outcome (OUTCOME)** | **Statistical Test (OUTCOME)** | **TOTAL** | **AHRQ Standards** |
| --- | --- | --- | --- | --- | --- | --- | --- | --- | --- |
| Almeida et al., 2010 | 0 | 0 | 1 | 2 | 2 | 1 | 1 | 7 | GOOD |
| Altamura et al., 2016 | 0 | 0 | 1 | 2 | 1 | 1 | 1 | 6 | GOOD |
| Baez et al., 2013 | 0 | 0 | 1 | 1 | 2 | 1 | 1 | 6 | FAIR |
| Barbosa et al., 2023 | 0 | 0 | 1 | 2 | 2 | 1 | 1 | 7 | GOOD |
| Bellack et al., 1996 | 0 | 0 | 1 | 2 | 1 | 1 | 1 | 6 | GOOD |
| Benito et al., 2013 | 0 | 0 | 1 | 1 | 2 | 1 | 1 | 6 | FAIR |
| Bio et al., 2013 | 0 | 0 | 0 | 2 | 1 | 1 | 1 | 5 | FAIR |
| Bora et al., 2005 | 0 | 0 | 1 | 2 | 2 | 1 | 1 | 7 | GOOD |
| Bozikas et al., 2006 | 0 | 0 | 1 | 2 | 2 | 1 | 1 | 7 | GOOD |
| Bozorg et al., 2014 | 1 | 0 | 1 | 2 | 2 | 1 | 1 | 8 | GOOD |
| Branco et al., 2017 | 0 | 1 | 1 | 2 | 1 | 1 | 1 | 7 | GOOD |
| Brotman et al., 2008 | 0 | 0 | 1 | 2 | 1 | 1 | 1 | 6 | GOOD |
| Brotman et al., 2008 | 0 | 0 | 1 | 2 | 1 | 1 | 1 | 6 | GOOD |
| Burger et al., 2017 | 0 | 0 | 1 | 2 | 2 | 1 | 1 | 7 | GOOD |
| Darke et al., 2021 | 0 | 0 | 1 | 1 | 0 | 1 | 1 | 4 | POOR |
| David et al., 2014 | 0 | 0 | 1 | 2 | 1 | 1 | 1 | 6 | GOOD |
| Derntl et al., 2009 | 0 | 0 | 1 | 2 | 2 | 1 | 1 | 7 | GOOD |
| Derntl et al., 2012 | 0 | 0 | 1 | 2 | 2 | 1 | 1 | 7 | GOOD |
| Dima et al., 2016 | 0 | 0 | 1 | 1 | 2 | 1 | 1 | 6 | GOOD |
| Fernandes et al., 2016 | 0 | 0 | 1 | 2 | 2 | 1 | 1 | 7 | GOOD |
| Foland-Ross et al., 2012 | 0 | 0 | 1 | 2 | 2 | 1 | 1 | 7 | GOOD |
| Fulford et al., 2014 | 0 | 0 | 0 | 2 | 2 | 1 | 1 | 6 | FAIR |
| Furlong et al., 2022 | 0 | 0 | 1 | 2 | 2 | 1 | 1 | 7 | GOOD |
| Getz et al., 2003 | 0 | 0 | 1 | 2 | 2 | 1 | 1 | 7 | GOOD |
| Goghari et al., 2013 | 0 | 0 | 1 | 2 | 2 | 1 | 1 | 7 | GOOD |
| Golkhatmi et al., 2013 | 0 | 0 | 1 | 1 | 0 | 1 | 1 | 4 | POOR |
| Gray et al., 2024 | 0 | 0 | 1 | 2 | 0 | 1 | 1 | 5 | POOR |
| Guyer et al., 2007 | 0 | 0 | 1 | 2 | 2 | 1 | 1 | 7 | GOOD |
| Harmer et al., 2002 | 0 | 0 | 1 | 2 | 2 | 1 | 1 | 7 | GOOD |
| Hassel et al., 2008 | 0 | 0 | 0 | 2 | 1 | 1 | 1 | 5 | FAIR |
| Hoertnagl et al., 2011 | 0 | 0 | 1 | 2 | 2 | 1 | 1 | 7 | GOOD |
| Hulvershorn et al., 2012 | 1 | 0 | 1 | 2 | 2 | 1 | 1 | 8 | GOOD |
| Hwang et al., 2021 | 1 | 0 | 1 | 1 | 2 | 1 | 1 | 7 | GOOD |
| Iakimova et al., 2016 | 0 | 0 | 1 | 1 | 2 | 1 | 1 | 6 | FAIR |
| Jogia et al., 2011 | 0 | 0 | 1 | 2 | 2 | 1 | 1 | 7 | GOOD |
| Kaufmann et al., 2017 | 0 | 0 | 1 | 2 | 1 | 1 | 1 | 6 | GOOD |
| Khafif et al., 2023 | 0 | 0 | 1 | 2 | 0 | 1 | 1 | 5 | POOR |
| Kim et al., 2013 | 0 | 0 | 1 | 2 | 1 | 1 | 1 | 6 | GOOD |
| Lahera et al., 2015 | 0 | 0 | 1 | 1 | 2 | 1 | 1 | 6 | FAIR |
| Lawlor-Savage et al., 2014 | 0 | 0 | 1 | 1 | 1 | 1 | 1 | 5 | FAIR |
| Lee et al., 2020 | 0 | 0 | 0 | 2 | 1 | 1 | 1 | 5 | FAIR |
| Lelli-Chiesa et al., 2010 | 0 | 0 | 0 | 2 | 2 | 1 | 1 | 6 | FAIR |
| Lembke et al., 2002 | 0 | 0 | 0 | 2 | 0 | 1 | 1 | 4 | POOR |
| Lescalier et al., 2015 | 0 | 0 | 1 | 2 | 2 | 1 | 1 | 7 | GOOD |
| Li et al., 2022 | 0 | 0 | 1 | 2 | 2 | 1 | 1 | 7 | GOOD |
| Maila de Castro et al., 2015 | 0 | 0 | 1 | 2 | 2 | 1 | 1 | 7 | GOOD |
| Malhi et al., 2007 | 0 | 0 | 1 | 2 | 2 | 1 | 1 | 7 | GOOD |
| Martino et al., 2011 | 1 | 0 | 1 | 2 | 2 | 1 | 1 | 8 | GOOD |
| McClure et al., 2005 | 0 | 0 | 1 | 2 | 2 | 1 | 1 | 7 | GOOD |
| Millet et al., 2023 | 0 | 0 | 1 | 2 | 1 | 1 | 1 | 6 | GOOD |
| Miola et al., 2023 | 0 | 0 | 1 | 2 | 2 | 1 | 1 | 7 | GOOD |
| Murao-Miranda et al., 2012 | 0 | 0 | 0 | 2 | 2 | 1 | 1 | 6 | FAIR |
| Navarra-Ventura et al., 2021 | 1 | 0 | 1 | 1 | 2 | 1 | 1 | 7 | GOOD |
| Nigam et al., 2021 | 0 | 1 | 0 | 1 | 2 | 1 | 1 | 6 | FAIR |
| Pan et al., 2013 | 0 | 0 | 1 | 2 | 2 | 1 | 1 | 7 | GOOD |
| Pavuluri et al., 2009 | 0 | 0 | 0 | 2 | 2 | 1 | 1 | 6 | FAIR |
| Priyesh et al., 2022 | 0 | 1 | 1 | 1 | 1 | 1 | 1 | 6 | GOOD |
| Quide et al., 2018 | 1 | 0 | 1 | 2 | 0 | 1 | 1 | 6 | POOR |
| Reddy et al., 2022 | 0 | 0 | 1 | 2 | 2 | 1 | 1 | 7 | GOOD |
| Robinson et al., 2015 | 0 | 0 | 1 | 2 | 2 | 1 | 1 | 7 | GOOD |
| Rossell et al., 2014 | 0 | 0 | 1 | 2 | 0 | 1 | 1 | 5 | POOR |
| Rowland et al., 2012 | 1 | 0 | 1 | 0 | 0 | 1 | 1 | 4 | POOR |
| Rubin et al., 2022 | 1 | 0 | 1 | 2 | 0 | 1 | 1 | 6 | POOR |
| Ruihua et al., 2021 | 0 | 0 | 0 | 1 | 2 | 1 | 1 | 5 | FAIR |
| Ruocco et al., 2014 | 1 | 0 | 1 | 2 | 0 | 1 | 1 | 6 | POOR |
| Ryan et al., 2013 | 0 | 0 | 1 | 2 | 1 | 1 | 1 | 6 | GOOD |
| Sagar et al., 2013 | 0 | 0 | 1 | 2 | 0 | 1 | 1 | 5 | POOR |
| Schaefer et al., 2010 | 0 | 0 | 1 | 2 | 1 | 1 | 1 | 6 | GOOD |
| Schenkel et al., 2007 | 0 | 0 | 0 | 2 | 2 | 1 | 1 | 6 | FAIR |
| Schenkel et al., 2012 | 0 | 0 | 1 | 2 | 2 | 1 | 1 | 7 | GOOD |
| Schenkel et al., 2020 | 0 | 0 | 1 | 2 | 2 | 1 | 1 | 7 | GOOD |
| Seidel et al., 2012 | 0 | 0 | 1 | 2 | 1 | 1 | 1 | 6 | GOOD |
| Seymour et al., 2013 | 0 | 0 | 1 | 2 | 1 | 1 | 1 | 6 | GOOD |
| Shah et al., 2009 | 0 | 0 | 1 | 2 | 1 | 1 | 1 | 6 | GOOD |
| Sinha et al., 2020 | 0 | 0 | 1 | 2 | 2 | 1 | 1 | 7 | GOOD |
| Soeiro-de-Souza et al., 2012 | 0 | 0 | 1 | 2 | 2 | 1 | 1 | 7 | GOOD |
| Soeiro-de-Souza et al., 2012 | 0 | 0 | 1 | 2 | 1 | 1 | 1 | 6 | GOOD |
| Summers et al., 2006 | 0 | 0 | 1 | 2 | 1 | 1 | 1 | 6 | GOOD |
| Tesli et al., 2015 | 1 | 0 | 1 | 2 | 0 | 1 | 1 | 6 | POOR |
| Thaler et al., 2013 | 0 | 0 | 1 | 2 | 1 | 1 | 1 | 6 | GOOD |
| Ulusoy et al., 2020 | 0 | 1 | 1 | 2 | 2 | 1 | 1 | 8 | GOOD |
| Van Rheenen et al., 2014 | 0 | 0 | 1 | 2 | 2 | 1 | 1 | 7 | GOOD |
| Van Rheenen et al., 2017 | 0 | 0 | 1 | 1 | 0 | 1 | 1 | 4 | POOR |
| Vaskinn et al., 2007 | 0 | 0 | 1 | 2 | 1 | 1 | 1 | 6 | GOOD |
| Vederman et al., 2012 | 0 | 0 | 1 | 2 | 2 | 1 | 1 | 7 | GOOD |
| Venn et al., 2004 | 0 | 0 | 1 | 2 | 2 | 1 | 1 | 7 | GOOD |
| Versace et al., 2010 | 1 | 0 | 1 | 2 | 0 | 1 | 1 | 6 | POOR |
| Vierck et al., 2015 | 0 | 0 | 1 | 2 | 1 | 1 | 1 | 6 | GOOD |
| Wegbreit et al., 2015 | 0 | 0 | 0 | 2 | 1 | 1 | 1 | 5 | FAIR |
| Wiggins et al., 2017 | 0 | 0 | 1 | 2 | 2 | 1 | 1 | 7 | GOOD |
| Wynn et al., 2013 | 1 | 0 | 1 | 2 | 1 | 1 | 1 | 7 | GOOD |
| Yalcin-Siedentopf et al., 2014 | 0 | 0 | 0 | 2 | 2 | 1 | 1 | 6 | FAIR |
| Zhang et al., 2018 | 0 | 0 | 0 | 1 | 2 | 1 | 1 | 5 | FAIR |

## Quality according to the Newcastle-Ottawa Scale of included longitudinal studies

**eTable n. 5 - Detailed description of the quality of the included longitudinal studies according to the Newcastle-Ottawa Scale**

| **Author, year** | **Representativeness of the exposed cohort (SELECTION)** | **Representativeness of the non-exposed cohort (SELECTION)** | **Ascerteinment of the exposure (SELECTION)** | **Outcome of interest was not present at the beginning (SELECTION)** | **Comparability (COMPARABILITY)** | **Assessment of the outcome (OUTCOME)** | **Follow-up long enough (OUTCOME)** | **Adequacy of follow-up (OUTCOME)** | **TOTAL** | **AHRQ Standards** |
| --- | --- | --- | --- | --- | --- | --- | --- | --- | --- | --- |
| Addington et al., 1998 | 1 | 0 | 1 | 1 | 0 | 1 | 1 | 1 | 6 | POOR |
| Bjertrup et al., 2020 | 1 | 1 | 1 | 1 | 1 | 1 | 1 | 1 | 8 | GOOD |
| Daros et al., 2014 | 1 | 0 | 1 | 1 | 1 | 1 | 1 | 0 | 6 | FAIR |
| de Siqueria Rotenberg et al., 2023 | 1 | 0 | 1 | 1 | 2 | 1 | 1 | 1 | 8 | GOOD |
| Kjaerstad et al., 2022 | 1 | 0 | 1 | 1 | 2 | 1 | 1 | 1 | 8 | GOOD |
| MacPherson et al., 2021 | 0 | 1 | 1 | 0 | 2 | 1 | 1 | 0 | 6 | FAIR |

# APPENDIX VI

## Main analysis

The forest plots for each meta-analysis are available at https://osf.io/gax8e/?view_only=e076729b5c01413c82ff546a22ec7ce5

## Meta-regression analyses

**eTable n. 6 - Meta-regression analyses**

Predictors are organized following this order: i) *characteristics of the original study*; ii) *sociodemographic characteristics of people with BD*; iii) *clinical characteristics of people with BD*; iv) *FER task characteristics*.

The results are highlighted in green when a higher value of that specific predictor is significantly associated with a larger magnitude difference between the two groups.

The results are highlighted in blue when a higher value of that specific predictor is significantly associated with a smaller magnitude difference between the two groups.

The results are highlighted in yellow when a higher value of that specific predictor is significantly associated with a difference in the opposite direction compared to the lower value of the same predictor.

| **Control group** | **Emotion type** | **Outcome type** | **Predictor** | **Studies, n** | **Beta** | **95% CIs** | **p-value** | **SMD at predictor lower value** | **SMD at predictor higher value** |
| --- | --- | --- | --- | --- | --- | --- | --- | --- | --- |
| ***Identification*** | | | | | | | | | |
| *Adults* | | | | | | | | | |
| Level I | | | | | | | | | |
| HCs | Any emotion | Accuracy | Publication year | 65 | -0.016 | -0.032, 0.001 | 0.06 | -0.178 at 1996 | -0.607 at 2023 |
| HCs | Any emotion | Accuracy | Primary outcome (reference category, no) | 65 | -0.057 | -0.235, 0.121 | 0.53 | -0.438 at 0 | -0.495 at 1 |
| HCs | Any emotion | Accuracy | Age | 65 | -0.001 | -0.015, 0.014 | 0.92 | -0.454 at 20.03 | -0.48 at 57.4 |
| HCs | Any emotion | Accuracy | Education, years | 40 | 0.042 | -0.012, 0.096 | 0.13 | -0.903 at 3.46 | -0.306 at 17.7 |
| HCs | Any emotion | Accuracy | % of females | 63 | -0.052 | -0.694, 0.59 | 0.88 | -0.443 at 0.18 | -0.485 at 100 |
| HCs | Any emotion | Accuracy | Duration of illness | 28 | -0.005 | -0.038, 0.028 | 0.77 | -0.547 at 5.1 | -0.677 at 32.1 |
| HCs | Any emotion | Accuracy | Age at onset | 26 | -0.012 | -0.031, 0.006 | 0.19 | -0.367 at 16.53 | -0.651 at 39.62 |
| HCs | Any emotion | Accuracy | % of people with BD-I | 46 | -0.139 | -0.581, 0.303 | 0.54 | -0.352 at 0 | -0.491 at 100 |
| HCs | Any emotion | Accuracy | % of euthymic | 51 | -0.032 | -0.324, 0.261 | 0.83 | -0.482 at 0 | -0.514 at 100 |
| HCs | Any emotion | Accuracy | % of depressed | 48 | 0.241 | -0.158, 0.641 | 0.24 | -0.54 at 0 | -0.298 at 100 |
| HCs | Any emotion | Accuracy | % of (hypo)manic | 47 | -0.296 | -0.808, 0.216 | 0.26 | -0.491 at 0 | -0.787 at 100 |
| HCs | Any emotion | Accuracy | Symptoms severity scale, depression | 40 | 0.008 | -0.012, 0.027 | 0.44 | -0.484 at 1.72 | -0.297 at 26 |
| HCs | Any emotion | Accuracy | Symptoms severity scale, mania | 42 | -0.024 | -0.046, -0.003 | 0.028 | -0.354 at 0.67 | -1.023 at 28.1 |
| HCs | Any emotion | Accuracy | Morphed (reference category, no) | 65 | 0.111 | -0.129, 0.351 | 0.36 | -0.485 at 0 | -0.373 at 1 |
| HCs | Any emotion | Accuracy | Number of stimuli | 64 | 0.001 | -0.001, 0.002 | 0.24 | -0.523 at 10 | -0.299 at 250 |
| HCs | Any emotion | Accuracy | Number of emotions | 64 | -0.025 | -0.084, 0.034 | 0.4 | -0.375 at 1 | -0.527 at 7 |
| HCs | Any emotion | Accuracy | Duration of stimulus (msec) | 43 | -0.00004 | -6e-05, -1e-05 | 0.002 | -0.316 at 100 | -0.902 at 15000 |
| HCs | Any emotion | Accuracy | ISI, msec | 18 | -0.00002 | -7e-05, 2e-05 | 0.31 | -0.304 at 0 | -0.529 at 10000 |
| HCs | Any emotion | Accuracy | Practice (reference category, no) | 65 | 0.254 | 0.033, 0.476 | 0.025 | -0.512 at 0 | -0.258 at 1 |
| HCs | Any emotion | Accuracy | Minimum emotion intensity | 65 | -0.002 | -0.004, 0.001 | 0.13 | -0.325 at 1 | -0.581 at 150 |
| HCs | Any emotion | Accuracy | Maximum emotion intensity | 65 | 0.001 | -0.006, 0.009 | 0.71 | -0.498 at 80 | -0.402 at 150 |
| HCs | Any emotion | Accuracy | Presence of neutral faces (reference category, no) | 63 | 0.152 | -0.024, 0.327 | 0.09 | -0.55 at 0 | -0.398 at 1 |
| HCs | Any emotion | Reaction time | Publication year | 25 | -0.053 | -0.102, -0.003 | 0.039 | 1.02 at 2007 | 0.178 at 2023 |
| HCs | Any emotion | Reaction time | Primary outcome (reference category, no) | 25 | 0.008 | -0.487, 0.504 | 0.97 | 0.57 at 0 | 0.578 at 1 |
| HCs | Any emotion | Reaction time | Age | 25 | 0.051 | 0.016, 0.086 | 0.004 | -0.35 at 20.03 | 0.991 at 46.2 |
| HCs | Any emotion | Reaction time | Education, years | 15 | -0.017 | -0.155, 0.12 | 0.81 | 0.881 at 3.46 | 0.635 at 17.7 |
| HCs | Any emotion | Reaction time | % of females | 25 | 0.408 | -1.067, 1.882 | 0.59 | 0.433 at 0.19 | 0.763 at 100 |
| HCs | Any emotion | Reaction time | Duration of illness | 13 | 0.014 | -0.045, 0.073 | 0.64 | 0.429 at 5.1 | 0.658 at 21.2 |
| HCs | Any emotion | Reaction time | Age at onset | 10 | 0.137 | 0.096, 0.177 | <0.001 | -0.104 at 16.9 | 2.999 at 39.62 |
| HCs | Any emotion | Reaction time | % of people with BD-I | 20 | -0.249 | -1.299, 0.801 | 0.64 | 0.87 at 0 | 0.621 at 100 |
| HCs | Any emotion | Reaction time | % of euthymic | 20 | 0.006 | -0.557, 0.568 | 0.98 | 0.545 at 0 | 0.551 at 100 |
| HCs | Any emotion | Reaction time | % of depressed | 19 | -0.25 | -1.106, 0.607 | 0.57 | 0.566 at 0 | 0.379 at 75 |
| HCs | Any emotion | Reaction time | Symptoms severity scale, depression | 16 | 0.001 | -0.088, 0.091 | 0.98 | 0.576 at 2 | 0.588 at 12 |
| HCs | Any emotion | Reaction time | Symptoms severity scale, mania | 17 | -0.042 | -0.139, 0.054 | 0.39 | 0.656 at 0.9 | 0.283 at 9.7 |
| HCs | Any emotion | Reaction time | Morphed (reference category, no) | 25 | 0.257 | -0.355, 0.869 | 0.41 | 0.523 at 0 | 0.78 at 1 |
| HCs | Any emotion | Reaction time | Number of stimuli | 24 | -0.002 | -0.005, 0.002 | 0.29 | 0.715 at 10 | 0.264 at 250 |
| HCs | Any emotion | Reaction time | Number of emotions | 24 | -0.048 | -0.201, 0.105 | 0.54 | 0.722 at 1 | 0.484 at 6 |
| HCs | Any emotion | Reaction time | Duration of stimulus (msec) | 19 | 0.00002 | -0.00011, 0.00015 | 0.73 | 0.61 at 300 | 0.831 at 10000 |
| HCs | Any emotion | Reaction time | ISI, msec | 11 | 0.00001 | -0.00011, 0.00014 | 0.84 | 0.606 at 0 | 0.705 at 8000 |
| HCs | Any emotion | Reaction time | Practice (reference category, no) | 25 | -0.382 | -0.896, 0.132 | 0.14 | 0.678 at 0 | 0.296 at 1 |
| HCs | Any emotion | Reaction time | Minimum emotion intensity | 25 | -0.00006 | -0.005, 0.005 | 0.98 | 0.578 at 1 | 0.57 at 150 |
| HCs | Any emotion | Reaction time | Maximum emotion intensity | 25 | 0.008 | -0.006, 0.021 | 0.28 | 0.448 at 90 | 0.904 at 150 |
| HCs | Any emotion | Reaction time | Presence of neutral faces (reference category, no) | 24 | 0.346 | -0.388, 1.08 | 0.36 | 0.285 at 0 | 0.631 at 1 |
| FDRs | Any emotion | Accuracy | Publication year | 11 | 0.02 | -0.01, 0.051 | 0.2 | -0.178 at 2010 | 0.064 at 2022 |
| FDRs | Any emotion | Accuracy | Primary outcome (reference category, no) | 11 | -0.188 | -0.459, 0.084 | 0.18 | 0.084 at 0 | -0.104 at 1 |
| FDRs | Any emotion | Accuracy | Age | 11 | -0.016 | -0.04, 0.008 | 0.19 | 0.096 at 28.42 | -0.186 at 46 |
| FDRs | Any emotion | Accuracy | % of females | 11 | 0.385 | -0.697, 1.467 | 0.48 | -0.161 at 0.23 | 0.039 at 0.75 |
| FDRs | Any emotion | Accuracy | Morphed (reference category, no) | 11 | 0.132 | -0.186, 0.451 | 0.42 | -0.073 at 0 | 0.06 at 1 |
| FDRs | Any emotion | Accuracy | Number of stimuli | 11 | 0.001 | -0.001, 0.003 | 0.31 | -0.098 at 19 | 0.101 at 250 |
| FDRs | Any emotion | Accuracy | Number of emotions | 11 | 0.028 | -0.059, 0.115 | 0.52 | -0.133 at 1 | 0.008 at 6 |
| FDRs | Any emotion | Accuracy | Practice (reference category, no) | 11 | -0.157 | -0.744, 0.43 | 0.6 | -0.028 at 0 | -0.185 at 1 |
| FDRs | Any emotion | Accuracy | Minimum emotion intensity | 11 | -0.00006 | -0.003, 0.003 | 0.97 | -0.034 at 10 | -0.042 at 150 |
| FDRs | Any emotion | Accuracy | Maximum emotion intensity | 11 | 0.002 | -0.005, 0.008 | 0.67 | -0.068 at 90 | 0.022 at 150 |
| FDRs | Any emotion | Accuracy | Presence of neutral faces (reference category, no) | 10 | 0.066 | -0.535, 0.667 | 0.83 | -0.144 at 0 | -0.078 at 1 |
| Level II | | | | | | | | | |
| HCs | Negative | Accuracy | Publication year | 45 | -0.009 | -0.034, 0.016 | 0.47 | -0.219 at 2004 | -0.395 at 2023 |
| HCs | Negative | Accuracy | Primary outcome (reference category, no) | 45 | -0.058 | -0.277, 0.161 | 0.6 | -0.291 at 0 | -0.349 at 1 |
| HCs | Negative | Accuracy | Age | 45 | 0.009 | -0.009, 0.026 | 0.33 | -0.476 at 20.03 | -0.216 at 50.38 |
| HCs | Negative | Accuracy | Education, years | 26 | 0.025 | -0.019, 0.068 | 0.26 | -0.587 at 3.46 | -0.232 at 17.7 |
| HCs | Negative | Accuracy | % of females | 43 | 0.23 | -0.509, 0.97 | 0.54 | -0.403 at 0.18 | -0.215 at 100 |
| HCs | Negative | Accuracy | Duration of illness | 18 | 0.021 | -0.011, 0.053 | 0.2 | -0.557 at 5.1 | -0.242 at 20.2 |
| HCs | Negative | Accuracy | Age at onset | 14 | -0.013 | -0.042, 0.017 | 0.39 | -0.218 at 16.53 | -0.49 at 37.9 |
| HCs | Negative | Accuracy | % of people with BD-I | 30 | -0.154 | -0.422, 0.115 | 0.26 | -0.188 at 0 | -0.342 at 100 |
| HCs | Negative | Accuracy | % of euthymic | 39 | -0.085 | -0.309, 0.14 | 0.46 | -0.308 at 0 | -0.393 at 100 |
| HCs | Negative | Accuracy | % of depressed | 36 | 0.165 | -0.135, 0.465 | 0.28 | -0.397 at 0 | -0.232 at 100 |
| HCs | Negative | Accuracy | % of (hypo)manic | 35 | -0.171 | -0.604, 0.263 | 0.44 | -0.365 at 0 | -0.536 at 100 |
| HCs | Negative | Accuracy | Symptoms severity scale, depression | 30 | 0.015 | -0.007, 0.036 | 0.17 | -0.346 at 2 | 0.01 at 26 |
| HCs | Negative | Accuracy | Symptoms severity scale, mania | 30 | 0.004 | -0.031, 0.039 | 0.82 | -0.292 at 0.7 | -0.231 at 15.75 |
| HCs | Negative | Accuracy | Morphed (reference category, no) | 45 | -0.086 | -0.362, 0.19 | 0.54 | -0.305 at 0 | -0.391 at 1 |
| HCs | Negative | Accuracy | Number of stimuli | 44 | 0.00001 | -0.002, 0.002 | 0.99 | -0.326 at 19 | -0.323 at 250 |
| HCs | Negative | Accuracy | Number of emotions | 45 | 0.028 | -0.041, 0.097 | 0.42 | -0.422 at 1 | -0.28 at 6 |
| HCs | Negative | Accuracy | Duration of stimulus (msec) | 32 | 0 | -5e-05, 4e-05 | 0.9 | -0.321 at 100 | -0.362 at 15000 |
| HCs | Negative | Accuracy | ISI, msec | 13 | -0.00001 | -6e-05, 5e-05 | 0.76 | -0.26 at 0 | -0.346 at 10000 |
| HCs | Negative | Accuracy | Practice (reference category, no) | 45 | 0.208 | -0.057, 0.473 | 0.12 | -0.362 at 0 | -0.154 at 1 |
| HCs | Negative | Accuracy | Minimum emotion intensity | 45 | -0.001 | -0.004, 0.001 | 0.29 | -0.217 at 3 | -0.417 at 150 |
| HCs | Negative | Accuracy | Maximum emotion intensity | 45 | -0.001 | -0.008, 0.007 | 0.86 | -0.306 at 80 | -0.353 at 150 |
| HCs | Negative | Accuracy | Presence of neutral faces (reference category, no) | 44 | 0.134 | -0.096, 0.365 | 0.25 | -0.403 at 0 | -0.268 at 1 |
| HCs | Negative | Reaction time | Publication year | 19 | -0.059 | -0.111, -0.008 | 0.024 | 0.968 at 2007 | 0.02 at 2023 |
| HCs | Negative | Reaction time | Primary outcome (reference category, no) | 19 | -0.213 | -0.805, 0.379 | 0.48 | 0.504 at 0 | 0.291 at 1 |
| HCs | Negative | Reaction time | Age | 19 | 0.037 | -0.005, 0.079 | 0.09 | -0.202 at 20.03 | 0.769 at 46.2 |
| HCs | Negative | Reaction time | Education, years | 11 | -0.041 | -0.135, 0.054 | 0.4 | 0.908 at 3.46 | 0.329 at 17.7 |
| HCs | Negative | Reaction time | % of females | 19 | 0.699 | -0.888, 2.287 | 0.39 | 0.186 at 0.19 | 0.752 at 100 |
| HCs | Negative | Reaction time | % of people with BD-I | 14 | 0.306 | -0.577, 1.188 | 0.5 | 0.366 at 0 | 0.672 at 100 |
| HCs | Negative | Reaction time | % of euthymic | 15 | 0.015 | -0.827, 0.857 | 0.97 | 0.567 at 0 | 0.581 at 100 |
| HCs | Negative | Reaction time | % of depressed | 14 | 0.282 | -1.581, 2.144 | 0.77 | 0.565 at 0 | 0.689 at 44 |
| HCs | Negative | Reaction time | Symptoms severity scale, depression | 13 | -0.057 | -0.2, 0.086 | 0.44 | 0.712 at 2.16 | 0.154 at 12 |
| HCs | Negative | Reaction time | Symptoms severity scale, mania | 14 | -0.148 | -0.278, -0.017 | 0.027 | 0.831 at 0.9 | -0.47 at 9.7 |
| HCs | Negative | Reaction time | Morphed (reference category, no) | 19 | 0.432 | -0.205, 1.069 | 0.18 | 0.321 at 0 | 0.753 at 1 |
| HCs | Negative | Reaction time | Number of stimuli | 18 | -0.002 | -0.006, 0.002 | 0.24 | 0.622 at 10 | 0.074 at 250 |
| HCs | Negative | Reaction time | Number of emotions | 19 | -0.139 | -0.294, 0.016 | 0.08 | 0.788 at 1 | 0.092 at 6 |
| HCs | Negative | Reaction time | Duration of stimulus (msec) | 15 | -0.00007 | -0.00019, 5e-05 | 0.26 | 0.626 at 300 | -0.059 at 10000 |
| HCs | Negative | Reaction time | Practice (reference category, no) | 19 | -0.596 | -1.163, -0.028 | 0.04 | 0.583 at 0 | -0.013 at 1 |
| HCs | Negative | Reaction time | Minimum emotion intensity | 19 | 0.001 | -0.004, 0.007 | 0.7 | 0.346 at 1 | 0.504 at 150 |
| HCs | Negative | Reaction time | Maximum emotion intensity | 19 | 0.011 | -0.003, 0.025 | 0.12 | 0.232 at 90 | 0.887 at 150 |
| HCs | Negative | Reaction time | Presence of neutral faces (reference category, no) | 19 | 0.16 | -0.745, 1.065 | 0.73 | 0.288 at 0 | 0.448 at 1 |
| HCs | Positive | Accuracy | Publication year | 39 | -0.016 | -0.04, 0.009 | 0.22 | -0.108 at 2004 | -0.404 at 2023 |
| HCs | Positive | Accuracy | Primary outcome (reference category, no) | 39 | -0.01 | -0.237, 0.216 | 0.93 | -0.269 at 0 | -0.28 at 1 |
| HCs | Positive | Accuracy | Age | 39 | -0.001 | -0.019, 0.016 | 0.9 | -0.255 at 20.03 | -0.29 at 50.38 |
| HCs | Positive | Accuracy | Education, years | 22 | 0.064 | -0.019, 0.147 | 0.13 | -0.396 at 10.31 | 0.075 at 17.7 |
| HCs | Positive | Accuracy | % of females | 37 | 0.53 | -0.284, 1.343 | 0.2 | -0.485 at 0.18 | -0.05 at 100 |
| HCs | Positive | Accuracy | Duration of illness | 14 | 0.041 | -0.004, 0.086 | 0.07 | -0.575 at 5.1 | -0.131 at 15.82 |
| HCs | Positive | Accuracy | Age at onset | 12 | -0.009 | -0.042, 0.023 | 0.58 | -0.135 at 16.53 | -0.335 at 37.9 |
| HCs | Positive | Accuracy | % of people with BD-I | 26 | -0.092 | -0.443, 0.259 | 0.61 | -0.206 at 0 | -0.298 at 100 |
| HCs | Positive | Accuracy | % of euthymic | 32 | -0.08 | -0.332, 0.172 | 0.53 | -0.201 at 0 | -0.281 at 100 |
| HCs | Positive | Accuracy | % of depressed | 30 | 0.023 | -0.363, 0.41 | 0.91 | -0.276 at 0 | -0.252 at 100 |
| HCs | Positive | Accuracy | % of (hypo)manic | 29 | -0.043 | -0.554, 0.467 | 0.87 | -0.285 at 0 | -0.328 at 100 |
| HCs | Positive | Accuracy | Symptoms severity scale, depression | 25 | -0.001 | -0.024, 0.023 | 0.95 | -0.26 at 2 | -0.277 at 26 |
| HCs | Positive | Accuracy | Symptoms severity scale, mania | 25 | -0.024 | -0.06, 0.011 | 0.17 | -0.149 at 0.7 | -0.517 at 15.75 |
| HCs | Positive | Accuracy | Morphed (reference category, no) | 39 | 0.075 | -0.189, 0.34 | 0.58 | -0.293 at 0 | -0.218 at 1 |
| HCs | Positive | Accuracy | Number of stimuli | 38 | -0.00003 | -0.002, 0.002 | 0.97 | -0.274 at 18 | -0.281 at 250 |
| HCs | Positive | Accuracy | Number of emotions | 39 | -0.011 | -0.094, 0.071 | 0.78 | -0.242 at 2 | -0.288 at 6 |
| HCs | Positive | Accuracy | Duration of stimulus (msec) | 26 | -0.00003 | -8e-05, 1e-05 | 0.13 | -0.198 at 100 | -0.702 at 15000 |
| HCs | Positive | Accuracy | ISI, msec | 10 | -0.00001 | -8e-05, 5e-05 | 0.74 | -0.194 at 0 | -0.306 at 10000 |
| HCs | Positive | Accuracy | Practice (reference category, no) | 39 | -0.217 | -0.468, 0.035 | 0.09 | -0.225 at 0 | -0.442 at 1 |
| HCs | Positive | Accuracy | Minimum emotion intensity | 39 | 0.001 | -0.002, 0.004 | 0.39 | -0.355 at 3 | -0.24 at 100 |
| HCs | Positive | Accuracy | Maximum emotion intensity | 39 | -0.005 | -0.02, 0.01 | 0.49 | -0.169 at 80 | -0.54 at 150 |
| HCs | Positive | Accuracy | Presence of neutral faces (reference category, no) | 38 | -0.125 | -0.359, 0.109 | 0.29 | -0.192 at 0 | -0.317 at 1 |
| HCs | Positive | Reaction time | Publication year | 13 | -0.019 | -0.094, 0.056 | 0.62 | 0.769 at 2009 | 0.525 at 2022 |
| HCs | Positive | Reaction time | Primary outcome (reference category, no) | 13 | -0.068 | -0.737, 0.6 | 0.84 | 0.645 at 0 | 0.576 at 1 |
| HCs | Positive | Reaction time | Age | 13 | 0.048 | 0.01, 0.085 | 0.013 | -0.141 at 20.03 | 1.11 at 46.2 |
| HCs | Positive | Reaction time | % of females | 13 | 0.069 | -2.011, 2.149 | 0.95 | 0.599 at 0.19 | 0.638 at 75 |
| HCs | Positive | Reaction time | Morphed (reference category, no) | 13 | 0.313 | -0.311, 0.936 | 0.33 | 0.513 at 0 | 0.825 at 1 |
| HCs | Positive | Reaction time | Number of stimuli | 12 | -0.003 | -0.006, -0.0001 | 0.023 | 0.944 at 10 | 0.155 at 250 |
| HCs | Positive | Reaction time | Number of emotions | 13 | 0.037 | -0.157, 0.231 | 0.71 | 0.542 at 2 | 0.692 at 6 |
| HCs | Positive | Reaction time | Duration of stimulus (msec) | 10 | 0.00014 | 2e-05, 0.00025 | 0.024 | 0.368 at 300 | 1.417 at 8000 |
| HCs | Positive | Reaction time | Practice (reference category, no) | 13 | -0.202 | -0.821, 0.416 | 0.52 | 0.704 at 0 | 0.502 at 1 |
| HCs | Positive | Reaction time | Minimum emotion intensity | 13 | -0.005 | -0.011, 0.001 | 0.08 | 0.933 at 1 | 0.399 at 100 |
| HCs | Positive | Reaction time | Maximum emotion intensity | 13 | -0.00049 | -0.114, 0.113 | 0.99 | 0.63 at 90 | 0.625 at 100 |
| HCs | Positive | Reaction time | Presence of neutral faces (reference category, no) | 13 | 0.314 | -0.746, 1.374 | 0.56 | 0.338 at 0 | 0.652 at 1 |
| Level III | | | | | | | | | |
| HCs | Anger | Reaction time | Publication year | 11 | -0.045 | -0.121, 0.031 | 0.25 | 0.676 at 2013 | 0.229 at 2023 |
| HCs | Anger | Reaction time | Primary outcome (reference category, no) | 11 | -0.098 | -0.82, 0.624 | 0.79 | 0.501 at 0 | 0.403 at 1 |
| HCs | Anger | Reaction time | Age | 11 | 0.025 | -0.019, 0.069 | 0.26 | 0.064 at 20.03 | 0.719 at 46.2 |
| HCs | Anger | Reaction time | % of females | 11 | 0.757 | -1.091, 2.606 | 0.42 | 0.217 at 0.19 | 0.641 at 75 |
| HCs | Anger | Reaction time | Morphed (reference category, no) | 11 | 0.444 | -0.148, 1.036 | 0.14 | 0.282 at 0 | 0.726 at 1 |
| HCs | Anger | Reaction time | Number of stimuli | 10 | -0.002 | -0.006, 0.002 | 0.22 | 0.714 at 10 | 0.124 at 250 |
| HCs | Anger | Reaction time | Number of emotions | 11 | -0.16 | -0.371, 0.05 | 0.13 | 0.879 at 2 | 0.237 at 6 |
| HCs | Anger | Reaction time | Practice (reference category, no) | 11 | -0.176 | -0.822, 0.47 | 0.59 | 0.542 at 0 | 0.366 at 1 |
| HCs | Anger | Reaction time | Minimum emotion intensity | 11 | -0.004 | -0.011, 0.003 | 0.23 | 0.678 at 1 | 0.276 at 100 |
| HCs | Anger | Reaction time | Maximum emotion intensity | 11 | 0.007 | -0.102, 0.116 | 0.9 | 0.413 at 90 | 0.483 at 100 |
| HCs | Disgust | Accuracy | Publication year | 22 | 0.012 | -0.014, 0.039 | 0.37 | -0.475 at 2004 | -0.243 at 2023 |
| HCs | Disgust | Accuracy | Primary outcome (reference category, no) | 22 | -0.009 | -0.282, 0.264 | 0.95 | -0.322 at 0 | -0.332 at 1 |
| HCs | Disgust | Accuracy | Age | 22 | -0.004 | -0.026, 0.018 | 0.72 | -0.283 at 28.16 | -0.371 at 50.38 |
| HCs | Disgust | Accuracy | Education, years | 13 | 0.138 | 0.016, 0.26 | 0.027 | -0.565 at 12.2 | 0.194 at 17.7 |
| HCs | Disgust | Accuracy | % of females | 21 | -1.161 | -2.098, -0.223 | 0.015 | -0.053 at 0.33 | -0.83 at 100 |
| HCs | Disgust | Accuracy | % of people with BD-I | 14 | 0.123 | -0.545, 0.791 | 0.72 | -0.42 at 0 | -0.298 at 100 |
| HCs | Disgust | Accuracy | % of euthymic | 19 | -0.135 | -0.49, 0.219 | 0.46 | -0.291 at 0 | -0.427 at 100 |
| HCs | Disgust | Accuracy | % of depressed | 18 | 0.224 | -0.214, 0.662 | 0.32 | -0.436 at 0 | -0.213 at 100 |
| HCs | Disgust | Accuracy | % of (hypo)manic | 18 | -0.083 | -0.781, 0.615 | 0.81 | -0.375 at 0 | -0.458 at 100 |
| HCs | Disgust | Accuracy | Symptoms severity scale, depression | 18 | 0.012 | -0.006, 0.031 | 0.18 | -0.39 at 2.06 | -0.092 at 26 |
| HCs | Disgust | Accuracy | Symptoms severity scale, mania | 18 | 0.023 | -0.015, 0.061 | 0.24 | -0.377 at 0.7 | -0.092 at 13.11 |
| HCs | Disgust | Accuracy | Morphed (reference category, no) | 22 | 0.157 | -0.122, 0.436 | 0.27 | -0.376 at 0 | -0.219 at 1 |
| HCs | Disgust | Accuracy | Number of stimuli | 22 | 0.001 | -0.001, 0.003 | 0.2 | -0.4 at 30 | -0.143 at 250 |
| HCs | Disgust | Accuracy | Number of emotions | 22 | -0.051 | -0.24, 0.137 | 0.59 | -0.19 at 3 | -0.344 at 6 |
| HCs | Disgust | Accuracy | Duration of stimulus (msec) | 15 | 0.00003 | -5e-05, 0.00011 | 0.45 | -0.405 at 100 | -0.168 at 8000 |
| HCs | Disgust | Accuracy | Practice (reference category, no) | 22 | 0.08 | -0.211, 0.371 | 0.59 | -0.353 at 0 | -0.273 at 1 |
| HCs | Disgust | Accuracy | Minimum emotion intensity | 22 | -0.002 | -0.005, 0.001 | 0.15 | -0.209 at 3 | -0.411 at 100 |
| HCs | Disgust | Accuracy | Maximum emotion intensity | 22 | 0.007 | -0.006, 0.021 | 0.3 | -0.475 at 80 | 0.041 at 150 |
| HCs | Disgust | Accuracy | Presence of neutral faces (reference category, no) | 22 | 0.033 | -0.244, 0.309 | 0.82 | -0.347 at 0 | -0.315 at 1 |
| HCs | Fear | Accuracy | Publication year | 33 | 0.002 | -0.028, 0.032 | 0.87 | -0.392 at 2004 | -0.346 at 2023 |
| HCs | Fear | Accuracy | Primary outcome (reference category, no) | 33 | -0.149 | -0.393, 0.096 | 0.23 | -0.279 at 0 | -0.427 at 1 |
| HCs | Fear | Accuracy | Age | 33 | 0.002 | -0.019, 0.023 | 0.86 | -0.388 at 26.65 | -0.345 at 50.38 |
| HCs | Fear | Accuracy | Education, years | 21 | 0.01 | -0.043, 0.062 | 0.72 | -0.456 at 3.46 | -0.319 at 17.7 |
| HCs | Fear | Accuracy | % of females | 31 | 0.154 | -0.721, 1.029 | 0.73 | -0.408 at 0.18 | -0.281 at 100 |
| HCs | Fear | Accuracy | Age at onset | 10 | -0.007 | -0.052, 0.038 | 0.76 | -0.336 at 16.53 | -0.487 at 37.9 |
| HCs | Fear | Accuracy | % of people with BD-I | 21 | 0.051 | -0.584, 0.686 | 0.88 | -0.509 at 0 | -0.457 at 100 |
| HCs | Fear | Accuracy | % of euthymic | 28 | -0.081 | -0.442, 0.281 | 0.66 | -0.366 at 0 | -0.447 at 100 |
| HCs | Fear | Accuracy | % of depressed | 26 | 0.163 | -0.344, 0.671 | 0.53 | -0.482 at 0 | -0.318 at 100 |
| HCs | Fear | Accuracy | % of (hypo)manic | 26 | -0.288 | -0.959, 0.382 | 0.4 | -0.424 at 0 | -0.713 at 100 |
| HCs | Fear | Accuracy | Symptoms severity scale, depression | 23 | 0.008 | -0.016, 0.031 | 0.53 | -0.358 at 2 | -0.175 at 26 |
| HCs | Fear | Accuracy | Symptoms severity scale, mania | 23 | -0.002 | -0.038, 0.034 | 0.92 | -0.312 at 0.7 | -0.338 at 15.75 |
| HCs | Fear | Accuracy | Morphed (reference category, no) | 33 | 0.04 | -0.253, 0.333 | 0.79 | -0.376 at 0 | -0.336 at 1 |
| HCs | Fear | Accuracy | Number of stimuli | 33 | 0.001 | -0.001, 0.003 | 0.26 | -0.426 at 19 | -0.182 at 250 |
| HCs | Fear | Accuracy | Number of emotions | 33 | -0.068 | -0.163, 0.027 | 0.16 | -0.089 at 1 | -0.429 at 6 |
| HCs | Fear | Accuracy | Duration of stimulus (msec) | 23 | -0.00001 | -4e-05, 2e-05 | 0.48 | -0.203 at 100 | -0.375 at 15000 |
| HCs | Fear | Accuracy | Practice (reference category, no) | 33 | 0.22 | -0.081, 0.52 | 0.15 | -0.412 at 0 | -0.192 at 1 |
| HCs | Fear | Accuracy | Minimum emotion intensity | 33 | -0.001 | -0.004, 0.002 | 0.66 | -0.322 at 3 | -0.418 at 150 |
| HCs | Fear | Accuracy | Maximum emotion intensity | 33 | 0.00011 | -0.01, 0.011 | 0.98 | -0.369 at 80 | -0.361 at 150 |
| HCs | Fear | Accuracy | Presence of neutral faces (reference category, no) | 32 | 0.128 | -0.137, 0.392 | 0.34 | -0.442 at 0 | -0.314 at 1 |
| HCs | Fear | Reaction time | Publication year | 11 | -0.046 | -0.068, -0.025 | <0.001 | 0.79 at 2007 | 0.096 at 2022 |
| HCs | Fear | Reaction time | Primary outcome (reference category, no) | 11 | -0.095 | -0.537, 0.347 | 0.67 | 0.41 at 0 | 0.315 at 1 |
| HCs | Fear | Reaction time | Age | 11 | 0.024 | -0.016, 0.064 | 0.23 | 0.232 at 31.2 | 0.592 at 46.2 |
| HCs | Fear | Reaction time | % of females | 11 | 0.383 | -0.757, 1.522 | 0.51 | 0.232 at 0.19 | 0.542 at 100 |
| HCs | Fear | Reaction time | Morphed (reference category, no) | 11 | 0.01 | -0.397, 0.418 | 0.96 | 0.382 at 0 | 0.393 at 1 |
| HCs | Fear | Reaction time | Number of stimuli | 11 | -0.001 | -0.003, 0.001 | 0.2 | 0.466 at 30 | 0.179 at 250 |
| HCs | Fear | Reaction time | Number of emotions | 11 | -0.08 | -0.166, 0.006 | 0.07 | 0.64 at 1 | 0.24 at 6 |
| HCs | Fear | Reaction time | Practice (reference category, no) | 11 | -0.194 | -0.605, 0.216 | 0.35 | 0.438 at 0 | 0.244 at 1 |
| HCs | Fear | Reaction time | Minimum emotion intensity | 11 | 0.002 | -0.001, 0.005 | 0.12 | 0.236 at 5 | 0.568 at 150 |
| HCs | Fear | Reaction time | Maximum emotion intensity | 11 | 0.006 | -0.006, 0.018 | 0.31 | 0.294 at 90 | 0.663 at 150 |
| HCs | Happiness | Accuracy | Publication year | 35 | 0.006 | -0.013, 0.026 | 0.53 | -0.261 at 2004 | -0.143 at 2023 |
| HCs | Happiness | Accuracy | Primary outcome (reference category, no) | 34 | -0.016 | -0.187, 0.154 | 0.85 | -0.175 at 0 | -0.191 at 1 |
| HCs | Happiness | Accuracy | Age | 35 | -0.002 | -0.015, 0.01 | 0.73 | -0.154 at 20.03 | -0.221 at 50.38 |
| HCs | Happiness | Accuracy | Education, years | 21 | 0.075 | 0.006, 0.144 | 0.033 | -0.405 at 10.31 | 0.149 at 17.7 |
| HCs | Happiness | Accuracy | % of females | 33 | 0.189 | -0.486, 0.865 | 0.58 | -0.267 at 0.18 | -0.146 at 82 |
| HCs | Happiness | Accuracy | Duration of illness | 11 | 0.021 | -0.054, 0.096 | 0.58 | -0.47 at 5.49 | -0.249 at 15.82 |
| HCs | Happiness | Accuracy | Age at onset | 10 | -0.013 | -0.045, 0.02 | 0.45 | -0.148 at 16.53 | -0.416 at 37.9 |
| HCs | Happiness | Accuracy | % of people with BD-I | 24 | -0.391 | -0.707, -0.075 | 0.015 | 0.064 at 0 | -0.327 at 100 |
| HCs | Happiness | Accuracy | % of euthymic | 29 | -0.12 | -0.357, 0.118 | 0.32 | -0.166 at 0 | -0.286 at 100 |
| HCs | Happiness | Accuracy | % of depressed | 27 | 0.037 | -0.334, 0.408 | 0.85 | -0.27 at 0 | -0.233 at 100 |
| HCs | Happiness | Accuracy | % of (hypo)manic | 26 | 0.088 | -0.406, 0.581 | 0.73 | -0.291 at 0 | -0.203 at 100 |
| HCs | Happiness | Accuracy | Symptoms severity scale, depression | 23 | 0.003 | -0.011, 0.018 | 0.64 | -0.183 at 2 | -0.1 at 26 |
| HCs | Happiness | Accuracy | Symptoms severity scale, mania | 23 | -0.001 | -0.02, 0.019 | 0.95 | -0.129 at 0.7 | -0.138 at 15.75 |
| HCs | Happiness | Accuracy | Morphed (reference category, no) | 35 | 0.004 | -0.195, 0.204 | 0.97 | -0.194 at 0 | -0.19 at 1 |
| HCs | Happiness | Accuracy | Number of stimuli | 34 | -0.00008 | -0.001, 0.001 | 0.91 | -0.19 at 19 | -0.208 at 250 |
| HCs | Happiness | Accuracy | Number of emotions | 35 | -0.019 | -0.082, 0.043 | 0.55 | -0.136 at 2 | -0.213 at 6 |
| HCs | Happiness | Accuracy | Duration of stimulus (msec) | 24 | 0 | -4e-05, 3e-05 | 0.77 | -0.178 at 100 | -0.248 at 15000 |
| HCs | Happiness | Accuracy | Practice (reference category, no) | 35 | -0.023 | -0.218, 0.171 | 0.81 | -0.187 at 0 | -0.211 at 1 |
| HCs | Happiness | Accuracy | Minimum emotion intensity | 35 | -0.00025 | -0.002, 0.002 | 0.81 | -0.176 at 3 | -0.2 at 100 |
| HCs | Happiness | Accuracy | Maximum emotion intensity | 35 | -0.006 | -0.018, 0.006 | 0.32 | -0.067 at 80 | -0.501 at 150 |
| HCs | Happiness | Accuracy | Presence of neutral faces (reference category, no) | 34 | -0.016 | -0.187, 0.154 | 0.85 | -0.175 at 0 | -0.191 at 1 |
| HCs | Happiness | Reaction time | Publication year | 12 | -0.032 | -0.107, 0.042 | 0.4 | 0.788 at 2009 | 0.37 at 2022 |
| HCs | Happiness | Reaction time | Primary outcome (reference category, no) | 12 | 0.328 | -0.352, 1.009 | 0.34 | 0.477 at 0 | 0.806 at 1 |
| HCs | Happiness | Reaction time | Age | 12 | 0.043 | 0.004, 0.081 | 0.029 | -0.134 at 20.03 | 0.986 at 46.2 |
| HCs | Happiness | Reaction time | % of females | 12 | -0.113 | -2.15, 1.925 | 0.91 | 0.601 at 0.19 | 0.538 at 75 |
| HCs | Happiness | Reaction time | Morphed (reference category, no) | 12 | 0.4 | -0.198, 0.998 | 0.19 | 0.401 at 0 | 0.801 at 1 |
| HCs | Happiness | Reaction time | Number of stimuli | 11 | -0.002 | -0.005, 0.002 | 0.35 | 0.742 at 10 | 0.325 at 250 |
| HCs | Happiness | Reaction time | Number of emotions | 12 | -0.091 | -0.294, 0.112 | 0.38 | 0.77 at 2 | 0.408 at 6 |
| HCs | Happiness | Reaction time | Practice (reference category, no) | 12 | -0.347 | -0.95, 0.255 | 0.26 | 0.671 at 0 | 0.324 at 1 |
| HCs | Happiness | Reaction time | Minimum emotion intensity | 12 | -0.003 | -0.009, 0.004 | 0.46 | 0.698 at 1 | 0.447 at 100 |
| HCs | Happiness | Reaction time | Maximum emotion intensity | 12 | -0.008 | -0.118, 0.102 | 0.89 | 0.63 at 90 | 0.553 at 100 |
| HCs | Happiness | Reaction time | Presence of neutral faces (reference category, no) | 12 | 0.244 | -0.799, 1.288 | 0.65 | 0.338 at 0 | 0.582 at 1 |
| HCs | Neutral | Reaction time | Publication year | 10 | -0.033 | -0.069, 0.003 | 0.07 | 0.612 at 2007 | 0.081 at 2023 |
| HCs | Neutral | Reaction time | Primary outcome (reference category, no) | 10 | 0.127 | -0.325, 0.579 | 0.58 | 0.259 at 0 | 0.385 at 1 |
| HCs | Neutral | Reaction time | Age | 10 | 0.009 | -0.021, 0.04 | 0.55 | 0.159 at 20.03 | 0.403 at 46.2 |
| HCs | Neutral | Reaction time | % of females | 10 | 0.586 | -0.501, 1.672 | 0.29 | 0.078 at 0.19 | 0.553 at 100 |
| HCs | Neutral | Reaction time | Morphed (reference category, no) | 10 | -0.203 | -0.589, 0.184 | 0.3 | 0.356 at 0 | 0.153 at 1 |
| HCs | Neutral | Reaction time | Number of emotions | 10 | -0.085 | -0.222, 0.053 | 0.23 | 0.472 at 2 | 0.134 at 6 |
| HCs | Neutral | Reaction time | Practice (reference category, no) | 10 | 0.054 | -0.4, 0.507 | 0.82 | 0.279 at 0 | 0.333 at 1 |
| HCs | Neutral | Reaction time | Minimum emotion intensity | 10 | 0.002 | -0.002, 0.007 | 0.3 | 0.153 at 10 | 0.356 at 100 |
| HCs | Neutral | Reaction time | Maximum emotion intensity | 10 | -0.001 | -0.069, 0.068 | 0.98 | 0.3 at 90 | 0.293 at 100 |
| HCs | Sadness | Accuracy | Publication year | 36 | 0.007 | -0.017, 0.031 | 0.54 | -0.309 at 2004 | -0.167 at 2023 |
| HCs | Sadness | Accuracy | Primary outcome (reference category, no) | 36 | 0.067 | -0.147, 0.282 | 0.54 | -0.265 at 0 | -0.197 at 1 |
| HCs | Sadness | Accuracy | Age | 36 | 0.017 | 0.002, 0.033 | 0.031 | -0.534 at 20.03 | -0.013 at 50.38 |
| HCs | Sadness | Accuracy | Education, years | 21 | 0.082 | 0.008, 0.157 | 0.031 | -0.498 at 10.31 | 0.11 at 17.7 |
| HCs | Sadness | Accuracy | % of females | 35 | -0.088 | -0.795, 0.619 | 0.81 | -0.186 at 0.18 | -0.258 at 100 |
| HCs | Sadness | Accuracy | Duration of illness | 12 | -0.004 | -0.073, 0.064 | 0.91 | -0.201 at 5.49 | -0.26 at 19.94 |
| HCs | Sadness | Accuracy | Age at onset | 10 | -0.026 | -0.076, 0.024 | 0.31 | -0.047 at 16.9 | -0.592 at 37.9 |
| HCs | Sadness | Accuracy | % of people with BD-I | 25 | -0.075 | -0.614, 0.464 | 0.78 | -0.23 at 0 | -0.305 at 100 |
| HCs | Sadness | Accuracy | % of euthymic | 30 | 0.069 | -0.253, 0.391 | 0.68 | -0.31 at 0 | -0.241 at 100 |
| HCs | Sadness | Accuracy | % of depressed | 28 | 0.04 | -0.402, 0.481 | 0.86 | -0.296 at 0 | -0.256 at 100 |
| HCs | Sadness | Accuracy | % of (hypo)manic | 28 | -0.563 | -1.158, 0.033 | 0.06 | -0.238 at 0 | -0.8 at 100 |
| HCs | Sadness | Accuracy | Symptoms severity scale, depression | 26 | 0.001 | -0.017, 0.02 | 0.89 | -0.184 at 2 | -0.153 at 26 |
| HCs | Sadness | Accuracy | Symptoms severity scale, mania | 25 | -0.003 | -0.033, 0.026 | 0.82 | -0.169 at 0.7 | -0.22 at 15.75 |
| HCs | Sadness | Accuracy | Morphed (reference category, no) | 36 | -0.071 | -0.322, 0.179 | 0.58 | -0.21 at 0 | -0.281 at 1 |
| HCs | Sadness | Accuracy | Number of stimuli | 35 | -0.00018 | -0.002, 0.001 | 0.83 | -0.217 at 19 | -0.259 at 250 |
| HCs | Sadness | Accuracy | Number of emotions | 36 | 0.005 | -0.071, 0.081 | 0.9 | -0.245 at 1 | -0.22 at 6 |
| HCs | Sadness | Accuracy | Duration of stimulus (msec) | 24 | 0.00002 | -1e-05, 4e-05 | 0.26 | -0.233 at 100 | 0.001 at 15000 |
| HCs | Sadness | Accuracy | Practice (reference category, no) | 36 | 0.129 | -0.116, 0.374 | 0.3 | -0.254 at 0 | -0.126 at 1 |
| HCs | Sadness | Accuracy | Minimum emotion intensity | 36 | -0.00038 | -0.003, 0.002 | 0.76 | -0.199 at 3 | -0.255 at 150 |
| HCs | Sadness | Accuracy | Maximum emotion intensity | 36 | -0.001 | -0.011, 0.008 | 0.77 | -0.196 at 80 | -0.292 at 150 |
| HCs | Sadness | Accuracy | Presence of neutral faces (reference category, no) | 35 | 0.02 | -0.214, 0.253 | 0.87 | -0.237 at 0 | -0.217 at 1 |
| HCs | Sadness | Reaction time | Publication year | 11 | -0.064 | -0.088, -0.04 | <0.001 | 0.773 at 2010 | -0.059 at 2023 |
| HCs | Sadness | Reaction time | Primary outcome (reference category, no) | 11 | 0.166 | -0.274, 0.606 | 0.46 | 0.279 at 0 | 0.445 at 1 |
| HCs | Sadness | Reaction time | Age | 11 | 0.012 | -0.019, 0.043 | 0.45 | 0.14 at 20.03 | 0.451 at 46.2 |
| HCs | Sadness | Reaction time | % of females | 11 | 0.723 | -0.623, 2.068 | 0.29 | 0.083 at 0.19 | 0.488 at 75 |
| HCs | Sadness | Reaction time | Morphed (reference category, no) | 11 | 0.08 | -0.38, 0.539 | 0.73 | 0.307 at 0 | 0.387 at 1 |
| HCs | Sadness | Reaction time | Number of stimuli | 10 | -0.002 | -0.004, 0.001 | 0.16 | 0.449 at 36 | 0.074 at 250 |
| HCs | Sadness | Reaction time | Number of emotions | 11 | -0.061 | -0.184, 0.062 | 0.33 | 0.536 at 1 | 0.231 at 6 |
| HCs | Sadness | Reaction time | Practice (reference category, no) | 11 | -0.054 | -0.495, 0.388 | 0.81 | 0.353 at 0 | 0.299 at 1 |
| HCs | Sadness | Reaction time | Minimum emotion intensity | 11 | 0.001 | -0.003, 0.005 | 0.6 | 0.266 at 5 | 0.423 at 150 |
| HCs | Sadness | Reaction time | Maximum emotion intensity | 11 | 0.007 | -0.007, 0.02 | 0.33 | 0.237 at 90 | 0.642 at 150 |
| ***Discrimination*** | | | | | | | | | |
| *Adults* | | | | | | | | | |
| Level I | | | | | | | | | |
| HCs | Any emotion | Accuracy | Publication year | 16 | -0.005 | -0.03, 0.02 | 0.71 | -0.511 at 1996 | -0.634 at 2022 |
| HCs | Any emotion | Accuracy | Primary outcome (reference category, no) | 16 | -0.108 | -0.5, 0.285 | 0.59 | -0.527 at 0 | -0.635 at 1 |
| HCs | Any emotion | Accuracy | Age | 16 | 0.015 | -0.022, 0.052 | 0.42 | -0.741 at 25.3 | -0.488 at 42.3 |
| HCs | Any emotion | Accuracy | Education, years | 11 | 0.181 | 0.035, 0.328 | 0.015 | -1.091 at 10.31 | -0.261 at 14.89 |
| HCs | Any emotion | Accuracy | % of females | 16 | -0.817 | -2.287, 0.652 | 0.28 | -0.322 at 0.23 | -0.747 at 75 |
| HCs | Any emotion | Accuracy | Duration of illness | 11 | 0.041 | -0.001, 0.084 | 0.06 | -0.928 at 3.8 | -0.209 at 21.2 |
| HCs | Any emotion | Accuracy | % of euthymic | 13 | -0.375 | -0.815, 0.066 | 0.1 | -0.4 at 0 | -0.774 at 100 |
| HCs | Any emotion | Accuracy | % of depressed | 12 | 0.667 | 0.206, 1.127 | 0.005 | -0.757 at 0 | -0.09 at 100 |
| HCs | Any emotion | Accuracy | % of (hypo)manic | 12 | 0.186 | -0.619, 0.992 | 0.65 | -0.631 at 0 | -0.445 at 100 |
| HCs | Any emotion | Accuracy | Symptoms severity scale, mania | 11 | -0.004 | -0.029, 0.021 | 0.74 | -0.531 at 0.72 | -0.647 at 28.1 |
| HCs | Any emotion | Accuracy | Morphed (reference category, no) | 16 | -0.607 | -1.528, 0.314 | 0.2 | -0.564 at 0 | -1.171 at 1 |
| HCs | Any emotion | Accuracy | Number of stimuli | 16 | -0.003 | -0.011, 0.005 | 0.52 | -0.528 at 12 | -0.784 at 108 |
| HCs | Any emotion | Accuracy | Number of emotions | 15 | -0.003 | -0.115, 0.109 | 0.96 | -0.61 at 2 | -0.622 at 6 |
| HCs | Any emotion | Accuracy | Practice (reference category, no) | 16 | 0.052 | -0.439, 0.543 | 0.84 | -0.601 at 0 | -0.549 at 1 |
| HCs | Any emotion | Accuracy | Minimum emotion intensity | 16 | 0.009 | -0.005, 0.023 | 0.2 | -1.171 at 33 | -0.564 at 100 |
| HCs | Any emotion | Accuracy | Presence of neutral faces (reference category, no) | 14 | -0.013 | -0.472, 0.446 | 0.96 | -0.577 at 0 | -0.59 at 1 |

## Sensitivity analyses, leave-one-out sensitivity analysis

**eTable n. 7 - Leave-one-out sensitivity analysis**

The results are highlighted in green when the removal of that particular study changes a previously non-significant association to significant.

The results are highlighted in red when the removal of that particular study changes a previously significant association to not significant.

| **Control group** | **Emotion type** | **Outcome type** | **Author, year of the study removed** | **SMD** | **95% CIs** | **p-value** | **I^2^** | **tau^2^** | **Q test p-value** |
| --- | --- | --- | --- | --- | --- | --- | --- | --- | --- |
| ***Identification*** | | | | | | | | | |
| *Adults* | | | | | | | | | |
| Level I | | | | | | | | | |
| HCs | Any emotion | Accuracy | Addington et al., 1998 | -0.467 | -0.557, -0.376 | <0.001 | 64.23 | 0.08 | <0.1 |
| HCs | Any emotion | Accuracy | Almeida et al., 2010 | -0.467 | -0.557, -0.377 | <0.001 | 64.18 | 0.08 | <0.1 |
| HCs | Any emotion | Accuracy | Altamura et al., 2016 | -0.467 | -0.557, -0.377 | <0.001 | 64.17 | 0.08 | <0.1 |
| HCs | Any emotion | Accuracy | Barbosa et al., 2023 | -0.447 | -0.53, -0.363 | <0.001 | 57.44 | 0.06 | <0.1 |
| HCs | Any emotion | Accuracy | Bellack et al., 1996 | -0.467 | -0.557, -0.377 | <0.001 | 64.12 | 0.08 | <0.1 |
| HCs | Any emotion | Accuracy | Benito et al., 2013 | -0.454 | -0.541, -0.367 | <0.001 | 61.12 | 0.07 | <0.1 |
| HCs | Any emotion | Accuracy | Bjertrup et al., 2021 | -0.463 | -0.553, -0.374 | <0.001 | 63.81 | 0.08 | <0.1 |
| HCs | Any emotion | Accuracy | Branco et al., 2017 | -0.468 | -0.559, -0.378 | <0.001 | 64.22 | 0.08 | <0.1 |
| HCs | Any emotion | Accuracy | Darke et al., 2021 | -0.454 | -0.541, -0.368 | <0.001 | 61.34 | 0.07 | <0.1 |
| HCs | Any emotion | Accuracy | Derntl et al., 2009 | -0.466 | -0.557, -0.375 | <0.001 | 64.16 | 0.08 | <0.1 |
| HCs | Any emotion | Accuracy | Dima et al., 2016 | -0.464 | -0.555, -0.374 | <0.001 | 64.04 | 0.08 | <0.1 |
| HCs | Any emotion | Accuracy | Fernandes et al., 2016 | -0.468 | -0.559, -0.378 | <0.001 | 64.19 | 0.08 | <0.1 |
| HCs | Any emotion | Accuracy | Foland-Ross et al., 2012 | -0.473 | -0.562, -0.383 | <0.001 | 63.48 | 0.08 | <0.1 |
| HCs | Any emotion | Accuracy | Getz et al., 2003 | -0.46 | -0.549, -0.371 | <0.001 | 63.23 | 0.07 | <0.1 |
| HCs | Any emotion | Accuracy | Golkhatmi et al., 2013 | -0.46 | -0.549, -0.371 | <0.001 | 63.18 | 0.07 | <0.1 |
| HCs | Any emotion | Accuracy | Baez et al., 2013 | -0.462 | -0.552, -0.373 | <0.001 | 63.69 | 0.08 | <0.1 |
| HCs | Any emotion | Accuracy | Bora et al., 2005 | -0.469 | -0.56, -0.379 | <0.001 | 64.14 | 0.08 | <0.1 |
| HCs | Any emotion | Accuracy | Derntl et al., 2012 | -0.466 | -0.556, -0.375 | <0.001 | 64.15 | 0.08 | <0.1 |
| HCs | Any emotion | Accuracy | Goghari et al., 2013 | -0.472 | -0.561, -0.382 | <0.001 | 63.71 | 0.08 | <0.1 |
| HCs | Any emotion | Accuracy | Pan et al., 2013 | -0.466 | -0.557, -0.376 | <0.001 | 64.22 | 0.08 | <0.1 |
| HCs | Any emotion | Accuracy | Ryan et al., 2013 | -0.469 | -0.56, -0.378 | <0.001 | 63.65 | 0.08 | <0.1 |
| HCs | Any emotion | Accuracy | Vaskinn et al., 2007 | -0.472 | -0.562, -0.382 | <0.001 | 63.74 | 0.08 | <0.1 |
| HCs | Any emotion | Accuracy | Summers et al., 2006 | -0.47 | -0.56, -0.38 | <0.001 | 64.07 | 0.08 | <0.1 |
| HCs | Any emotion | Accuracy | Kjaerstad et al., 2022 | -0.473 | -0.564, -0.382 | <0.001 | 62.48 | 0.08 | <0.1 |
| HCs | Any emotion | Accuracy | Harmer et al., 2002 | -0.473 | -0.562, -0.384 | <0.001 | 63.33 | 0.08 | <0.1 |
| HCs | Any emotion | Accuracy | Hoertnagl et al., 2011 | -0.472 | -0.563, -0.382 | <0.001 | 63.58 | 0.08 | <0.1 |
| HCs | Any emotion | Accuracy | Iakimova et al., 2016 | -0.468 | -0.559, -0.378 | <0.001 | 64.22 | 0.08 | <0.1 |
| HCs | Any emotion | Accuracy | Ulusoy et al., 2020 | -0.456 | -0.543, -0.368 | <0.001 | 61.87 | 0.07 | <0.1 |
| HCs | Any emotion | Accuracy | Jogia et al., 2011 | -0.473 | -0.563, -0.384 | <0.001 | 63.31 | 0.08 | <0.1 |
| HCs | Any emotion | Accuracy | Lahera et al., 2015 | -0.454 | -0.541, -0.367 | <0.001 | 61.24 | 0.07 | <0.1 |
| HCs | Any emotion | Accuracy | Lawlor-Savage et al., 2014 | -0.467 | -0.557, -0.376 | <0.001 | 64.21 | 0.08 | <0.1 |
| HCs | Any emotion | Accuracy | Lee et al., 2020 | -0.465 | -0.555, -0.375 | <0.001 | 64.11 | 0.08 | <0.1 |
| HCs | Any emotion | Accuracy | Lelli-Chiesa et al., 2010 | -0.467 | -0.558, -0.377 | <0.001 | 64.23 | 0.08 | <0.1 |
| HCs | Any emotion | Accuracy | Martino et al., 2011 | -0.467 | -0.557, -0.376 | <0.001 | 64.21 | 0.08 | <0.1 |
| HCs | Any emotion | Accuracy | Navarra-Ventura et al., 2021 | -0.453 | -0.54, -0.366 | <0.001 | 60.77 | 0.07 | <0.1 |
| HCs | Any emotion | Accuracy | Maila de Castro et al., 2015 | -0.456 | -0.544, -0.368 | <0.001 | 62.04 | 0.07 | <0.1 |
| HCs | Any emotion | Accuracy | Nigam et al., 2021 | -0.466 | -0.557, -0.376 | <0.001 | 64.2 | 0.08 | <0.1 |
| HCs | Any emotion | Accuracy | Lescalier et al., 2015 | -0.473 | -0.562, -0.383 | <0.001 | 63.48 | 0.08 | <0.1 |
| HCs | Any emotion | Accuracy | Priyesh et al., 2022 | -0.461 | -0.55, -0.371 | <0.001 | 63.36 | 0.08 | <0.1 |
| HCs | Any emotion | Accuracy | Reddy et al., 2022 | -0.458 | -0.547, -0.37 | <0.001 | 62.81 | 0.07 | <0.1 |
| HCs | Any emotion | Accuracy | Robinson et al., 2015a | -0.472 | -0.562, -0.382 | <0.001 | 63.72 | 0.08 | <0.1 |
| HCs | Any emotion | Accuracy | Robinson et al., 2015b | -0.472 | -0.562, -0.382 | <0.001 | 63.7 | 0.08 | <0.1 |
| HCs | Any emotion | Accuracy | Rowland et al., 2012 | -0.476 | -0.564, -0.388 | <0.001 | 61.99 | 0.07 | <0.1 |
| HCs | Any emotion | Accuracy | Rubin et al., 2022 | -0.476 | -0.565, -0.386 | <0.001 | 61.74 | 0.07 | <0.1 |
| HCs | Any emotion | Accuracy | Ruocco et al., 2014 | -0.47 | -0.562, -0.379 | <0.001 | 62.46 | 0.08 | <0.1 |
| HCs | Any emotion | Accuracy | Sagar et al., 2013 | -0.467 | -0.557, -0.376 | <0.001 | 64.18 | 0.08 | <0.1 |
| HCs | Any emotion | Accuracy | Schaefer et al., 2010 | -0.472 | -0.562, -0.382 | <0.001 | 63.64 | 0.08 | <0.1 |
| HCs | Any emotion | Accuracy | Seidel et al., 2012 | -0.466 | -0.556, -0.376 | <0.001 | 64.17 | 0.08 | <0.1 |
| HCs | Any emotion | Accuracy | Shah et al., 2009 | -0.475 | -0.564, -0.386 | <0.001 | 62.54 | 0.07 | <0.1 |
| HCs | Any emotion | Accuracy | Soeiro-de-Souza et al., 2012a | -0.462 | -0.552, -0.372 | <0.001 | 63.56 | 0.08 | <0.1 |
| HCs | Any emotion | Accuracy | Soeiro-de-Souza et al., 2012b | -0.469 | -0.56, -0.379 | <0.001 | 64.15 | 0.08 | <0.1 |
| HCs | Any emotion | Accuracy | Tesli et al., 2015 | -0.475 | -0.564, -0.385 | <0.001 | 62.63 | 0.07 | <0.1 |
| HCs | Any emotion | Accuracy | Van Rheenen et al., 2014 | -0.469 | -0.56, -0.379 | <0.001 | 64.13 | 0.08 | <0.1 |
| HCs | Any emotion | Accuracy | Vederman et al., 2012 | -0.47 | -0.561, -0.379 | <0.001 | 63.9 | 0.08 | <0.1 |
| HCs | Any emotion | Accuracy | Venn et al., 2004 | -0.469 | -0.559, -0.379 | <0.001 | 64.1 | 0.08 | <0.1 |
| HCs | Any emotion | Accuracy | Versace et al., 2010 | -0.467 | -0.558, -0.377 | <0.001 | 64.22 | 0.08 | <0.1 |
| HCs | Any emotion | Accuracy | Vierck et al., 2015 | -0.469 | -0.559, -0.378 | <0.001 | 64.19 | 0.08 | <0.1 |
| HCs | Any emotion | Accuracy | Wynn et al., 2013 | -0.468 | -0.559, -0.377 | <0.001 | 64.24 | 0.08 | <0.1 |
| HCs | Any emotion | Accuracy | Yalcin-Siedentopf et al., 2014 | -0.464 | -0.555, -0.374 | <0.001 | 64.02 | 0.08 | <0.1 |
| HCs | Any emotion | Accuracy | Zhang et al., 2018 | -0.472 | -0.562, -0.381 | <0.001 | 63.73 | 0.08 | <0.1 |
| HCs | Any emotion | Accuracy | Quidè et al., 2018 | -0.459 | -0.549, -0.37 | <0.001 | 62.93 | 0.08 | <0.1 |
| HCs | Any emotion | Accuracy | Rossell et al., 2014 | -0.469 | -0.56, -0.378 | <0.001 | 64.16 | 0.08 | <0.1 |
| HCs | Any emotion | Accuracy | Van Rheenen et al., 2017 | -0.464 | -0.554, -0.374 | <0.001 | 63.98 | 0.08 | <0.1 |
| HCs | Any emotion | Accuracy | Millet et al., 2023 | -0.471 | -0.562, -0.381 | <0.001 | 63.71 | 0.08 | <0.1 |
| HCs | Any emotion | Accuracy | Miola et al., 2023 | -0.466 | -0.556, -0.375 | <0.001 | 64.18 | 0.08 | <0.1 |
| HCs | Any emotion | Number of errors | Thaler et al., 2013 | 0.295 | -0.045, 0.634 | 0.09 | 0 | 0 | 0.69 |
| HCs | Any emotion | Number of errors | MacPherson et al., 2021 | 0.158 | -0.27, 0.586 | 0.47 | 0 | 0 | 0.95 |
| HCs | Any emotion | Number of errors | Malhi et al., 2007 | 0.267 | -0.028, 0.561 | 0.08 | 0 | 0 | 0.62 |
| HCs | Any emotion | Reaction time | Altamura et al., 2016 | 0.505 | 0.302, 0.709 | <0.001 | 85.23 | 0.2 | <0.1 |
| HCs | Any emotion | Reaction time | Dima et al., 2016 | 0.533 | 0.302, 0.764 | <0.001 | 88.44 | 0.27 | <0.1 |
| HCs | Any emotion | Reaction time | Fernandes et al., 2016 | 0.589 | 0.343, 0.835 | <0.001 | 89.96 | 0.31 | <0.1 |
| HCs | Any emotion | Reaction time | Foland-Ross et al., 2012 | 0.582 | 0.334, 0.83 | <0.001 | 90.16 | 0.32 | <0.1 |
| HCs | Any emotion | Reaction time | Baez et al., 2013 | 0.548 | 0.309, 0.788 | <0.001 | 89.51 | 0.29 | <0.1 |
| HCs | Any emotion | Reaction time | Goghari et al., 2013 | 0.585 | 0.338, 0.832 | <0.001 | 90.1 | 0.32 | <0.1 |
| HCs | Any emotion | Reaction time | Ryan et al., 2013 | 0.584 | 0.333, 0.834 | <0.001 | 89.41 | 0.32 | <0.1 |
| HCs | Any emotion | Reaction time | Kjaerstad et al., 2022 | 0.598 | 0.354, 0.841 | <0.001 | 88.19 | 0.3 | <0.1 |
| HCs | Any emotion | Reaction time | Harmer et al., 2002 | 0.577 | 0.329, 0.825 | <0.001 | 90.21 | 0.32 | <0.1 |
| HCs | Any emotion | Reaction time | Jogia et al., 2011 | 0.568 | 0.319, 0.817 | <0.001 | 90.08 | 0.32 | <0.1 |
| HCs | Any emotion | Reaction time | Lelli-Chiesa et al., 2010 | 0.568 | 0.319, 0.817 | <0.001 | 90.09 | 0.32 | <0.1 |
| HCs | Any emotion | Reaction time | Malhi et al., 2007 | 0.566 | 0.322, 0.81 | <0.001 | 90.06 | 0.31 | <0.1 |
| HCs | Any emotion | Reaction time | Maila de Castro et al., 2015 | 0.574 | 0.326, 0.823 | <0.001 | 90.21 | 0.32 | <0.1 |
| HCs | Any emotion | Reaction time | Priyesh et al., 2022 | 0.596 | 0.354, 0.837 | <0.001 | 89.56 | 0.3 | <0.1 |
| HCs | Any emotion | Reaction time | Reddy et al., 2022 | 0.594 | 0.35, 0.837 | <0.001 | 89.71 | 0.3 | <0.1 |
| HCs | Any emotion | Reaction time | Rubin et al., 2022 | 0.595 | 0.35, 0.84 | <0.001 | 88.89 | 0.31 | <0.1 |
| HCs | Any emotion | Reaction time | Ruocco et al., 2014 | 0.563 | 0.314, 0.813 | <0.001 | 88.47 | 0.32 | <0.1 |
| HCs | Any emotion | Reaction time | Seidel et al., 2012 | 0.467 | 0.305, 0.628 | <0.001 | 75.75 | 0.11 | <0.1 |
| HCs | Any emotion | Reaction time | Shah et al., 2009 | 0.582 | 0.333, 0.831 | <0.001 | 90.12 | 0.32 | <0.1 |
| HCs | Any emotion | Reaction time | Tesli et al., 2015 | 0.581 | 0.33, 0.832 | <0.001 | 89.81 | 0.32 | <0.1 |
| HCs | Any emotion | Reaction time | Vierck et al., 2015 | 0.576 | 0.327, 0.826 | <0.001 | 90.17 | 0.32 | <0.1 |
| HCs | Any emotion | Reaction time | Zhang et al., 2018 | 0.589 | 0.341, 0.837 | <0.001 | 89.84 | 0.32 | <0.1 |
| HCs | Any emotion | Reaction time | Rossell et al., 2014 | 0.591 | 0.344, 0.838 | <0.001 | 89.73 | 0.31 | <0.1 |
| HCs | Any emotion | Reaction time | Van Rheenen et al., 2017 | 0.576 | 0.327, 0.825 | <0.001 | 90.2 | 0.32 | <0.1 |
| HCs | Any emotion | Reaction time | Miola et al., 2023 | 0.587 | 0.339, 0.835 | <0.001 | 89.99 | 0.32 | <0.1 |
| FDRs | Any emotion | Accuracy | Dima et al., 2016 | -0.045 | -0.194, 0.105 | 0.56 | 26.79 | 0.01 | 0.32 |
| FDRs | Any emotion | Accuracy | Fernandes et al., 2016 | -0.031 | -0.179, 0.117 | 0.68 | 26.79 | 0.01 | 0.31 |
| FDRs | Any emotion | Accuracy | Kjaerstad et al., 2022 | -0.086 | -0.229, 0.057 | 0.24 | 12.07 | 0.01 | 0.57 |
| FDRs | Any emotion | Accuracy | Ulusoy et al., 2020 | -0.082 | -0.217, 0.052 | 0.23 | 13.61 | 0.01 | 0.71 |
| FDRs | Any emotion | Accuracy | Jogia et al., 2011 | -0.037 | -0.187, 0.114 | 0.63 | 27.33 | 0.01 | 0.3 |
| FDRs | Any emotion | Accuracy | Lelli-Chiesa et al., 2010 | -0.046 | -0.195, 0.104 | 0.55 | 26.6 | 0.01 | 0.32 |
| FDRs | Any emotion | Accuracy | Nigam et al., 2021 | -0.021 | -0.167, 0.125 | 0.78 | 25.2 | 0.01 | 0.39 |
| FDRs | Any emotion | Accuracy | Reddy et al., 2022 | -0.032 | -0.181, 0.116 | 0.67 | 26.95 | 0.01 | 0.31 |
| FDRs | Any emotion | Accuracy | Ruocco et al., 2014 | 0.032 | -0.111, 0.175 | 0.66 | 0 | 0 | 0.66 |
| FDRs | Any emotion | Accuracy | Seidel et al., 2012 | -0.037 | -0.185, 0.111 | 0.62 | 26.88 | 0.01 | 0.3 |
| FDRs | Any emotion | Accuracy | Vierck et al., 2015 | -0.028 | -0.178, 0.121 | 0.71 | 26.79 | 0.01 | 0.32 |
| FDRs | Any emotion | Reaction time | Dima et al., 2016 | 0.193 | -0.091, 0.477 | 0.18 | 74.89 | 0.11 | <0.1 |
| FDRs | Any emotion | Reaction time | Fernandes et al., 2016 | 0.4 | -0.042, 0.843 | 0.08 | 90.26 | 0.35 | <0.1 |
| FDRs | Any emotion | Reaction time | Kjaerstad et al., 2022 | 0.42 | -0.018, 0.859 | 0.06 | 86.58 | 0.33 | <0.1 |
| FDRs | Any emotion | Reaction time | Jogia et al., 2011 | 0.307 | -0.142, 0.756 | 0.18 | 90.3 | 0.36 | <0.1 |
| FDRs | Any emotion | Reaction time | Lelli-Chiesa et al., 2010 | 0.287 | -0.149, 0.724 | 0.2 | 89.73 | 0.33 | <0.1 |
| FDRs | Any emotion | Reaction time | Reddy et al., 2022 | 0.431 | 0.013, 0.849 | 0.043 | 88.93 | 0.3 | <0.1 |
| FDRs | Any emotion | Reaction time | Ruocco et al., 2014 | 0.343 | -0.126, 0.811 | 0.15 | 86.54 | 0.39 | <0.1 |
| FDRs | Any emotion | Reaction time | Seidel et al., 2012 | 0.38 | -0.072, 0.832 | 0.1 | 90.72 | 0.37 | <0.1 |
| FDRs | Any emotion | Reaction time | Vierck et al., 2015 | 0.386 | -0.066, 0.839 | 0.09 | 90.47 | 0.36 | <0.1 |
| Level II | | | | | | | | | |
| HCs | Negative | Accuracy | Almeida et al., 2010 | -0.321 | -0.432, -0.21 | <0.001 | 67.13 | 0.08 | <0.1 |
| HCs | Negative | Accuracy | Bjertrup et al., 2021 | -0.313 | -0.422, -0.204 | <0.001 | 66.3 | 0.08 | <0.1 |
| HCs | Negative | Accuracy | Branco et al., 2017 | -0.321 | -0.432, -0.209 | <0.001 | 67.12 | 0.08 | <0.1 |
| HCs | Negative | Accuracy | Darke et al., 2021 | -0.3 | -0.405, -0.195 | <0.001 | 63.36 | 0.07 | <0.1 |
| HCs | Negative | Accuracy | Dima et al., 2016 | -0.314 | -0.425, -0.204 | <0.001 | 66.43 | 0.08 | <0.1 |
| HCs | Negative | Accuracy | Fernandes et al., 2016 | -0.318 | -0.429, -0.207 | <0.001 | 66.95 | 0.08 | <0.1 |
| HCs | Negative | Accuracy | Golkhatmi et al., 2013 | -0.312 | -0.422, -0.202 | <0.001 | 66.14 | 0.08 | <0.1 |
| HCs | Negative | Accuracy | Baez et al., 2013 | -0.311 | -0.42, -0.202 | <0.001 | 65.94 | 0.08 | <0.1 |
| HCs | Negative | Accuracy | Derntl et al., 2012 | -0.323 | -0.434, -0.212 | <0.001 | 67.19 | 0.08 | <0.1 |
| HCs | Negative | Accuracy | Goghari et al., 2013 | -0.325 | -0.436, -0.214 | <0.001 | 67.14 | 0.08 | <0.1 |
| HCs | Negative | Accuracy | Pan et al., 2013 | -0.317 | -0.428, -0.206 | <0.001 | 66.78 | 0.08 | <0.1 |
| HCs | Negative | Accuracy | Harmer et al., 2002 | -0.331 | -0.44, -0.221 | <0.001 | 66.44 | 0.08 | <0.1 |
| HCs | Negative | Accuracy | Hoertnagl et al., 2011 | -0.319 | -0.431, -0.208 | <0.001 | 66.99 | 0.08 | <0.1 |
| HCs | Negative | Accuracy | Iakimova et al., 2016 | -0.321 | -0.432, -0.21 | <0.001 | 67.14 | 0.08 | <0.1 |
| HCs | Negative | Accuracy | Ulusoy et al., 2020 | -0.316 | -0.426, -0.205 | <0.001 | 66.68 | 0.08 | <0.1 |
| HCs | Negative | Accuracy | Jogia et al., 2011 | -0.328 | -0.44, -0.217 | <0.001 | 66.85 | 0.08 | <0.1 |
| HCs | Negative | Accuracy | Lawlor-Savage et al., 2014 | -0.321 | -0.433, -0.21 | <0.001 | 67.16 | 0.08 | <0.1 |
| HCs | Negative | Accuracy | Lelli-Chiesa et al., 2010 | -0.319 | -0.43, -0.208 | <0.001 | 66.97 | 0.08 | <0.1 |
| HCs | Negative | Accuracy | Martino et al., 2011 | -0.322 | -0.433, -0.21 | <0.001 | 67.1 | 0.09 | <0.1 |
| HCs | Negative | Accuracy | Maila de Castro et al., 2015 | -0.31 | -0.419, -0.202 | <0.001 | 65.83 | 0.08 | <0.1 |
| HCs | Negative | Accuracy | Nigam et al., 2021 | -0.318 | -0.429, -0.207 | <0.001 | 66.96 | 0.08 | <0.1 |
| HCs | Negative | Accuracy | Lescalier et al., 2015 | -0.328 | -0.439, -0.218 | <0.001 | 66.86 | 0.08 | <0.1 |
| HCs | Negative | Accuracy | Priyesh et al., 2022 | -0.306 | -0.413, -0.198 | <0.001 | 64.81 | 0.08 | <0.1 |
| HCs | Negative | Accuracy | Reddy et al., 2022 | -0.305 | -0.413, -0.198 | <0.001 | 64.74 | 0.08 | <0.1 |
| HCs | Negative | Accuracy | Robinson et al., 2015a | -0.325 | -0.436, -0.214 | <0.001 | 67.16 | 0.08 | <0.1 |
| HCs | Negative | Accuracy | Robinson et al., 2015b | -0.326 | -0.438, -0.214 | <0.001 | 67.04 | 0.09 | <0.1 |
| HCs | Negative | Accuracy | Rowland et al., 2012 | -0.332 | -0.442, -0.222 | <0.001 | 66.16 | 0.08 | <0.1 |
| HCs | Negative | Accuracy | Rubin et al., 2022 | -0.331 | -0.411, -0.25 | <0.001 | 33.37 | 0.02 | <0.1 |
| HCs | Negative | Accuracy | Ruocco et al., 2014 | -0.33 | -0.442, -0.218 | <0.001 | 64.47 | 0.09 | <0.1 |
| HCs | Negative | Accuracy | Sagar et al., 2013 | -0.317 | -0.428, -0.207 | <0.001 | 66.86 | 0.08 | <0.1 |
| HCs | Negative | Accuracy | Schaefer et al., 2010 | -0.328 | -0.439, -0.217 | <0.001 | 66.91 | 0.08 | <0.1 |
| HCs | Negative | Accuracy | Shah et al., 2009 | -0.33 | -0.441, -0.22 | <0.001 | 66.59 | 0.08 | <0.1 |
| HCs | Negative | Accuracy | Soeiro-de-Souza et al., 2012a | -0.32 | -0.432, -0.208 | <0.001 | 66.89 | 0.09 | <0.1 |
| HCs | Negative | Accuracy | Soeiro-de-Souza et al., 2012b | -0.325 | -0.436, -0.213 | <0.001 | 67.15 | 0.09 | <0.1 |
| HCs | Negative | Accuracy | Tesli et al., 2015 | -0.328 | -0.44, -0.216 | <0.001 | 66.53 | 0.09 | <0.1 |
| HCs | Negative | Accuracy | Van Rheenen et al., 2014 | -0.322 | -0.434, -0.21 | <0.001 | 67.1 | 0.09 | <0.1 |
| HCs | Negative | Accuracy | Vederman et al., 2012 | -0.331 | -0.442, -0.22 | <0.001 | 66.09 | 0.08 | <0.1 |
| HCs | Negative | Accuracy | Venn et al., 2004 | -0.323 | -0.434, -0.213 | <0.001 | 67.15 | 0.08 | <0.1 |
| HCs | Negative | Accuracy | Versace et al., 2010 | -0.323 | -0.434, -0.212 | <0.001 | 67.19 | 0.08 | <0.1 |
| HCs | Negative | Accuracy | Vierck et al., 2015 | -0.318 | -0.429, -0.207 | <0.001 | 66.9 | 0.08 | <0.1 |
| HCs | Negative | Accuracy | Yalcin-Siedentopf et al., 2014 | -0.321 | -0.433, -0.209 | <0.001 | 67.06 | 0.09 | <0.1 |
| HCs | Negative | Accuracy | Zhang et al., 2018 | -0.324 | -0.436, -0.212 | <0.001 | 67.08 | 0.09 | <0.1 |
| HCs | Negative | Accuracy | de Siqueria Rotenberg et al., 2023 | -0.327 | -0.44, -0.215 | <0.001 | 65.43 | 0.09 | <0.1 |
| HCs | Negative | Accuracy | Millet et al., 2023 | -0.324 | -0.437, -0.212 | <0.001 | 66.88 | 0.09 | <0.1 |
| HCs | Negative | Accuracy | Miola et al., 2023 | -0.323 | -0.435, -0.212 | <0.001 | 67.16 | 0.09 | <0.1 |
| HCs | Negative | Number of errors | Thaler et al., 2013 | 0.163 | -0.176, 0.501 | 0.35 | 0 | 0 | 0.75 |
| HCs | Negative | Number of errors | MacPherson et al., 2021 | 0.165 | -0.263, 0.593 | 0.45 | 0 | 0 | 0.73 |
| HCs | Negative | Number of errors | Malhi et al., 2007 | 0.194 | -0.1, 0.488 | 0.2 | 0 | 0 | 0.94 |
| HCs | Negative | Reaction time | Altamura et al., 2016 | 0.345 | 0.105, 0.585 | 0.005 | 86.69 | 0.21 | <0.1 |
| HCs | Negative | Reaction time | Dima et al., 2016 | 0.373 | 0.104, 0.641 | 0.006 | 89.05 | 0.27 | <0.1 |
| HCs | Negative | Reaction time | Fernandes et al., 2016 | 0.445 | 0.155, 0.735 | 0.003 | 90.88 | 0.33 | <0.1 |
| HCs | Negative | Reaction time | Baez et al., 2013 | 0.391 | 0.113, 0.669 | 0.006 | 90.12 | 0.3 | <0.1 |
| HCs | Negative | Reaction time | Goghari et al., 2013 | 0.439 | 0.148, 0.729 | 0.003 | 90.96 | 0.33 | <0.1 |
| HCs | Negative | Reaction time | Kjaerstad et al., 2022 | 0.458 | 0.169, 0.747 | 0.002 | 88.95 | 0.32 | <0.1 |
| HCs | Negative | Reaction time | Harmer et al., 2002 | 0.439 | 0.149, 0.73 | 0.003 | 90.96 | 0.33 | <0.1 |
| HCs | Negative | Reaction time | Jogia et al., 2011 | 0.414 | 0.124, 0.704 | 0.005 | 90.69 | 0.33 | <0.1 |
| HCs | Negative | Reaction time | Lelli-Chiesa et al., 2010 | 0.415 | 0.124, 0.705 | 0.005 | 90.7 | 0.33 | <0.1 |
| HCs | Negative | Reaction time | Malhi et al., 2007 | 0.426 | 0.138, 0.713 | 0.004 | 90.95 | 0.33 | <0.1 |
| HCs | Negative | Reaction time | Priyesh et al., 2022 | 0.46 | 0.175, 0.745 | 0.002 | 90.48 | 0.32 | <0.1 |
| HCs | Negative | Reaction time | Reddy et al., 2022 | 0.451 | 0.163, 0.74 | 0.002 | 90.72 | 0.33 | <0.1 |
| HCs | Negative | Reaction time | Rubin et al., 2022 | 0.5 | 0.277, 0.723 | <0.001 | 81.87 | 0.17 | <0.1 |
| HCs | Negative | Reaction time | Ruocco et al., 2014 | 0.427 | 0.132, 0.722 | 0.005 | 88.91 | 0.34 | <0.1 |
| HCs | Negative | Reaction time | Shah et al., 2009 | 0.432 | 0.14, 0.725 | 0.004 | 90.9 | 0.34 | <0.1 |
| HCs | Negative | Reaction time | Tesli et al., 2015 | 0.425 | 0.132, 0.719 | 0.005 | 90.43 | 0.34 | <0.1 |
| HCs | Negative | Reaction time | Vierck et al., 2015 | 0.424 | 0.132, 0.716 | 0.004 | 90.86 | 0.34 | <0.1 |
| HCs | Negative | Reaction time | Zhang et al., 2018 | 0.437 | 0.143, 0.73 | 0.004 | 90.75 | 0.34 | <0.1 |
| HCs | Negative | Reaction time | Miola et al., 2023 | 0.443 | 0.151, 0.735 | 0.003 | 90.79 | 0.33 | <0.1 |
| HCs | Positive | Accuracy | Almeida et al., 2010 | -0.267 | -0.379, -0.155 | <0.001 | 63.55 | 0.07 | <0.1 |
| HCs | Positive | Accuracy | Bjertrup et al., 2021 | -0.29 | -0.399, -0.182 | <0.001 | 61.39 | 0.06 | <0.1 |
| HCs | Positive | Accuracy | Fernandes et al., 2016 | -0.275 | -0.388, -0.161 | <0.001 | 64.34 | 0.07 | <0.1 |
| HCs | Positive | Accuracy | Golkhatmi et al., 2013 | -0.267 | -0.38, -0.155 | <0.001 | 63.61 | 0.07 | <0.1 |
| HCs | Positive | Accuracy | Baez et al., 2013 | -0.268 | -0.38, -0.156 | <0.001 | 63.69 | 0.07 | <0.1 |
| HCs | Positive | Accuracy | Derntl et al., 2012 | -0.275 | -0.389, -0.162 | <0.001 | 64.34 | 0.07 | <0.1 |
| HCs | Positive | Accuracy | Goghari et al., 2013 | -0.288 | -0.398, -0.178 | <0.001 | 62.31 | 0.07 | <0.1 |
| HCs | Positive | Accuracy | Pan et al., 2013 | -0.283 | -0.396, -0.169 | <0.001 | 63.68 | 0.07 | <0.1 |
| HCs | Positive | Accuracy | Summers et al., 2006 | -0.276 | -0.389, -0.162 | <0.001 | 64.36 | 0.07 | <0.1 |
| HCs | Positive | Accuracy | Harmer et al., 2002 | -0.278 | -0.391, -0.165 | <0.001 | 64.19 | 0.07 | <0.1 |
| HCs | Positive | Accuracy | Hoertnagl et al., 2011 | -0.27 | -0.384, -0.156 | <0.001 | 64.06 | 0.07 | <0.1 |
| HCs | Positive | Accuracy | Iakimova et al., 2016 | -0.275 | -0.389, -0.161 | <0.001 | 64.36 | 0.07 | <0.1 |
| HCs | Positive | Accuracy | Ulusoy et al., 2020 | -0.271 | -0.384, -0.157 | <0.001 | 64.15 | 0.07 | <0.1 |
| HCs | Positive | Accuracy | Lawlor-Savage et al., 2014 | -0.261 | -0.371, -0.151 | <0.001 | 62.05 | 0.07 | <0.1 |
| HCs | Positive | Accuracy | Martino et al., 2011 | -0.281 | -0.395, -0.167 | <0.001 | 63.94 | 0.07 | <0.1 |
| HCs | Positive | Accuracy | Maila de Castro et al., 2015 | -0.268 | -0.381, -0.156 | <0.001 | 63.78 | 0.07 | <0.1 |
| HCs | Positive | Accuracy | Nigam et al., 2021 | -0.267 | -0.379, -0.154 | <0.001 | 63.53 | 0.07 | <0.1 |
| HCs | Positive | Accuracy | Lescalier et al., 2015 | -0.285 | -0.397, -0.172 | <0.001 | 63.31 | 0.07 | <0.1 |
| HCs | Positive | Accuracy | Priyesh et al., 2022 | -0.259 | -0.369, -0.15 | <0.001 | 61.37 | 0.06 | <0.1 |
| HCs | Positive | Accuracy | Robinson et al., 2015a | -0.288 | -0.399, -0.178 | <0.001 | 62.27 | 0.07 | <0.1 |
| HCs | Positive | Accuracy | Robinson et al., 2015b | -0.28 | -0.394, -0.166 | <0.001 | 64.08 | 0.07 | <0.1 |
| HCs | Positive | Accuracy | Rowland et al., 2012 | -0.279 | -0.393, -0.165 | <0.001 | 64.2 | 0.07 | <0.1 |
| HCs | Positive | Accuracy | Rubin et al., 2022 | -0.229 | -0.313, -0.146 | <0.001 | 30.05 | 0.02 | <0.1 |
| HCs | Positive | Accuracy | Ruocco et al., 2014 | -0.284 | -0.398, -0.169 | <0.001 | 61.02 | 0.07 | <0.1 |
| HCs | Positive | Accuracy | Sagar et al., 2013 | -0.276 | -0.389, -0.162 | <0.001 | 64.31 | 0.07 | <0.1 |
| HCs | Positive | Accuracy | Schaefer et al., 2010 | -0.28 | -0.393, -0.167 | <0.001 | 64.03 | 0.07 | <0.1 |
| HCs | Positive | Accuracy | Shah et al., 2009 | -0.283 | -0.396, -0.17 | <0.001 | 63.59 | 0.07 | <0.1 |
| HCs | Positive | Accuracy | Soeiro-de-Souza et al., 2012a | -0.275 | -0.39, -0.16 | <0.001 | 64.19 | 0.07 | <0.1 |
| HCs | Positive | Accuracy | Soeiro-de-Souza et al., 2012b | -0.282 | -0.396, -0.169 | <0.001 | 63.74 | 0.07 | <0.1 |
| HCs | Positive | Accuracy | Tesli et al., 2015 | -0.287 | -0.399, -0.174 | <0.001 | 62.41 | 0.07 | <0.1 |
| HCs | Positive | Accuracy | Van Rheenen et al., 2014 | -0.276 | -0.39, -0.161 | <0.001 | 64.3 | 0.07 | <0.1 |
| HCs | Positive | Accuracy | Vederman et al., 2012 | -0.285 | -0.398, -0.172 | <0.001 | 62.94 | 0.07 | <0.1 |
| HCs | Positive | Accuracy | Venn et al., 2004 | -0.273 | -0.386, -0.161 | <0.001 | 64.25 | 0.07 | <0.1 |
| HCs | Positive | Accuracy | Versace et al., 2010 | -0.267 | -0.379, -0.154 | <0.001 | 63.55 | 0.07 | <0.1 |
| HCs | Positive | Accuracy | Vierck et al., 2015 | -0.27 | -0.384, -0.157 | <0.001 | 64.07 | 0.07 | <0.1 |
| HCs | Positive | Accuracy | Yalcin-Siedentopf et al., 2014 | -0.272 | -0.386, -0.157 | <0.001 | 64.16 | 0.07 | <0.1 |
| HCs | Positive | Accuracy | Zhang et al., 2018 | -0.276 | -0.391, -0.162 | <0.001 | 64.26 | 0.07 | <0.1 |
| HCs | Positive | Accuracy | de Siqueria Rotenberg et al., 2023 | -0.273 | -0.389, -0.157 | <0.001 | 62.59 | 0.07 | <0.1 |
| HCs | Positive | Accuracy | Millet et al., 2023 | -0.278 | -0.393, -0.163 | <0.001 | 63.98 | 0.07 | <0.1 |
| HCs | Positive | Reaction time | Altamura et al., 2016 | 0.533 | 0.288, 0.778 | <0.001 | 83.29 | 0.14 | <0.1 |
| HCs | Positive | Reaction time | Fernandes et al., 2016 | 0.594 | 0.29, 0.898 | <0.001 | 89.2 | 0.23 | <0.1 |
| HCs | Positive | Reaction time | Baez et al., 2013 | 0.588 | 0.289, 0.887 | <0.001 | 88.93 | 0.22 | <0.1 |
| HCs | Positive | Reaction time | Goghari et al., 2013 | 0.648 | 0.339, 0.956 | <0.001 | 89.57 | 0.24 | <0.1 |
| HCs | Positive | Reaction time | Kjaerstad et al., 2022 | 0.674 | 0.375, 0.973 | <0.001 | 85.53 | 0.22 | <0.1 |
| HCs | Positive | Reaction time | Harmer et al., 2002 | 0.622 | 0.311, 0.933 | <0.001 | 89.82 | 0.25 | <0.1 |
| HCs | Positive | Reaction time | Priyesh et al., 2022 | 0.668 | 0.371, 0.965 | <0.001 | 88.53 | 0.22 | <0.1 |
| HCs | Positive | Reaction time | Rubin et al., 2022 | 0.567 | 0.274, 0.859 | <0.001 | 86.16 | 0.2 | <0.1 |
| HCs | Positive | Reaction time | Ruocco et al., 2014 | 0.601 | 0.286, 0.917 | <0.001 | 86.56 | 0.25 | <0.1 |
| HCs | Positive | Reaction time | Shah et al., 2009 | 0.648 | 0.336, 0.96 | <0.001 | 89.49 | 0.24 | <0.1 |
| HCs | Positive | Reaction time | Tesli et al., 2015 | 0.652 | 0.338, 0.966 | <0.001 | 88.72 | 0.25 | <0.1 |
| HCs | Positive | Reaction time | Vierck et al., 2015 | 0.625 | 0.309, 0.94 | <0.001 | 89.76 | 0.25 | <0.1 |
| HCs | Positive | Reaction time | Zhang et al., 2018 | 0.678 | 0.389, 0.968 | <0.001 | 87.2 | 0.2 | <0.1 |
| FDRs | Negative | Accuracy | Dima et al., 2016 | -0.03 | -0.145, 0.086 | 0.62 | 0.95 | 0 | 0.56 |
| FDRs | Negative | Accuracy | Fernandes et al., 2016 | -0.017 | -0.132, 0.097 | 0.77 | 1 | 0 | 0.61 |
| FDRs | Negative | Accuracy | Kjaerstad et al., 2022 | -0.062 | -0.185, 0.062 | 0.33 | 0 | 0 | 0.73 |
| FDRs | Negative | Accuracy | Ulusoy et al., 2020 | -0.052 | -0.168, 0.064 | 0.38 | 0 | 0 | 0.83 |
| FDRs | Negative | Accuracy | Jogia et al., 2011 | -0.024 | -0.141, 0.093 | 0.69 | 1.86 | 0 | 0.55 |
| FDRs | Negative | Accuracy | Lelli-Chiesa et al., 2010 | -0.031 | -0.146, 0.085 | 0.6 | 0.68 | 0 | 0.56 |
| FDRs | Negative | Accuracy | Nigam et al., 2021 | -0.013 | -0.127, 0.101 | 0.82 | 0.46 | 0 | 0.7 |
| FDRs | Negative | Accuracy | Reddy et al., 2022 | -0.021 | -0.136, 0.095 | 0.72 | 1.35 | 0 | 0.57 |
| FDRs | Negative | Accuracy | Ruocco et al., 2014 | 0.017 | -0.13, 0.165 | 0.82 | 0 | 0 | 0.64 |
| FDRs | Negative | Accuracy | Vierck et al., 2015 | -0.017 | -0.133, 0.1 | 0.78 | 1.56 | 0 | 0.6 |
| FDRs | Negative | Reaction time | Dima et al., 2016 | 0.19 | -0.128, 0.509 | 0.24 | 79.25 | 0.13 | <0.1 |
| FDRs | Negative | Reaction time | Fernandes et al., 2016 | 0.433 | -0.07, 0.935 | 0.09 | 92.11 | 0.4 | <0.1 |
| FDRs | Negative | Reaction time | Kjaerstad et al., 2022 | 0.452 | -0.046, 0.951 | 0.07 | 88.74 | 0.39 | <0.1 |
| FDRs | Negative | Reaction time | Jogia et al., 2011 | 0.324 | -0.191, 0.839 | 0.22 | 92.28 | 0.42 | <0.1 |
| FDRs | Negative | Reaction time | Lelli-Chiesa et al., 2010 | 0.301 | -0.199, 0.802 | 0.24 | 91.84 | 0.4 | <0.1 |
| FDRs | Negative | Reaction time | Reddy et al., 2022 | 0.464 | -0.01, 0.938 | 0.06 | 91.02 | 0.35 | <0.1 |
| FDRs | Negative | Reaction time | Ruocco et al., 2014 | 0.373 | -0.163, 0.91 | 0.17 | 88.81 | 0.46 | <0.1 |
| FDRs | Negative | Reaction time | Vierck et al., 2015 | 0.415 | -0.1, 0.931 | 0.11 | 92.32 | 0.43 | <0.1 |
| FDRs | Positive | Accuracy | Fernandes et al., 2016 | -0.063 | -0.189, 0.064 | 0.33 | 0 | 0 | 0.48 |
| FDRs | Positive | Accuracy | Kjaerstad et al., 2022 | -0.101 | -0.243, 0.04 | 0.16 | 0 | 0 | 0.37 |
| FDRs | Positive | Accuracy | Ulusoy et al., 2020 | -0.107 | -0.237, 0.023 | 0.11 | 0 | 0 | 0.63 |
| FDRs | Positive | Accuracy | Nigam et al., 2021 | -0.063 | -0.189, 0.064 | 0.33 | 0.02 | 0 | 0.47 |
| FDRs | Positive | Accuracy | Ruocco et al., 2014 | -0.051 | -0.231, 0.129 | 0.58 | 0 | 0 | 0.34 |
| FDRs | Positive | Accuracy | Vierck et al., 2015 | -0.073 | -0.201, 0.054 | 0.26 | 0.03 | 0 | 0.33 |
| FDRs | Positive | Reaction time | Fernandes et al., 2016 | 0.249 | -0.1, 0.598 | 0.16 | 78.12 | 0.07 | <0.1 |
| FDRs | Positive | Reaction time | Kjaerstad et al., 2022 | 0.464 | 0.306, 0.622 | <0.001 | 0 | 0 | 0.64 |
| FDRs | Positive | Reaction time | Ruocco et al., 2014 | 0.095 | -0.16, 0.35 | 0.47 | 16.91 | 0.01 | 0.37 |
| FDRs | Positive | Reaction time | Vierck et al., 2015 | 0.27 | -0.092, 0.633 | 0.14 | 78.36 | 0.07 | <0.1 |
| Level III | | | | | | | | | |
| HCs | Anger | Accuracy | Bjertrup et al., 2021 | -0.253 | -0.336, -0.17 | <0.001 | 25.19 | 0.01 | 0.2 |
| HCs | Anger | Accuracy | Branco et al., 2017 | -0.26 | -0.345, -0.174 | <0.001 | 27.57 | 0.01 | 0.12 |
| HCs | Anger | Accuracy | Fernandes et al., 2016 | -0.265 | -0.35, -0.18 | <0.001 | 27.93 | 0.01 | 0.12 |
| HCs | Anger | Accuracy | Golkhatmi et al., 2013 | -0.262 | -0.347, -0.176 | <0.001 | 27.86 | 0.01 | 0.12 |
| HCs | Anger | Accuracy | Baez et al., 2013 | -0.259 | -0.343, -0.174 | <0.001 | 27.08 | 0.01 | 0.13 |
| HCs | Anger | Accuracy | Derntl et al., 2012 | -0.26 | -0.345, -0.175 | <0.001 | 27.39 | 0.01 | 0.12 |
| HCs | Anger | Accuracy | Goghari et al., 2013 | -0.262 | -0.347, -0.177 | <0.001 | 27.73 | 0.01 | 0.12 |
| HCs | Anger | Accuracy | Pan et al., 2013 | -0.24 | -0.32, -0.159 | <0.001 | 19.53 | 0.01 | 0.3 |
| HCs | Anger | Accuracy | Kjaerstad et al., 2022 | -0.274 | -0.352, -0.196 | <0.001 | 10.09 | 0 | 0.39 |
| HCs | Anger | Accuracy | Harmer et al., 2002 | -0.268 | -0.353, -0.183 | <0.001 | 27.44 | 0.01 | 0.15 |
| HCs | Anger | Accuracy | Hoertnagl et al., 2011 | -0.267 | -0.353, -0.181 | <0.001 | 28.24 | 0.01 | 0.13 |
| HCs | Anger | Accuracy | Iakimova et al., 2016 | -0.244 | -0.325, -0.163 | <0.001 | 21.62 | 0.01 | 0.27 |
| HCs | Anger | Accuracy | Ulusoy et al., 2020 | -0.244 | -0.325, -0.163 | <0.001 | 21.7 | 0.01 | 0.26 |
| HCs | Anger | Accuracy | Lawlor-Savage et al., 2014 | -0.261 | -0.346, -0.176 | <0.001 | 27.68 | 0.01 | 0.12 |
| HCs | Anger | Accuracy | Martino et al., 2011 | -0.267 | -0.354, -0.181 | <0.001 | 28.25 | 0.01 | 0.13 |
| HCs | Anger | Accuracy | Maila de Castro et al., 2015 | -0.252 | -0.335, -0.169 | <0.001 | 24.98 | 0.01 | 0.18 |
| HCs | Anger | Accuracy | Nigam et al., 2021 | -0.264 | -0.35, -0.179 | <0.001 | 28 | 0.01 | 0.12 |
| HCs | Anger | Accuracy | Robinson et al., 2015a | -0.258 | -0.343, -0.173 | <0.001 | 27.17 | 0.01 | 0.13 |
| HCs | Anger | Accuracy | Robinson et al., 2015b | -0.26 | -0.346, -0.174 | <0.001 | 27.68 | 0.01 | 0.13 |
| HCs | Anger | Accuracy | Rubin et al., 2022 | -0.276 | -0.361, -0.191 | <0.001 | 23.04 | 0.01 | 0.21 |
| HCs | Anger | Accuracy | Ruocco et al., 2014 | -0.268 | -0.359, -0.178 | <0.001 | 27.98 | 0.02 | 0.12 |
| HCs | Anger | Accuracy | Schaefer et al., 2010 | -0.266 | -0.351, -0.181 | <0.001 | 27.82 | 0.01 | 0.13 |
| HCs | Anger | Accuracy | Soeiro-de-Souza et al., 2012a | -0.26 | -0.346, -0.173 | <0.001 | 27.8 | 0.01 | 0.13 |
| HCs | Anger | Accuracy | Soeiro-de-Souza et al., 2012b | -0.261 | -0.347, -0.175 | <0.001 | 27.81 | 0.01 | 0.12 |
| HCs | Anger | Accuracy | Van Rheenen et al., 2014 | -0.261 | -0.348, -0.175 | <0.001 | 28.06 | 0.01 | 0.12 |
| HCs | Anger | Accuracy | Vederman et al., 2012 | -0.274 | -0.359, -0.188 | <0.001 | 25.45 | 0.01 | 0.18 |
| HCs | Anger | Accuracy | Venn et al., 2004 | -0.261 | -0.345, -0.176 | <0.001 | 27.41 | 0.01 | 0.12 |
| HCs | Anger | Accuracy | Vierck et al., 2015 | -0.256 | -0.341, -0.171 | <0.001 | 26.71 | 0.01 | 0.14 |
| HCs | Anger | Accuracy | Yalcin-Siedentopf et al., 2014 | -0.267 | -0.353, -0.18 | <0.001 | 28.39 | 0.02 | 0.12 |
| HCs | Anger | Accuracy | Zhang et al., 2018 | -0.261 | -0.348, -0.175 | <0.001 | 28.11 | 0.01 | 0.12 |
| HCs | Anger | Accuracy | Millet et al., 2023 | -0.264 | -0.352, -0.177 | <0.001 | 28.65 | 0.02 | 0.12 |
| HCs | Anger | Accuracy | Miola et al., 2023 | -0.252 | -0.336, -0.168 | <0.001 | 25.29 | 0.01 | 0.16 |
| HCs | Anger | Reaction time | Altamura et al., 2016 | 0.308 | 0.153, 0.464 | <0.001 | 50.33 | 0.03 | <0.1 |
| HCs | Anger | Reaction time | Fernandes et al., 2016 | 0.495 | 0.171, 0.82 | 0.003 | 89.13 | 0.22 | <0.1 |
| HCs | Anger | Reaction time | Baez et al., 2013 | 0.401 | 0.134, 0.667 | 0.003 | 83.64 | 0.13 | <0.1 |
| HCs | Anger | Reaction time | Goghari et al., 2013 | 0.504 | 0.188, 0.82 | 0.002 | 88.62 | 0.2 | <0.1 |
| HCs | Anger | Reaction time | Kjaerstad et al., 2022 | 0.522 | 0.219, 0.825 | 0.001 | 82.94 | 0.17 | <0.1 |
| HCs | Anger | Reaction time | Harmer et al., 2002 | 0.467 | 0.145, 0.788 | 0.004 | 89.11 | 0.21 | <0.1 |
| HCs | Anger | Reaction time | Rubin et al., 2022 | 0.51 | 0.182, 0.838 | 0.002 | 86.86 | 0.22 | <0.1 |
| HCs | Anger | Reaction time | Ruocco et al., 2014 | 0.483 | 0.143, 0.823 | 0.005 | 85.99 | 0.24 | <0.1 |
| HCs | Anger | Reaction time | Vierck et al., 2015 | 0.483 | 0.152, 0.814 | 0.004 | 89.32 | 0.23 | <0.1 |
| HCs | Anger | Reaction time | Zhang et al., 2018 | 0.489 | 0.155, 0.823 | 0.004 | 89.12 | 0.23 | <0.1 |
| HCs | Anger | Reaction time | Miola et al., 2023 | 0.495 | 0.165, 0.825 | 0.003 | 89.13 | 0.22 | <0.1 |
| HCs | Disgust | Accuracy | Bjertrup et al., 2021 | -0.318 | -0.45, -0.185 | <0.001 | 52.84 | 0.05 | <0.1 |
| HCs | Disgust | Accuracy | Branco et al., 2017 | -0.318 | -0.454, -0.181 | <0.001 | 53.71 | 0.05 | <0.1 |
| HCs | Disgust | Accuracy | Golkhatmi et al., 2013 | -0.315 | -0.45, -0.181 | <0.001 | 52.91 | 0.05 | <0.1 |
| HCs | Disgust | Accuracy | Baez et al., 2013 | -0.311 | -0.442, -0.18 | <0.001 | 50.93 | 0.04 | <0.1 |
| HCs | Disgust | Accuracy | Derntl et al., 2012 | -0.343 | -0.475, -0.211 | <0.001 | 51.26 | 0.04 | <0.1 |
| HCs | Disgust | Accuracy | Kjaerstad et al., 2022 | -0.345 | -0.483, -0.206 | <0.001 | 49.21 | 0.05 | <0.1 |
| HCs | Disgust | Accuracy | Harmer et al., 2002 | -0.351 | -0.472, -0.23 | <0.001 | 42.98 | 0.03 | <0.1 |
| HCs | Disgust | Accuracy | Hoertnagl et al., 2011 | -0.298 | -0.421, -0.175 | <0.001 | 43.22 | 0.03 | <0.1 |
| HCs | Disgust | Accuracy | Iakimova et al., 2016 | -0.32 | -0.456, -0.184 | <0.001 | 54.18 | 0.05 | <0.1 |
| HCs | Disgust | Accuracy | Martino et al., 2011 | -0.314 | -0.45, -0.178 | <0.001 | 52.75 | 0.05 | <0.1 |
| HCs | Disgust | Accuracy | Nigam et al., 2021 | -0.327 | -0.464, -0.19 | <0.001 | 54.94 | 0.05 | <0.1 |
| HCs | Disgust | Accuracy | Robinson et al., 2015a | -0.326 | -0.464, -0.188 | <0.001 | 55 | 0.05 | <0.1 |
| HCs | Disgust | Accuracy | Robinson et al., 2015b | -0.338 | -0.475, -0.2 | <0.001 | 53.88 | 0.05 | <0.1 |
| HCs | Disgust | Accuracy | Rubin et al., 2022 | -0.341 | -0.481, -0.201 | <0.001 | 51.75 | 0.05 | <0.1 |
| HCs | Disgust | Accuracy | Schaefer et al., 2010 | -0.339 | -0.473, -0.205 | <0.001 | 52.93 | 0.05 | <0.1 |
| HCs | Disgust | Accuracy | Soeiro-de-Souza et al., 2012a | -0.341 | -0.478, -0.204 | <0.001 | 52.71 | 0.05 | <0.1 |
| HCs | Disgust | Accuracy | Soeiro-de-Souza et al., 2012b | -0.335 | -0.473, -0.198 | <0.001 | 54.48 | 0.05 | <0.1 |
| HCs | Disgust | Accuracy | Venn et al., 2004 | -0.336 | -0.47, -0.202 | <0.001 | 53.54 | 0.05 | <0.1 |
| HCs | Disgust | Accuracy | Vierck et al., 2015 | -0.307 | -0.437, -0.177 | <0.001 | 49.23 | 0.04 | <0.1 |
| HCs | Disgust | Accuracy | Yalcin-Siedentopf et al., 2014 | -0.304 | -0.432, -0.175 | <0.001 | 47.43 | 0.04 | <0.1 |
| HCs | Disgust | Accuracy | Millet et al., 2023 | -0.323 | -0.464, -0.183 | <0.001 | 54.74 | 0.05 | <0.1 |
| HCs | Disgust | Accuracy | Miola et al., 2023 | -0.334 | -0.472, -0.196 | <0.001 | 54.69 | 0.05 | <0.1 |
| HCs | Disgust | Reaction time | Baez et al., 2013 | 0.131 | -0.12, 0.382 | 0.31 | 63.15 | 0.05 | <0.1 |
| HCs | Disgust | Reaction time | Kjaerstad et al., 2022 | 0.355 | -0.042, 0.752 | 0.08 | 75.38 | 0.17 | <0.1 |
| HCs | Disgust | Reaction time | Harmer et al., 2002 | 0.339 | -0.04, 0.719 | 0.08 | 84.25 | 0.17 | <0.1 |
| HCs | Disgust | Reaction time | Malhi et al., 2007 | 0.25 | -0.117, 0.618 | 0.18 | 84.4 | 0.16 | <0.1 |
| HCs | Disgust | Reaction time | Rubin et al., 2022 | 0.354 | -0.041, 0.75 | 0.08 | 76.66 | 0.17 | <0.1 |
| HCs | Disgust | Reaction time | Vierck et al., 2015 | 0.19 | -0.15, 0.53 | 0.27 | 78.34 | 0.12 | <0.1 |
| HCs | Disgust | Reaction time | Miola et al., 2023 | 0.318 | -0.092, 0.727 | 0.13 | 85.04 | 0.19 | <0.1 |
| HCs | Fear | Accuracy | Almeida et al., 2010 | -0.367 | -0.495, -0.239 | <0.001 | 67.2 | 0.08 | <0.1 |
| HCs | Fear | Accuracy | Bjertrup et al., 2021 | -0.358 | -0.484, -0.232 | <0.001 | 66.28 | 0.08 | <0.1 |
| HCs | Fear | Accuracy | Fernandes et al., 2016 | -0.353 | -0.479, -0.228 | <0.001 | 65.63 | 0.07 | <0.1 |
| HCs | Fear | Accuracy | Golkhatmi et al., 2013 | -0.346 | -0.468, -0.223 | <0.001 | 63.67 | 0.07 | <0.1 |
| HCs | Fear | Accuracy | Baez et al., 2013 | -0.347 | -0.47, -0.225 | <0.001 | 64 | 0.07 | <0.1 |
| HCs | Fear | Accuracy | Derntl et al., 2012 | -0.366 | -0.494, -0.237 | <0.001 | 67.21 | 0.08 | <0.1 |
| HCs | Fear | Accuracy | Goghari et al., 2013 | -0.368 | -0.496, -0.24 | <0.001 | 67.21 | 0.08 | <0.1 |
| HCs | Fear | Accuracy | Pan et al., 2013 | -0.374 | -0.502, -0.245 | <0.001 | 66.89 | 0.08 | <0.1 |
| HCs | Fear | Accuracy | Kjaerstad et al., 2022 | -0.377 | -0.507, -0.247 | <0.001 | 64.44 | 0.08 | <0.1 |
| HCs | Fear | Accuracy | Harmer et al., 2002 | -0.38 | -0.503, -0.258 | <0.001 | 63.95 | 0.07 | <0.1 |
| HCs | Fear | Accuracy | Hoertnagl et al., 2011 | -0.372 | -0.501, -0.242 | <0.001 | 67.12 | 0.08 | <0.1 |
| HCs | Fear | Accuracy | Iakimova et al., 2016 | -0.338 | -0.456, -0.22 | <0.001 | 60.98 | 0.06 | <0.1 |
| HCs | Fear | Accuracy | Ulusoy et al., 2020 | -0.361 | -0.489, -0.233 | <0.001 | 66.91 | 0.08 | <0.1 |
| HCs | Fear | Accuracy | Jogia et al., 2011 | -0.376 | -0.504, -0.248 | <0.001 | 66.46 | 0.08 | <0.1 |
| HCs | Fear | Accuracy | Lawlor-Savage et al., 2014 | -0.366 | -0.494, -0.237 | <0.001 | 67.22 | 0.08 | <0.1 |
| HCs | Fear | Accuracy | Martino et al., 2011 | -0.355 | -0.482, -0.228 | <0.001 | 65.87 | 0.08 | <0.1 |
| HCs | Fear | Accuracy | Maila de Castro et al., 2015 | -0.341 | -0.46, -0.222 | <0.001 | 62.05 | 0.06 | <0.1 |
| HCs | Fear | Accuracy | Nigam et al., 2021 | -0.341 | -0.461, -0.221 | <0.001 | 62.14 | 0.06 | <0.1 |
| HCs | Fear | Accuracy | Robinson et al., 2015a | -0.373 | -0.501, -0.245 | <0.001 | 66.93 | 0.08 | <0.1 |
| HCs | Fear | Accuracy | Robinson et al., 2015b | -0.368 | -0.497, -0.238 | <0.001 | 67.28 | 0.08 | <0.1 |
| HCs | Fear | Accuracy | Rubin et al., 2022 | -0.381 | -0.507, -0.254 | <0.001 | 63.59 | 0.07 | <0.1 |
| HCs | Fear | Accuracy | Ruocco et al., 2014 | -0.379 | -0.508, -0.251 | <0.001 | 62.79 | 0.08 | <0.1 |
| HCs | Fear | Accuracy | Sagar et al., 2013 | -0.36 | -0.488, -0.233 | <0.001 | 66.76 | 0.08 | <0.1 |
| HCs | Fear | Accuracy | Schaefer et al., 2010 | -0.378 | -0.503, -0.253 | <0.001 | 65.5 | 0.07 | <0.1 |
| HCs | Fear | Accuracy | Shah et al., 2009 | -0.378 | -0.504, -0.251 | <0.001 | 65.83 | 0.08 | <0.1 |
| HCs | Fear | Accuracy | Soeiro-de-Souza et al., 2012a | -0.352 | -0.478, -0.225 | <0.001 | 65.09 | 0.08 | <0.1 |
| HCs | Fear | Accuracy | Soeiro-de-Souza et al., 2012b | -0.373 | -0.501, -0.244 | <0.001 | 66.99 | 0.08 | <0.1 |
| HCs | Fear | Accuracy | Van Rheenen et al., 2014 | -0.361 | -0.49, -0.232 | <0.001 | 66.9 | 0.08 | <0.1 |
| HCs | Fear | Accuracy | Vederman et al., 2012 | -0.38 | -0.506, -0.253 | <0.001 | 64.88 | 0.08 | <0.1 |
| HCs | Fear | Accuracy | Venn et al., 2004 | -0.357 | -0.483, -0.231 | <0.001 | 66.28 | 0.08 | <0.1 |
| HCs | Fear | Accuracy | Vierck et al., 2015 | -0.363 | -0.492, -0.234 | <0.001 | 67.1 | 0.08 | <0.1 |
| HCs | Fear | Accuracy | Yalcin-Siedentopf et al., 2014 | -0.374 | -0.503, -0.244 | <0.001 | 66.86 | 0.08 | <0.1 |
| HCs | Fear | Accuracy | Millet et al., 2023 | -0.371 | -0.502, -0.241 | <0.001 | 66.9 | 0.08 | <0.1 |
| HCs | Fear | Number of errors | Thaler et al., 2013 | 0.03 | -0.35, 0.41 | 0.88 | 8.19 | 0.01 | 0.3 |
| HCs | Fear | Number of errors | MacPherson et al., 2021 | -0.002 | -0.431, 0.426 | 0.99 | 0 | 0 | 0.33 |
| HCs | Fear | Number of errors | Malhi et al., 2007 | 0.118 | -0.175, 0.412 | 0.43 | 0 | 0 | 0.99 |
| HCs | Fear | Reaction time | Fernandes et al., 2016 | 0.403 | 0.208, 0.598 | <0.001 | 65.3 | 0.05 | <0.1 |
| HCs | Fear | Reaction time | Baez et al., 2013 | 0.324 | 0.161, 0.486 | <0.001 | 51.23 | 0.03 | <0.1 |
| HCs | Fear | Reaction time | Goghari et al., 2013 | 0.378 | 0.184, 0.573 | <0.001 | 65.57 | 0.05 | <0.1 |
| HCs | Fear | Reaction time | Kjaerstad et al., 2022 | 0.43 | 0.246, 0.613 | <0.001 | 47.79 | 0.03 | <0.1 |
| HCs | Fear | Reaction time | Harmer et al., 2002 | 0.397 | 0.202, 0.592 | <0.001 | 65.74 | 0.05 | <0.1 |
| HCs | Fear | Reaction time | Jogia et al., 2011 | 0.343 | 0.16, 0.527 | <0.001 | 58.73 | 0.04 | <0.1 |
| HCs | Fear | Reaction time | Malhi et al., 2007 | 0.378 | 0.189, 0.567 | <0.001 | 64.87 | 0.05 | <0.1 |
| HCs | Fear | Reaction time | Rubin et al., 2022 | 0.424 | 0.229, 0.619 | <0.001 | 56.53 | 0.04 | <0.1 |
| HCs | Fear | Reaction time | Ruocco et al., 2014 | 0.366 | 0.157, 0.576 | 0.001 | 58.15 | 0.06 | <0.1 |
| HCs | Fear | Reaction time | Shah et al., 2009 | 0.384 | 0.182, 0.585 | <0.001 | 66.38 | 0.06 | <0.1 |
| HCs | Fear | Reaction time | Vierck et al., 2015 | 0.371 | 0.174, 0.569 | <0.001 | 65.19 | 0.05 | <0.1 |
| HCs | Happiness | Accuracy | Almeida et al., 2010 | -0.18 | -0.258, -0.102 | <0.001 | 22.45 | 0.01 | <0.1 |
| HCs | Happiness | Accuracy | Fernandes et al., 2016 | -0.19 | -0.271, -0.108 | <0.001 | 27.37 | 0.01 | <0.1 |
| HCs | Happiness | Accuracy | Golkhatmi et al., 2013 | -0.188 | -0.269, -0.106 | <0.001 | 26.71 | 0.01 | <0.1 |
| HCs | Happiness | Accuracy | Baez et al., 2013 | -0.187 | -0.268, -0.106 | <0.001 | 26.15 | 0.01 | <0.1 |
| HCs | Happiness | Accuracy | Derntl et al., 2012 | -0.19 | -0.272, -0.109 | <0.001 | 27.48 | 0.01 | <0.1 |
| HCs | Happiness | Accuracy | Goghari et al., 2013 | -0.198 | -0.278, -0.118 | <0.001 | 25.15 | 0.01 | <0.1 |
| HCs | Happiness | Accuracy | Pan et al., 2013 | -0.197 | -0.28, -0.115 | <0.001 | 28.22 | 0.01 | <0.1 |
| HCs | Happiness | Accuracy | Kjaerstad et al., 2022 | -0.203 | -0.288, -0.118 | <0.001 | 26.96 | 0.02 | <0.1 |
| HCs | Happiness | Accuracy | Harmer et al., 2002 | -0.193 | -0.274, -0.111 | <0.001 | 27.83 | 0.01 | <0.1 |
| HCs | Happiness | Accuracy | Hoertnagl et al., 2011 | -0.164 | -0.235, -0.092 | <0.001 | 10.64 | 0 | 0.12 |
| HCs | Happiness | Accuracy | Iakimova et al., 2016 | -0.193 | -0.276, -0.111 | <0.001 | 28.39 | 0.01 | <0.1 |
| HCs | Happiness | Accuracy | Ulusoy et al., 2020 | -0.181 | -0.26, -0.102 | <0.001 | 23.39 | 0.01 | <0.1 |
| HCs | Happiness | Accuracy | Lawlor-Savage et al., 2014 | -0.168 | -0.242, -0.095 | <0.001 | 14.02 | 0.01 | 0.15 |
| HCs | Happiness | Accuracy | Martino et al., 2011 | -0.198 | -0.281, -0.115 | <0.001 | 28.1 | 0.01 | <0.1 |
| HCs | Happiness | Accuracy | Maila de Castro et al., 2015 | -0.182 | -0.261, -0.103 | <0.001 | 23.45 | 0.01 | <0.1 |
| HCs | Happiness | Accuracy | Nigam et al., 2021 | -0.183 | -0.262, -0.103 | <0.001 | 24.1 | 0.01 | <0.1 |
| HCs | Happiness | Accuracy | Lescalier et al., 2015 | -0.198 | -0.279, -0.116 | <0.001 | 27.05 | 0.01 | <0.1 |
| HCs | Happiness | Accuracy | Robinson et al., 2015a | -0.199 | -0.279, -0.119 | <0.001 | 24.71 | 0.01 | <0.1 |
| HCs | Happiness | Accuracy | Robinson et al., 2015b | -0.195 | -0.279, -0.112 | <0.001 | 29.13 | 0.02 | <0.1 |
| HCs | Happiness | Accuracy | Rubin et al., 2022 | -0.201 | -0.287, -0.116 | <0.001 | 28.67 | 0.02 | <0.1 |
| HCs | Happiness | Accuracy | Ruocco et al., 2014 | -0.203 | -0.289, -0.117 | <0.001 | 27.52 | 0.02 | <0.1 |
| HCs | Happiness | Accuracy | Sagar et al., 2013 | -0.191 | -0.272, -0.109 | <0.001 | 27.44 | 0.01 | <0.1 |
| HCs | Happiness | Accuracy | Schaefer et al., 2010 | -0.194 | -0.276, -0.113 | <0.001 | 27.95 | 0.01 | <0.1 |
| HCs | Happiness | Accuracy | Shah et al., 2009 | -0.197 | -0.28, -0.115 | <0.001 | 27.86 | 0.01 | <0.1 |
| HCs | Happiness | Accuracy | Soeiro-de-Souza et al., 2012a | -0.191 | -0.274, -0.107 | <0.001 | 28.73 | 0.02 | <0.1 |
| HCs | Happiness | Accuracy | Soeiro-de-Souza et al., 2012b | -0.197 | -0.28, -0.115 | <0.001 | 28.04 | 0.01 | <0.1 |
| HCs | Happiness | Accuracy | Tesli et al., 2015 | -0.202 | -0.283, -0.12 | <0.001 | 24.86 | 0.01 | <0.1 |
| HCs | Happiness | Accuracy | Van Rheenen et al., 2014 | -0.19 | -0.273, -0.107 | <0.001 | 28.36 | 0.02 | <0.1 |
| HCs | Happiness | Accuracy | Vederman et al., 2012 | -0.201 | -0.284, -0.118 | <0.001 | 27.16 | 0.01 | <0.1 |
| HCs | Happiness | Accuracy | Venn et al., 2004 | -0.188 | -0.268, -0.107 | <0.001 | 26.39 | 0.01 | <0.1 |
| HCs | Happiness | Accuracy | Versace et al., 2010 | -0.178 | -0.256, -0.101 | <0.001 | 21.28 | 0.01 | <0.1 |
| HCs | Happiness | Accuracy | Vierck et al., 2015 | -0.182 | -0.261, -0.102 | <0.001 | 23.84 | 0.01 | <0.1 |
| HCs | Happiness | Accuracy | Yalcin-Siedentopf et al., 2014 | -0.168 | -0.242, -0.094 | <0.001 | 14 | 0.01 | <0.1 |
| HCs | Happiness | Accuracy | Zhang et al., 2018 | -0.191 | -0.274, -0.107 | <0.001 | 28.58 | 0.02 | <0.1 |
| HCs | Happiness | Accuracy | Millet et al., 2023 | -0.194 | -0.278, -0.109 | <0.001 | 29.76 | 0.02 | <0.1 |
| HCs | Happiness | Number of errors | Thaler et al., 2013 | 0.076 | -0.183, 0.335 | 0.56 | 0 | 0 | 1 |
| HCs | Happiness | Number of errors | MacPherson et al., 2021.1 | 0.057 | -0.236, 0.35 | 0.7 | 0 | 0 | 0.86 |
| HCs | Happiness | Number of errors | MacPherson et al., 2021.2 | 0.057 | -0.236, 0.35 | 0.7 | 0 | 0 | 0.86 |
| HCs | Happiness | Reaction time | Altamura et al., 2016 | 0.44 | 0.214, 0.665 | <0.001 | 79.7 | 0.1 | <0.1 |
| HCs | Happiness | Reaction time | Fernandes et al., 2016 | 0.517 | 0.217, 0.817 | 0.001 | 88.69 | 0.2 | <0.1 |
| HCs | Happiness | Reaction time | Baez et al., 2013 | 0.527 | 0.222, 0.831 | 0.001 | 89.15 | 0.21 | <0.1 |
| HCs | Happiness | Reaction time | Goghari et al., 2013 | 0.578 | 0.264, 0.892 | <0.001 | 89.79 | 0.23 | <0.1 |
| HCs | Happiness | Reaction time | Kjaerstad et al., 2022 | 0.605 | 0.299, 0.912 | <0.001 | 85.88 | 0.21 | <0.1 |
| HCs | Happiness | Reaction time | Harmer et al., 2002 | 0.538 | 0.228, 0.848 | 0.001 | 89.56 | 0.22 | <0.1 |
| HCs | Happiness | Reaction time | Rubin et al., 2022 | 0.608 | 0.306, 0.909 | <0.001 | 86.43 | 0.2 | <0.1 |
| HCs | Happiness | Reaction time | Ruocco et al., 2014 | 0.522 | 0.209, 0.835 | 0.001 | 85.93 | 0.22 | <0.1 |
| HCs | Happiness | Reaction time | Shah et al., 2009 | 0.579 | 0.26, 0.897 | <0.001 | 89.76 | 0.23 | <0.1 |
| HCs | Happiness | Reaction time | Tesli et al., 2015 | 0.582 | 0.261, 0.904 | <0.001 | 89.05 | 0.24 | <0.1 |
| HCs | Happiness | Reaction time | Vierck et al., 2015 | 0.553 | 0.234, 0.872 | 0.001 | 89.81 | 0.23 | <0.1 |
| HCs | Happiness | Reaction time | Zhang et al., 2018 | 0.608 | 0.311, 0.905 | <0.001 | 87.64 | 0.19 | <0.1 |
| HCs | Neutral | Accuracy | Almeida et al., 2010 | -0.161 | -0.284, -0.038 | 0.01 | 27.66 | 0.01 | 0.22 |
| HCs | Neutral | Accuracy | Fernandes et al., 2016 | -0.17 | -0.295, -0.046 | 0.007 | 28.03 | 0.01 | 0.2 |
| HCs | Neutral | Accuracy | Derntl et al., 2012 | -0.162 | -0.286, -0.038 | 0.01 | 28.02 | 0.01 | 0.2 |
| HCs | Neutral | Accuracy | Goghari et al., 2013 | -0.173 | -0.297, -0.05 | 0.006 | 27.47 | 0.01 | 0.2 |
| HCs | Neutral | Accuracy | Kjaerstad et al., 2022 | -0.187 | -0.32, -0.053 | 0.006 | 23.57 | 0.02 | 0.29 |
| HCs | Neutral | Accuracy | Harmer et al., 2002 | -0.176 | -0.298, -0.053 | 0.005 | 26.97 | 0.01 | 0.22 |
| HCs | Neutral | Accuracy | Iakimova et al., 2016 | -0.151 | -0.273, -0.029 | 0.015 | 25.28 | 0.01 | 0.3 |
| HCs | Neutral | Accuracy | Maila de Castro et al., 2015 | -0.181 | -0.302, -0.06 | 0.003 | 25.65 | 0.01 | 0.25 |
| HCs | Neutral | Accuracy | Nigam et al., 2021 | -0.164 | -0.289, -0.039 | 0.01 | 28.33 | 0.01 | 0.2 |
| HCs | Neutral | Accuracy | Lescalier et al., 2015 | -0.171 | -0.295, -0.046 | 0.007 | 28.14 | 0.01 | 0.2 |
| HCs | Neutral | Accuracy | Robinson et al., 2015a | -0.167 | -0.293, -0.041 | 0.009 | 28.55 | 0.02 | 0.2 |
| HCs | Neutral | Accuracy | Ruocco et al., 2014 | -0.118 | -0.227, -0.009 | 0.034 | 0 | 0 | 0.54 |
| HCs | Neutral | Accuracy | Shah et al., 2009 | -0.194 | -0.312, -0.076 | 0.001 | 20.98 | 0.01 | 0.37 |
| HCs | Neutral | Accuracy | Vierck et al., 2015 | -0.191 | -0.31, -0.071 | 0.002 | 22.4 | 0.01 | 0.32 |
| HCs | Neutral | Accuracy | Zhang et al., 2018 | -0.181 | -0.307, -0.055 | 0.005 | 26.59 | 0.01 | 0.23 |
| HCs | Neutral | Accuracy | Miola et al., 2023 | -0.148 | -0.27, -0.026 | 0.017 | 24.13 | 0.01 | 0.32 |
| HCs | Neutral | Reaction time | Fernandes et al., 2016 | 0.324 | 0.12, 0.527 | 0.002 | 62.79 | 0.05 | <0.1 |
| HCs | Neutral | Reaction time | Goghari et al., 2013 | 0.297 | 0.088, 0.506 | 0.005 | 65.13 | 0.05 | <0.1 |
| HCs | Neutral | Reaction time | Kjaerstad et al., 2022 | 0.384 | 0.218, 0.55 | <0.001 | 26.45 | 0.02 | 0.19 |
| HCs | Neutral | Reaction time | Harmer et al., 2002 | 0.268 | 0.067, 0.47 | 0.009 | 62.87 | 0.05 | <0.1 |
| HCs | Neutral | Reaction time | Malhi et al., 2007 | 0.26 | 0.067, 0.453 | 0.008 | 61.05 | 0.04 | <0.1 |
| HCs | Neutral | Reaction time | Ruocco et al., 2014 | 0.218 | 0.025, 0.411 | 0.027 | 40.61 | 0.03 | <0.1 |
| HCs | Neutral | Reaction time | Shah et al., 2009 | 0.286 | 0.073, 0.499 | 0.009 | 64.86 | 0.06 | <0.1 |
| HCs | Neutral | Reaction time | Vierck et al., 2015 | 0.293 | 0.079, 0.508 | 0.007 | 65.18 | 0.06 | <0.1 |
| HCs | Neutral | Reaction time | Zhang et al., 2018 | 0.308 | 0.091, 0.526 | 0.006 | 64.54 | 0.06 | <0.1 |
| HCs | Neutral | Reaction time | Miola et al., 2023 | 0.294 | 0.078, 0.51 | 0.008 | 65.13 | 0.06 | <0.1 |
| HCs | Sadness | Accuracy | Almeida et al., 2010 | -0.22 | -0.325, -0.116 | <0.001 | 54.64 | 0.05 | <0.1 |
| HCs | Sadness | Accuracy | Bjertrup et al., 2021 | -0.217 | -0.32, -0.114 | <0.001 | 53.52 | 0.04 | <0.1 |
| HCs | Sadness | Accuracy | Branco et al., 2017 | -0.224 | -0.33, -0.117 | <0.001 | 55.51 | 0.05 | <0.1 |
| HCs | Sadness | Accuracy | Fernandes et al., 2016 | -0.215 | -0.318, -0.112 | <0.001 | 53.08 | 0.04 | <0.1 |
| HCs | Sadness | Accuracy | Golkhatmi et al., 2013 | -0.194 | -0.285, -0.103 | <0.001 | 40.17 | 0.03 | <0.1 |
| HCs | Sadness | Accuracy | Baez et al., 2013 | -0.203 | -0.3, -0.107 | <0.001 | 46.97 | 0.03 | <0.1 |
| HCs | Sadness | Accuracy | Derntl et al., 2012 | -0.219 | -0.324, -0.115 | <0.001 | 54.47 | 0.05 | <0.1 |
| HCs | Sadness | Accuracy | Goghari et al., 2013 | -0.231 | -0.336, -0.126 | <0.001 | 54.85 | 0.05 | <0.1 |
| HCs | Sadness | Accuracy | Pan et al., 2013 | -0.23 | -0.336, -0.123 | <0.001 | 55.65 | 0.05 | <0.1 |
| HCs | Sadness | Accuracy | Kjaerstad et al., 2022 | -0.229 | -0.338, -0.12 | <0.001 | 54.53 | 0.05 | <0.1 |
| HCs | Sadness | Accuracy | Harmer et al., 2002 | -0.223 | -0.328, -0.118 | <0.001 | 55.12 | 0.05 | <0.1 |
| HCs | Sadness | Accuracy | Hoertnagl et al., 2011 | -0.217 | -0.321, -0.112 | <0.001 | 53.87 | 0.05 | <0.1 |
| HCs | Sadness | Accuracy | Iakimova et al., 2016 | -0.236 | -0.325, -0.148 | <0.001 | 36.5 | 0.02 | <0.1 |
| HCs | Sadness | Accuracy | Ulusoy et al., 2020 | -0.222 | -0.327, -0.116 | <0.001 | 55.18 | 0.05 | <0.1 |
| HCs | Sadness | Accuracy | Lawlor-Savage et al., 2014 | -0.222 | -0.327, -0.116 | <0.001 | 55.05 | 0.05 | <0.1 |
| HCs | Sadness | Accuracy | Lelli-Chiesa et al., 2010 | -0.218 | -0.323, -0.113 | <0.001 | 54.23 | 0.05 | <0.1 |
| HCs | Sadness | Accuracy | Martino et al., 2011 | -0.234 | -0.339, -0.128 | <0.001 | 54.47 | 0.05 | <0.1 |
| HCs | Sadness | Accuracy | Maila de Castro et al., 2015 | -0.214 | -0.316, -0.112 | <0.001 | 52.51 | 0.04 | <0.1 |
| HCs | Sadness | Accuracy | Nigam et al., 2021 | -0.226 | -0.332, -0.12 | <0.001 | 55.6 | 0.05 | <0.1 |
| HCs | Sadness | Accuracy | Lescalier et al., 2015 | -0.231 | -0.336, -0.125 | <0.001 | 55.18 | 0.05 | <0.1 |
| HCs | Sadness | Accuracy | Robinson et al., 2015a | -0.232 | -0.338, -0.127 | <0.001 | 54.74 | 0.05 | <0.1 |
| HCs | Sadness | Accuracy | Robinson et al., 2015b | -0.234 | -0.339, -0.128 | <0.001 | 54.57 | 0.05 | <0.1 |
| HCs | Sadness | Accuracy | Rubin et al., 2022 | -0.239 | -0.342, -0.136 | <0.001 | 49.67 | 0.04 | <0.1 |
| HCs | Sadness | Accuracy | Ruocco et al., 2014 | -0.236 | -0.343, -0.129 | <0.001 | 51.66 | 0.05 | <0.1 |
| HCs | Sadness | Accuracy | Schaefer et al., 2010 | -0.227 | -0.332, -0.121 | <0.001 | 55.5 | 0.05 | <0.1 |
| HCs | Sadness | Accuracy | Soeiro-de-Souza et al., 2012a | -0.219 | -0.325, -0.113 | <0.001 | 54.59 | 0.05 | <0.1 |
| HCs | Sadness | Accuracy | Soeiro-de-Souza et al., 2012b | -0.226 | -0.332, -0.119 | <0.001 | 55.74 | 0.05 | <0.1 |
| HCs | Sadness | Accuracy | Van Rheenen et al., 2014 | -0.226 | -0.334, -0.119 | <0.001 | 55.85 | 0.05 | <0.1 |
| HCs | Sadness | Accuracy | Vederman et al., 2012 | -0.234 | -0.341, -0.128 | <0.001 | 54.28 | 0.05 | <0.1 |
| HCs | Sadness | Accuracy | Venn et al., 2004 | -0.23 | -0.335, -0.126 | <0.001 | 54.88 | 0.05 | <0.1 |
| HCs | Sadness | Accuracy | Versace et al., 2010 | -0.224 | -0.33, -0.119 | <0.001 | 55.48 | 0.05 | <0.1 |
| HCs | Sadness | Accuracy | Vierck et al., 2015 | -0.221 | -0.327, -0.116 | <0.001 | 55.16 | 0.05 | <0.1 |
| HCs | Sadness | Accuracy | Yalcin-Siedentopf et al., 2014 | -0.22 | -0.326, -0.114 | <0.001 | 54.92 | 0.05 | <0.1 |
| HCs | Sadness | Accuracy | Zhang et al., 2018 | -0.227 | -0.335, -0.12 | <0.001 | 55.87 | 0.05 | <0.1 |
| HCs | Sadness | Accuracy | Millet et al., 2023 | -0.23 | -0.338, -0.122 | <0.001 | 55.65 | 0.05 | <0.1 |
| HCs | Sadness | Accuracy | Miola et al., 2023 | -0.227 | -0.334, -0.12 | <0.001 | 55.82 | 0.05 | <0.1 |
| HCs | Sadness | Reaction time | Fernandes et al., 2016 | 0.352 | 0.137, 0.568 | 0.001 | 75.68 | 0.08 | <0.1 |
| HCs | Sadness | Reaction time | Baez et al., 2013 | 0.273 | 0.087, 0.46 | 0.004 | 67.68 | 0.05 | <0.1 |
| HCs | Sadness | Reaction time | Goghari et al., 2013 | 0.343 | 0.127, 0.56 | 0.002 | 76.23 | 0.08 | <0.1 |
| HCs | Sadness | Reaction time | Kjaerstad et al., 2022 | 0.391 | 0.209, 0.572 | <0.001 | 53.99 | 0.04 | <0.1 |
| HCs | Sadness | Reaction time | Harmer et al., 2002 | 0.325 | 0.109, 0.54 | 0.003 | 76.07 | 0.08 | <0.1 |
| HCs | Sadness | Reaction time | Lelli-Chiesa et al., 2010 | 0.292 | 0.086, 0.499 | 0.006 | 72.19 | 0.07 | <0.1 |
| HCs | Sadness | Reaction time | Rubin et al., 2022 | 0.365 | 0.143, 0.586 | 0.001 | 71.8 | 0.08 | <0.1 |
| HCs | Sadness | Reaction time | Ruocco et al., 2014 | 0.296 | 0.078, 0.515 | 0.008 | 67.45 | 0.07 | <0.1 |
| HCs | Sadness | Reaction time | Vierck et al., 2015 | 0.314 | 0.097, 0.532 | 0.005 | 75.45 | 0.08 | <0.1 |
| HCs | Sadness | Reaction time | Zhang et al., 2018 | 0.339 | 0.113, 0.564 | 0.003 | 76.32 | 0.09 | <0.1 |
| HCs | Sadness | Reaction time | Miola et al., 2023 | 0.345 | 0.123, 0.567 | 0.002 | 76.3 | 0.08 | <0.1 |
| HCs | Surprise | Accuracy | Bjertrup et al., 2021 | -0.299 | -0.394, -0.204 | <0.001 | 0 | 0 | 0.4 |
| HCs | Surprise | Accuracy | Golkhatmi et al., 2013 | -0.29 | -0.386, -0.194 | <0.001 | 0 | 0 | 0.52 |
| HCs | Surprise | Accuracy | Baez et al., 2013 | -0.297 | -0.393, -0.202 | <0.001 | 0 | 0 | 0.38 |
| HCs | Surprise | Accuracy | Summers et al., 2006 | -0.311 | -0.41, -0.212 | <0.001 | 2.67 | 0 | 0.26 |
| HCs | Surprise | Accuracy | Kjaerstad et al., 2022 | -0.31 | -0.429, -0.19 | <0.001 | 10.96 | 0.01 | 0.26 |
| HCs | Surprise | Accuracy | Harmer et al., 2002 | -0.313 | -0.41, -0.216 | <0.001 | 1.37 | 0 | 0.28 |
| HCs | Surprise | Accuracy | Hoertnagl et al., 2011 | -0.308 | -0.409, -0.208 | <0.001 | 3.32 | 0 | 0.26 |
| HCs | Surprise | Accuracy | Iakimova et al., 2016 | -0.308 | -0.406, -0.209 | <0.001 | 1.94 | 0 | 0.26 |
| HCs | Surprise | Accuracy | Ulusoy et al., 2020 | -0.296 | -0.393, -0.2 | <0.001 | 0.02 | 0 | 0.33 |
| HCs | Surprise | Accuracy | Martino et al., 2011 | -0.321 | -0.42, -0.222 | <0.001 | 1.61 | 0 | 0.32 |
| HCs | Surprise | Accuracy | Nigam et al., 2021 | -0.294 | -0.39, -0.198 | <0.001 | 0 | 0 | 0.4 |
| HCs | Surprise | Accuracy | Rubin et al., 2022 | -0.342 | -0.446, -0.238 | <0.001 | 0 | 0 | 0.4 |
| HCs | Surprise | Accuracy | Schaefer et al., 2010 | -0.316 | -0.413, -0.219 | <0.001 | 1.19 | 0 | 0.3 |
| HCs | Surprise | Accuracy | Soeiro-de-Souza et al., 2012a | -0.312 | -0.418, -0.207 | <0.001 | 6.14 | 0 | 0.26 |
| HCs | Surprise | Accuracy | Soeiro-de-Souza et al., 2012b | -0.321 | -0.418, -0.225 | <0.001 | 0.11 | 0 | 0.36 |
| HCs | Surprise | Accuracy | Venn et al., 2004 | -0.308 | -0.404, -0.212 | <0.001 | 0.79 | 0 | 0.26 |
| HCs | Surprise | Accuracy | Yalcin-Siedentopf et al., 2014 | -0.31 | -0.412, -0.208 | <0.001 | 4.35 | 0 | 0.26 |
| HCs | Surprise | Reaction time | Baez et al., 2013 | 0.159 | 0.019, 0.299 | 0.026 | 0.01 | 0 | 0.44 |
| HCs | Surprise | Reaction time | Kjaerstad et al., 2022 | 0.617 | -0.043, 1.277 | 0.07 | 80.58 | 0.27 | <0.1 |
| HCs | Surprise | Reaction time | Harmer et al., 2002 | 0.462 | -0.224, 1.149 | 0.19 | 94.37 | 0.33 | <0.1 |
| HCs | Surprise | Reaction time | Rubin et al., 2022 | 0.603 | -0.081, 1.287 | 0.08 | 82.63 | 0.29 | <0.1 |
| FDRs | Anger | Accuracy | Fernandes et al., 2016 | 0.11 | -0.122, 0.342 | 0.35 | 57.52 | 0.04 | <0.1 |
| FDRs | Anger | Accuracy | Kjaerstad et al., 2022 | 0.039 | -0.224, 0.302 | 0.77 | 50.64 | 0.04 | <0.1 |
| FDRs | Anger | Accuracy | Ulusoy et al., 2020 | -0.002 | -0.182, 0.178 | 0.98 | 28.53 | 0.01 | 0.35 |
| FDRs | Anger | Accuracy | Nigam et al., 2021 | 0.081 | -0.162, 0.323 | 0.52 | 60.99 | 0.04 | <0.1 |
| FDRs | Anger | Accuracy | Ruocco et al., 2014 | 0.16 | -0.066, 0.386 | 0.16 | 26.45 | 0.02 | 0.23 |
| FDRs | Anger | Accuracy | Vierck et al., 2015 | 0.102 | -0.141, 0.345 | 0.41 | 59.61 | 0.04 | <0.1 |
| FDRs | Anger | Reaction time | Fernandes et al., 2016 | 0.132 | -0.108, 0.373 | 0.28 | 55.43 | 0.02 | 0.11 |
| FDRs | Anger | Reaction time | Kjaerstad et al., 2022 | 0.199 | -0.035, 0.433 | 0.1 | 22.79 | 0.01 | 0.35 |
| FDRs | Anger | Reaction time | Ruocco et al., 2014 | -0.009 | -0.222, 0.204 | 0.94 | 0 | 0 | 0.99 |
| FDRs | Anger | Reaction time | Vierck et al., 2015 | 0.144 | -0.096, 0.385 | 0.24 | 53.12 | 0.02 | 0.13 |
| FDRs | Disgust | Accuracy | Kjaerstad et al., 2022 | -0.471 | -0.96, 0.019 | 0.06 | 35.57 | 0.04 | 0.21 |
| FDRs | Disgust | Accuracy | Nigam et al., 2021 | -0.199 | -1.135, 0.738 | 0.68 | 90.11 | 0.41 | <0.1 |
| FDRs | Disgust | Accuracy | Vierck et al., 2015 | 0.099 | -0.323, 0.521 | 0.65 | 50.01 | 0.05 | 0.16 |
| FDRs | Fear | Accuracy | Fernandes et al., 2016 | -0.067 | -0.397, 0.263 | 0.69 | 80.79 | 0.13 | <0.1 |
| FDRs | Fear | Accuracy | Kjaerstad et al., 2022 | -0.158 | -0.534, 0.219 | 0.41 | 79.03 | 0.16 | <0.1 |
| FDRs | Fear | Accuracy | Ulusoy et al., 2020 | -0.18 | -0.509, 0.149 | 0.28 | 78.34 | 0.12 | <0.1 |
| FDRs | Fear | Accuracy | Jogia et al., 2011 | -0.129 | -0.497, 0.24 | 0.49 | 84.07 | 0.16 | <0.1 |
| FDRs | Fear | Accuracy | Nigam et al., 2021 | 0.028 | -0.095, 0.15 | 0.66 | 0.01 | 0 | 0.46 |
| FDRs | Fear | Accuracy | Ruocco et al., 2014 | -0.147 | -0.534, 0.24 | 0.46 | 77.16 | 0.17 | <0.1 |
| FDRs | Fear | Accuracy | Vierck et al., 2015 | -0.155 | -0.512, 0.202 | 0.39 | 83.11 | 0.15 | <0.1 |
| FDRs | Fear | Reaction time | Fernandes et al., 2016 | 0.171 | -0.21, 0.551 | 0.38 | 82.52 | 0.12 | <0.1 |
| FDRs | Fear | Reaction time | Kjaerstad et al., 2022 | 0.187 | -0.219, 0.594 | 0.37 | 73.03 | 0.12 | <0.1 |
| FDRs | Fear | Reaction time | Jogia et al., 2011 | 0.025 | -0.28, 0.33 | 0.87 | 70.9 | 0.06 | <0.1 |
| FDRs | Fear | Reaction time | Ruocco et al., 2014 | 0.045 | -0.36, 0.449 | 0.83 | 68.98 | 0.11 | <0.1 |
| FDRs | Fear | Reaction time | Vierck et al., 2015 | 0.206 | -0.137, 0.548 | 0.24 | 77.18 | 0.09 | <0.1 |
| FDRs | Happiness | Accuracy | Fernandes et al., 2016 | -0.112 | -0.319, 0.096 | 0.29 | 47.59 | 0.02 | 0.12 |
| FDRs | Happiness | Accuracy | Kjaerstad et al., 2022 | -0.202 | -0.419, 0.016 | 0.07 | 32.15 | 0.02 | 0.22 |
| FDRs | Happiness | Accuracy | Ulusoy et al., 2020 | -0.063 | -0.192, 0.067 | 0.34 | 0 | 0 | 0.54 |
| FDRs | Happiness | Accuracy | Nigam et al., 2021 | -0.171 | -0.392, 0.049 | 0.13 | 53.01 | 0.03 | 0.11 |
| FDRs | Happiness | Accuracy | Ruocco et al., 2014 | -0.166 | -0.448, 0.117 | 0.25 | 50.41 | 0.05 | <0.1 |
| FDRs | Happiness | Accuracy | Vierck et al., 2015 | -0.143 | -0.377, 0.091 | 0.23 | 56.63 | 0.04 | <0.1 |
| FDRs | Happiness | Reaction time | Fernandes et al., 2016 | 0.227 | -0.152, 0.606 | 0.24 | 81.47 | 0.09 | <0.1 |
| FDRs | Happiness | Reaction time | Kjaerstad et al., 2022 | 0.464 | 0.306, 0.622 | <0.001 | 0 | 0 | 0.64 |
| FDRs | Happiness | Reaction time | Ruocco et al., 2014 | 0.087 | -0.213, 0.387 | 0.57 | 33.32 | 0.03 | 0.26 |
| FDRs | Happiness | Reaction time | Vierck et al., 2015 | 0.249 | -0.145, 0.644 | 0.22 | 81.77 | 0.09 | <0.1 |
| FDRs | Neutral | Accuracy | Fernandes et al., 2016 | -0.085 | -0.253, 0.084 | 0.33 | 21.51 | 0.01 | 0.38 |
| FDRs | Neutral | Accuracy | Kjaerstad et al., 2022 | -0.148 | -0.299, 0.002 | 0.05 | 0 | 0 | 0.9 |
| FDRs | Neutral | Accuracy | Nigam et al., 2021 | -0.06 | -0.229, 0.109 | 0.49 | 21.63 | 0.01 | 0.44 |
| FDRs | Neutral | Accuracy | Ruocco et al., 2014 | 0.005 | -0.195, 0.205 | 0.96 | 0 | 0 | 0.6 |
| FDRs | Neutral | Accuracy | Vierck et al., 2015 | -0.069 | -0.244, 0.107 | 0.44 | 23.59 | 0.01 | 0.37 |
| FDRs | Neutral | Reaction time | Fernandes et al., 2016 | -0.045 | -0.297, 0.206 | 0.72 | 58.87 | 0.03 | <0.1 |
| FDRs | Neutral | Reaction time | Kjaerstad et al., 2022 | 0.066 | -0.09, 0.222 | 0.4 | 0 | 0 | 0.53 |
| FDRs | Neutral | Reaction time | Ruocco et al., 2014 | -0.206 | -0.42, 0.007 | 0.06 | 0 | 0 | 0.68 |
| FDRs | Neutral | Reaction time | Vierck et al., 2015 | -0.087 | -0.359, 0.186 | 0.53 | 62.52 | 0.03 | <0.1 |
| FDRs | Sadness | Accuracy | Fernandes et al., 2016 | -0.064 | -0.186, 0.059 | 0.31 | 0 | 0 | 0.73 |
| FDRs | Sadness | Accuracy | Kjaerstad et al., 2022 | -0.079 | -0.215, 0.057 | 0.26 | 0 | 0 | 0.76 |
| FDRs | Sadness | Accuracy | Ulusoy et al., 2020 | -0.09 | -0.215, 0.036 | 0.16 | 0 | 0 | 0.96 |
| FDRs | Sadness | Accuracy | Lelli-Chiesa et al., 2010 | -0.072 | -0.196, 0.051 | 0.25 | 0 | 0 | 0.77 |
| FDRs | Sadness | Accuracy | Nigam et al., 2021 | -0.065 | -0.188, 0.058 | 0.3 | 0 | 0 | 0.73 |
| FDRs | Sadness | Accuracy | Ruocco et al., 2014 | 0.011 | -0.157, 0.18 | 0.9 | 0 | 0 | 0.94 |
| FDRs | Sadness | Accuracy | Vierck et al., 2015 | -0.062 | -0.185, 0.061 | 0.32 | 0 | 0 | 0.73 |
| FDRs | Sadness | Reaction time | Fernandes et al., 2016 | 0.225 | -0.216, 0.665 | 0.32 | 87.04 | 0.17 | <0.1 |
| FDRs | Sadness | Reaction time | Kjaerstad et al., 2022 | 0.242 | -0.213, 0.697 | 0.3 | 78.26 | 0.16 | <0.1 |
| FDRs | Sadness | Reaction time | Lelli-Chiesa et al., 2010 | 0.007 | -0.328, 0.341 | 0.97 | 75.92 | 0.08 | <0.1 |
| FDRs | Sadness | Reaction time | Ruocco et al., 2014 | 0.068 | -0.431, 0.566 | 0.79 | 79.45 | 0.2 | <0.1 |
| FDRs | Sadness | Reaction time | Vierck et al., 2015 | 0.19 | -0.295, 0.675 | 0.44 | 88.86 | 0.2 | <0.1 |
| FDRs | Surprise | Accuracy | Kjaerstad et al., 2022 | -0.241 | -1.21, 0.727 | 0.62 | 86.06 | 0.42 | <0.1 |
| FDRs | Surprise | Accuracy | Ulusoy et al., 2020 | -0.353 | -1.04, 0.334 | 0.31 | 78.09 | 0.2 | <0.1 |
| FDRs | Surprise | Accuracy | Nigam et al., 2021 | 0.041 | -0.218, 0.3 | 0.76 | 23.46 | 0.01 | 0.25 |
| *Children/adolescents* | | | | | | | | | |
| Level I | | | | | | | | | |
| HCs | Any emotion | Accuracy | Bozorg et al., 2014 | -0.532 | -0.791, -0.272 | <0.001 | 0 | 0 | 0.42 |
| HCs | Any emotion | Accuracy | Khafif et al., 2023 | -0.655 | -0.914, -0.396 | <0.001 | 0 | 0 | 0.48 |
| HCs | Any emotion | Accuracy | Pavuluri et al., 2009 | -0.633 | -0.875, -0.391 | <0.001 | 0 | 0 | 0.57 |
| HCs | Any emotion | Accuracy | Kim et al., 2013 | -0.604 | -0.865, -0.342 | <0.001 | 5.96 | 0.01 | 0.32 |
| HCs | Any emotion | Accuracy | Schenkel et al., 2012 | -0.512 | -0.776, -0.247 | <0.001 | 0 | 0 | 0.5 |
| HCs | Any emotion | Accuracy | Schenkel et al., 2020 | -0.574 | -0.85, -0.298 | <0.001 | 12.72 | 0.01 | 0.31 |
| HCs | Any emotion | Number of errors | Brotman et al., 2008 | 0.624 | 0.364, 0.884 | <0.001 | 0 | 0 | 0.8 |
| HCs | Any emotion | Number of errors | Guyer et al., 2007 | 0.718 | 0.461, 0.976 | <0.001 | 0 | 0 | 1 |
| HCs | Any emotion | Number of errors | McClure et al., 2005 | 0.651 | 0.422, 0.879 | <0.001 | 0 | 0 | 0.74 |
| HCs | Any emotion | Number of errors | Seymour et al., 2013 | 0.646 | 0.411, 0.88 | <0.001 | 0 | 0 | 0.75 |
| HCs | Any emotion | Reaction time | Bozorg et al., 2014 | 0.157 | -0.298, 0.612 | 0.5 | 0 | 0 | 0.52 |
| HCs | Any emotion | Reaction time | Khafif et al., 2023 | 0.352 | -0.298, 1.003 | 0.29 | 43.39 | 0.1 | 0.18 |
| HCs | Any emotion | Reaction time | Pavuluri et al., 2009 | 0.43 | 0.059, 0.801 | 0.023 | 0 | 0 | 0.35 |
| Level II | | | | | | | | | |
| HCs | Negative | Accuracy | Bozorg et al., 2014 | -0.508 | -0.81, -0.205 | 0.001 | 0 | 0 | 0.57 |
| HCs | Negative | Accuracy | Khafif et al., 2023 | -0.611 | -0.911, -0.311 | <0.001 | 0 | 0 | 0.8 |
| HCs | Negative | Accuracy | Schenkel et al., 2012 | -0.475 | -0.785, -0.165 | 0.003 | 0 | 0 | 0.7 |
| HCs | Negative | Accuracy | Schenkel et al., 2020 | -0.564 | -0.859, -0.269 | <0.001 | 0 | 0 | 0.55 |
| HCs | Negative | Number of errors | Guyer et al., 2007 | 0.476 | 0.116, 0.837 | 0.01 | 0 | 0 | 0.4 |
| HCs | Negative | Number of errors | McClure et al., 2005 | 0.448 | 0.156, 0.74 | 0.003 | 0 | 0 | 0.59 |
| HCs | Negative | Number of errors | Seymour et al., 2013 | 0.556 | 0.248, 0.864 | <0.001 | 0 | 0 | 0.67 |
| HCs | Positive | Accuracy | Bozorg et al., 2014 | -0.613 | -1.087, -0.14 | 0.011 | 57.82 | 0.1 | <0.1 |
| HCs | Positive | Accuracy | Khafif et al., 2023 | -0.697 | -1.059, -0.334 | <0.001 | 29.9 | 0.03 | 0.25 |
| HCs | Positive | Accuracy | Schenkel et al., 2012 | -0.415 | -0.724, -0.106 | 0.009 | 0 | 0 | 0.71 |
| HCs | Positive | Accuracy | Schenkel et al., 2020 | -0.591 | -1.06, -0.121 | 0.014 | 59.81 | 0.1 | <0.1 |
| HCs | Positive | Number of errors | Guyer et al., 2007 | 0.081 | -0.848, 1.01 | 0.86 | 84.67 | 0.38 | <0.1 |
| HCs | Positive | Number of errors | McClure et al., 2005 | 0.145 | -0.868, 1.157 | 0.78 | 91.1 | 0.49 | <0.1 |
| HCs | Positive | Number of errors | Seymour et al., 2013 | 0.624 | 0.315, 0.933 | <0.001 | 0 | 0 | 0.8 |
| Level III | | | | | | | | | |
| HCs | Anger | Accuracy | Bozorg et al., 2014 | -0.333 | -0.748, 0.082 | 0.12 | 46.98 | 0.06 | 0.15 |
| HCs | Anger | Accuracy | Khafif et al., 2023 | -0.574 | -0.874, -0.275 | <0.001 | 0 | 0 | 0.4 |
| HCs | Anger | Accuracy | Schenkel et al., 2012 | -0.326 | -0.739, 0.086 | 0.12 | 43.76 | 0.06 | 0.17 |
| HCs | Anger | Accuracy | Schenkel et al., 2020 | -0.48 | -0.93, -0.031 | 0.036 | 56.77 | 0.09 | 0.1 |
| HCs | Anger | Number of errors | Guyer et al., 2007 | 0.545 | 0.004, 1.086 | 0.048 | 54.07 | 0.08 | 0.14 |
| HCs | Anger | Number of errors | McClure et al., 2005 | 0.341 | 0.051, 0.632 | 0.021 | 0 | 0 | 0.79 |
| HCs | Anger | Number of errors | Seymour et al., 2013 | 0.558 | 0.107, 1.009 | 0.015 | 46.95 | 0.05 | 0.17 |
| HCs | Fear | Number of errors | Guyer et al., 2007 | 0.285 | -0.072, 0.642 | 0.12 | 0 | 0 | 0.57 |
| HCs | Fear | Number of errors | McClure et al., 2005 | 0.352 | 0.062, 0.643 | 0.018 | 0 | 0 | 0.41 |
| HCs | Fear | Number of errors | Seymour et al., 2013 | 0.435 | 0.129, 0.74 | 0.005 | 0 | 0 | 0.9 |
| HCs | Happiness | Accuracy | Bozorg et al., 2014 | -0.613 | -1.087, -0.14 | 0.011 | 57.82 | 0.1 | <0.1 |
| HCs | Happiness | Accuracy | Khafif et al., 2023 | -0.697 | -1.059, -0.334 | <0.001 | 29.9 | 0.03 | 0.25 |
| HCs | Happiness | Accuracy | Schenkel et al., 2012 | -0.415 | -0.724, -0.106 | 0.009 | 0 | 0 | 0.71 |
| HCs | Happiness | Accuracy | Schenkel et al., 2020 | -0.591 | -1.06, -0.121 | 0.014 | 59.81 | 0.1 | <0.1 |
| HCs | Happiness | Number of errors | Guyer et al., 2007 | 0.636 | 0.272, 1 | 0.001 | 0 | 0 | 0.74 |
| HCs | Happiness | Number of errors | McClure et al., 2005 | 0.665 | 0.37, 0.961 | <0.001 | 0 | 0 | 0.9 |
| HCs | Happiness | Number of errors | Seymour et al., 2013 | 0.624 | 0.315, 0.933 | <0.001 | 0 | 0 | 0.8 |
| HCs | Sadness | Accuracy | Bozorg et al., 2014 | -0.67 | -1.064, -0.276 | 0.001 | 0 | 0 | 0.73 |
| HCs | Sadness | Accuracy | Khafif et al., 2023 | -0.58 | -0.962, -0.198 | 0.003 | 0 | 0 | 0.46 |
| HCs | Sadness | Accuracy | Schenkel et al., 2020 | -0.523 | -0.895, -0.151 | 0.006 | 0 | 0 | 0.69 |
| HCs | Sadness | Number of errors | Guyer et al., 2007 | 0.647 | 0.283, 1.011 | <0.001 | 0 | 0 | 0.64 |
| HCs | Sadness | Number of errors | McClure et al., 2005 | 0.691 | 0.395, 0.987 | <0.001 | 0 | 0 | 0.54 |
| HCs | Sadness | Number of errors | Seymour et al., 2013 | 0.758 | 0.446, 1.07 | <0.001 | 0 | 0 | 0.96 |
| *Mixed age group* | | | | | | | | | |
| Level I | | | | | | | | | |
| HCs | Any emotion | Accuracy | Daros et al., 2014 | -0.078 | -0.335, 0.18 | 0.56 | 0 | 0 | 0.34 |
| HCs | Any emotion | Accuracy | Wiggins et al., 2017 | -0.261 | -0.891, 0.369 | 0.42 | 70.94 | 0.15 | <0.1 |
| HCs | Any emotion | Accuracy | Gray et al., 2024 | -0.392 | -0.754, -0.029 | 0.034 | 0 | 0 | 0.32 |
| Level II | | | | | | | | | |
| HCs | Negative | Accuracy | Daros et al., 2014 | 0.001 | -0.256, 0.258 | 0.99 | 0 | 0 | 0.51 |
| HCs | Negative | Accuracy | Wiggins et al., 2017 | -0.198 | -0.807, 0.411 | 0.52 | 69.25 | 0.14 | <0.1 |
| HCs | Negative | Accuracy | Gray et al., 2024 | -0.296 | -0.725, 0.132 | 0.17 | 25.65 | 0.03 | 0.25 |
| HCs | Positive | Accuracy | Daros et al., 2014 | -0.244 | -0.591, 0.103 | 0.17 | 39.72 | 0.03 | 0.2 |
| HCs | Positive | Accuracy | Wiggins et al., 2017 | -0.362 | -0.975, 0.252 | 0.25 | 69.1 | 0.14 | <0.1 |
| HCs | Positive | Accuracy | Gray et al., 2024 | -0.56 | -0.925, -0.194 | 0.003 | 0 | 0 | 0.48 |
| Level III | | | | | | | | | |
| HCs | Happiness | Accuracy | Daros et al., 2014 | -0.37 | -0.629, -0.111 | 0.005 | 0 | 0 | 0.62 |
| HCs | Happiness | Accuracy | Wiggins et al., 2017 | -0.442 | -0.808, -0.077 | 0.018 | 26.6 | 0.02 | 0.24 |
| HCs | Happiness | Accuracy | Gray et al., 2024 | -0.56 | -0.925, -0.194 | 0.003 | 0 | 0 | 0.48 |
| ***Discrimination*** | | | | | | | | | |
| *Adults* | | | | | | | | | |
| Level I | | | | | | | | | |
| HCs | Any emotion | Accuracy | Addington et al., 1998 | -0.552 | -0.738, -0.366 | <0.001 | 51.89 | 0.07 | <0.1 |
| HCs | Any emotion | Accuracy | Bellack et al., 1996 | -0.596 | -0.793, -0.4 | <0.001 | 59.07 | 0.09 | <0.1 |
| HCs | Any emotion | Accuracy | Benito et al., 2013 | -0.58 | -0.781, -0.378 | <0.001 | 58.4 | 0.09 | <0.1 |
| HCs | Any emotion | Accuracy | Bozikas et al., 2006 | -0.567 | -0.759, -0.374 | <0.001 | 56.47 | 0.08 | <0.1 |
| HCs | Any emotion | Accuracy | Burger et al., 2017 | -0.623 | -0.813, -0.433 | <0.001 | 53.87 | 0.07 | <0.1 |
| HCs | Any emotion | Accuracy | Darke et al., 2021 | -0.564 | -0.753, -0.375 | <0.001 | 55.66 | 0.08 | <0.1 |
| HCs | Any emotion | Accuracy | Foland-Ross et al., 2012 | -0.616 | -0.809, -0.424 | <0.001 | 56.11 | 0.08 | <0.1 |
| HCs | Any emotion | Accuracy | Getz et al., 2003 | -0.599 | -0.798, -0.4 | <0.001 | 59.09 | 0.09 | <0.1 |
| HCs | Any emotion | Accuracy | Vaskinn et al., 2007 | -0.626 | -0.811, -0.442 | <0.001 | 51.97 | 0.07 | <0.1 |
| HCs | Any emotion | Accuracy | Hulvershorn et al., 2012 | -0.608 | -0.808, -0.408 | <0.001 | 57.83 | 0.09 | <0.1 |
| HCs | Any emotion | Accuracy | Hwang et al., 2021 | -0.587 | -0.791, -0.384 | <0.001 | 58.82 | 0.09 | <0.1 |
| HCs | Any emotion | Accuracy | Ulusoy et al., 2020 | -0.553 | -0.738, -0.368 | <0.001 | 52.21 | 0.07 | <0.1 |
| HCs | Any emotion | Accuracy | Lahera et al., 2015 | -0.577 | -0.777, -0.376 | <0.001 | 58 | 0.09 | <0.1 |
| HCs | Any emotion | Accuracy | Li et al., 2022 | -0.572 | -0.769, -0.376 | <0.001 | 57.7 | 0.08 | <0.1 |
| HCs | Any emotion | Accuracy | Reddy et al., 2022 | -0.589 | -0.789, -0.388 | <0.001 | 59.29 | 0.09 | <0.1 |
| HCs | Any emotion | Accuracy | Rossell et al., 2014 | -0.63 | -0.816, -0.444 | <0.001 | 49.81 | 0.07 | <0.1 |
| HCs | Any emotion | Reaction time | Burger et al., 2017 | 0.187 | -0.072, 0.445 | 0.16 | 49.86 | 0.05 | <0.1 |
| HCs | Any emotion | Reaction time | Foland-Ross et al., 2012 | 0.239 | 0.016, 0.461 | 0.036 | 36.14 | 0.03 | 0.18 |
| HCs | Any emotion | Reaction time | Hulvershorn et al., 2012 | 0.2 | -0.058, 0.458 | 0.13 | 47.95 | 0.05 | <0.1 |
| HCs | Any emotion | Reaction time | Hwang et al., 2021 | 0.14 | -0.116, 0.397 | 0.28 | 45.76 | 0.05 | 0.1 |
| HCs | Any emotion | Reaction time | Li et al., 2022 | 0.189 | -0.065, 0.443 | 0.14 | 49.6 | 0.05 | <0.1 |
| HCs | Any emotion | Reaction time | Reddy et al., 2022 | 0.229 | -0.005, 0.462 | 0.06 | 40.44 | 0.03 | 0.14 |
| HCs | Any emotion | Reaction time | Rossell et al., 2014 | 0.106 | -0.084, 0.296 | 0.28 | 0 | 0 | 0.55 |
| Level II | | | | | | | | | |
| HCs | Negative | Accuracy | Burger et al., 2017 | -0.686 | -1.007, -0.364 | <0.001 | 30.97 | 0.03 | 0.22 |
| HCs | Negative | Accuracy | Darke et al., 2021 | -0.467 | -0.78, -0.154 | 0.003 | 40.34 | 0.04 | 0.17 |
| HCs | Negative | Accuracy | Hulvershorn et al., 2012 | -0.647 | -1.087, -0.207 | 0.004 | 60.88 | 0.12 | <0.1 |
| HCs | Negative | Accuracy | Li et al., 2022 | -0.499 | -0.882, -0.116 | 0.011 | 54.46 | 0.08 | <0.1 |
| HCs | Negative | Accuracy | Reddy et al., 2022 | -0.574 | -1.02, -0.128 | 0.012 | 65.65 | 0.13 | <0.1 |
| HCs | Negative | Reaction time | Burger et al., 2017 | 0.032 | -0.243, 0.307 | 0.82 | 0 | 0 | 0.8 |
| HCs | Negative | Reaction time | Hulvershorn et al., 2012 | 0.043 | -0.241, 0.328 | 0.76 | 0 | 0 | 0.78 |
| HCs | Negative | Reaction time | Li et al., 2022 | 0.035 | -0.231, 0.3 | 0.8 | 0 | 0 | 0.8 |
| HCs | Negative | Reaction time | Reddy et al., 2022 | 0.097 | -0.17, 0.364 | 0.48 | 0 | 0 | 0.99 |

## Sensitivity analyses, only good quality studies

**eTable n. 8 - Sensitivity analysis including only good quality studies**

Significant results are in **bold**.

The results are highlighted in red when the sensitivity analysis does not show significance, while the analysis considering all studies does.

| **Control group** | **Emotion type** | **Outcome type** | **Studies, n** | **BD patients, n** | **Controls, n** | **SMD** | **95% CIs** | **p-value** | **95% PIs** | **I^2^** | **tau^2^** | **Q test p-value** |
| --- | --- | --- | --- | --- | --- | --- | --- | --- | --- | --- | --- | --- |
| ***Identification*** | | | | | | | | | | | | |
| *Adults* | | | | | | | | | | | | |
| Level I | | | | | | | | | | | | |
| HCs | Any emotion | Accuracy | 43 | 2083 | 1776 | **-0.435** | **-0.543, -0.327** | **<0.001** | -0.962, 0.091 | 58.33 | 0.07 | <0.1 |
| HCs | Any emotion | Number of errors | 2 | 58 | 34 | 0.158 | -0.27, 0.586 | 0.47 | -0.27, 0.586 | 0 | 0 | 0.95 |
| HCs | Any emotion | Reaction time | 17 | 825 | 787 | **0.64** | **0.27, 1.009** | **0.001** | -0.822, 2.101 | 91.33 | 0.52 | <0.1 |
| FDRs | Any emotion | Accuracy | 8 | 496 | 274 | 0.058 | -0.097, 0.212 | 0.47 | -0.097, 0.212 | 0 | 0 | 0.69 |
| FDRs | Any emotion | Reaction time | 7 | 458 | 214 | 0.27 | -0.245, 0.785 | 0.3 | -1.09, 1.63 | 87.34 | 0.41 | <0.1 |
| Level II | | | | | | | | | | | | |
| HCs | Negative | Accuracy | 30 | 1540 | 1259 | **-0.318** | **-0.411, -0.226** | **<0.001** | **-0.568, -0.068** | 22.72 | 0.01 | <0.1 |
| HCs | Negative | Number of errors | 2 | 58 | 34 | 0.165 | -0.263, 0.593 | 0.45 | -0.263, 0.593 | 0 | 0 | 0.73 |
| HCs | Negative | Reaction time | 13 | 603 | 576 | **0.466** | **0.149, 0.783** | **0.004** | -0.591, 1.523 | 83.19 | 0.26 | <0.1 |
| HCs | Positive | Accuracy | 26 | 1386 | 1079 | **-0.195** | **-0.292, -0.098** | **<0.001** | -0.429, 0.04 | 19.93 | 0.01 | <0.1 |
| HCs | Positive | Reaction time | 8 | 433 | 401 | **0.593** | **0.182, 1.005** | **0.005** | -0.513, 1.7 | 83.42 | 0.27 | <0.1 |
| FDRs | Negative | Accuracy | 7 | 475 | 253 | 0.042 | -0.117, 0.202 | 0.6 | -0.117, 0.202 | 0 | 0 | 0.64 |
| FDRs | Negative | Reaction time | 6 | 437 | 193 | 0.295 | -0.312, 0.901 | 0.34 | -1.227, 1.816 | 89.93 | 0.51 | <0.1 |
| FDRs | Positive | Accuracy | 4 | 363 | 182 | -0.015 | -0.204, 0.174 | 0.87 | -0.204, 0.174 | 0 | 0 | 0.37 |
| FDRs | Positive | Reaction time | 3 | 325 | 122 | 0.095 | -0.16, 0.35 | 0.47 | -0.229, 0.419 | 16.91 | 0.01 | 0.37 |
| Level III | | | | | | | | | | | | |
| HCs | Anger | Accuracy | 23 | 1305 | 1016 | **-0.28** | **-0.388, -0.172** | **<0.001** | -0.573, 0.013 | 30.08 | 0.02 | 0.1 |
| HCs | Anger | Reaction time | 7 | 425 | 366 | 0.5 | -0.001, 1.001 | 0.05 | -0.808, 1.809 | 88.11 | 0.38 | <0.1 |
| HCs | Disgust | Accuracy | 16 | 993 | 750 | **-0.27** | **-0.429, -0.112** | **0.001** | -0.741, 0.201 | 54.15 | 0.05 | <0.1 |
| HCs | Disgust | Number of errors | 2 | 58 | 34 | 0.343 | -0.088, 0.774 | 0.12 | -0.088, 0.774 | 0 | 0 | 0.56 |
| HCs | Disgust | Reaction time | 5 | 380 | 299 | 0.2 | -0.125, 0.526 | 0.23 | -0.435, 0.835 | 62.19 | 0.08 | <0.1 |
| HCs | Fear | Accuracy | 24 | 1328 | 1045 | **-0.303** | **-0.424, -0.183** | **<0.001** | -0.695, 0.088 | 44 | 0.04 | <0.1 |
| HCs | Fear | Number of errors | 2 | 58 | 34 | -0.002 | -0.431, 0.426 | 0.99 | -0.431, 0.426 | 0 | 0 | 0.33 |
| HCs | Fear | Reaction time | 8 | 442 | 415 | **0.328** | **0.124, 0.533** | **0.002** | -0.065, 0.721 | 36.79 | 0.03 | 0.18 |
| HCs | Happiness | Accuracy | 23 | 1308 | 994 | **-0.152** | **-0.244, -0.06** | **0.001** | -0.315, 0.01 | 9.48 | 0 | 0.2 |
| HCs | Happiness | Reaction time | 7 | 407 | 375 | **0.695** | **0.241, 1.148** | **0.003** | -0.465, 1.854 | 84.56 | 0.3 | <0.1 |
| HCs | Neutral | Accuracy | 12 | 580 | 510 | -0.096 | -0.216, 0.025 | 0.12 | -0.216, 0.025 | 0 | 0 | 0.59 |
| HCs | Neutral | Number of errors | 2 | 58 | 34 | 0.028 | -0.4, 0.456 | 0.9 | -0.4, 0.456 | 0 | 0 | 0.49 |
| HCs | Neutral | Reaction time | 8 | 449 | 404 | **0.238** | **0.012, 0.463** | **0.039** | -0.232, 0.707 | 46.59 | 0.04 | <0.1 |
| HCs | Sadness | Accuracy | 25 | 1363 | 1059 | **-0.205** | **-0.288, -0.122** | **<0.001** | **-0.288, -0.122** | 0 | 0 | 0.65 |
| HCs | Sadness | Reaction time | 6 | 409 | 346 | 0.154 | -0.086, 0.394 | 0.21 | -0.297, 0.605 | 45.53 | 0.04 | 0.1 |
| HCs | Surprise | Accuracy | 11 | 636 | 534 | **-0.288** | **-0.406, -0.17** | **<0.001** | **-0.406, -0.17** | 0.01 | 0 | 0.66 |
| HCs | Surprise | Reaction time | 2 | 286 | 210 | 0.229 | -0.137, 0.596 | 0.22 | -0.298, 0.757 | 39.88 | 0.04 | 0.2 |
| FDRs | Anger | Accuracy | 4 | 363 | 182 | 0.162 | -0.118, 0.443 | 0.26 | -0.303, 0.628 | 44.43 | 0.04 | 0.15 |
| FDRs | Anger | Reaction time | 3 | 325 | 122 | -0.009 | -0.222, 0.204 | 0.94 | -0.222, 0.204 | 0 | 0 | 0.99 |
| FDRs | Disgust | Accuracy | 2 | 302 | 100 | -0.199 | -1.135, 0.738 | 0.68 | -1.768, 1.371 | 90.11 | 0.41 | <0.1 |
| FDRs | Disgust | Reaction time | 2 | 302 | 100 | 0.013 | -0.731, 0.758 | 0.97 | -1.213, 1.24 | 84.88 | 0.25 | <0.1 |
| FDRs | Fear | Accuracy | 5 | 404 | 207 | 0.057 | -0.119, 0.234 | 0.52 | -0.119, 0.234 | 0 | 0 | 0.35 |
| FDRs | Fear | Reaction time | 4 | 366 | 147 | 0.045 | -0.36, 0.449 | 0.83 | -0.73, 0.82 | 68.98 | 0.11 | <0.1 |
| FDRs | Happiness | Accuracy | 4 | 363 | 182 | -0.225 | -0.546, 0.097 | 0.17 | -0.801, 0.352 | 56.87 | 0.06 | <0.1 |
| FDRs | Happiness | Reaction time | 3 | 325 | 122 | 0.087 | -0.213, 0.387 | 0.57 | -0.346, 0.521 | 33.32 | 0.03 | 0.26 |
| FDRs | Neutral | Accuracy | 3 | 325 | 122 | 0.045 | -0.168, 0.258 | 0.68 | -0.168, 0.258 | 0 | 0 | 0.7 |
| FDRs | Neutral | Reaction time | 3 | 325 | 122 | -0.206 | -0.42, 0.007 | 0.06 | -0.42, 0.007 | 0 | 0 | 0.68 |
| FDRs | Sadness | Accuracy | 4 | 363 | 182 | 0.011 | -0.177, 0.2 | 0.91 | -0.177, 0.2 | 0 | 0 | 0.76 |
| FDRs | Sadness | Reaction time | 3 | 325 | 122 | -0.181 | -0.395, 0.032 | 0.1 | -0.395, 0.032 | 0 | 0 | 0.88 |
| FDRs | Surprise | Accuracy | 2 | 304 | 136 | 0.041 | -0.218, 0.3 | 0.76 | -0.279, 0.361 | 23.46 | 0.01 | 0.25 |
| *Children/adolescents* | | | | | | | | | | | | |
| Level I | | | | | | | | | | | | |
| HCs | Any emotion | Accuracy | 4 | 116 | 108 | **-0.717** | **-0.988, -0.446** | **<0.001** | **-0.988, -0.446** | 0 | 0 | 0.77 |
| HCs | Any emotion | Number of errors | 4 | 162 | 231 | **0.659** | **0.447, 0.87** | **<0.001** | **0.447, 0.87** | 0 | 0 | 0.89 |
| Level II | | | | | | | | | | | | |
| HCs | Negative | Accuracy | 3 | 94 | 86 | **-0.611** | **-0.911, -0.311** | **<0.001** | **-0.911, -0.311** | 0 | 0 | 0.8 |
| HCs | Negative | Number of errors | 3 | 110 | 153 | **0.493** | **0.235, 0.751** | **<0.001** | **0.235, 0.751** | 0 | 0 | 0.7 |
| HCs | Positive | Accuracy | 3 | 94 | 86 | **-0.697** | **-1.059, -0.334** | **<0.001** | **-1.196, -0.197** | 29.9 | 0.03 | 0.25 |
| HCs | Positive | Number of errors | 3 | 110 | 153 | 0.281 | -0.371, 0.933 | 0.4 | -0.936, 1.498 | 83.22 | 0.27 | <0.1 |
| Level III | | | | | | | | | | | | |
| HCs | Anger | Accuracy | 3 | 94 | 86 | **-0.574** | **-0.874, -0.275** | **<0.001** | **-0.874, -0.275** | 0 | 0 | 0.4 |
| HCs | Anger | Number of errors | 3 | 110 | 153 | **0.447** | **0.189, 0.705** | **0.001** | **0.189, 0.705** | 0 | 0 | 0.29 |
| HCs | Fear | Number of errors | 3 | 110 | 153 | **0.364** | **0.107, 0.621** | **0.005** | **0.107, 0.621** | 0 | 0 | 0.7 |
| HCs | Happiness | Accuracy | 3 | 94 | 86 | **-0.697** | **-1.059, -0.334** | **<0.001** | **-1.196, -0.197** | 29.9 | 0.03 | 0.25 |
| HCs | Happiness | Number of errors | 3 | 110 | 153 | **0.643** | **0.382, 0.904** | **<0.001** | **0.382, 0.904** | 0 | 0 | 0.94 |
| HCs | Sadness | Accuracy | 2 | 55 | 55 | **-0.58** | **-0.962, -0.198** | **0.003** | **-0.962, -0.198** | 0 | 0 | 0.46 |
| HCs | Sadness | Number of errors | 3 | 110 | 153 | **0.703** | **0.442, 0.965** | **<0.001** | **0.442, 0.965** | 0 | 0 | 0.82 |
| ***Discrimination*** | | | | | | | | | | | | |
| *Adults* | | | | | | | | | | | | |
| Level I | | | | | | | | | | | | |
| HCs | Any emotion | Accuracy | 11 | 361 | 336 | **-0.522** | **-0.741, -0.303** | **<0.001** | -1.067, 0.024 | 48.32 | 0.06 | <0.1 |
| HCs | Any emotion | Reaction time | 6 | 247 | 201 | 0.106 | -0.084, 0.296 | 0.28 | -0.084, 0.296 | 0 | 0 | 0.55 |
| FDRs | Any emotion | Accuracy | 2 | 68 | 81 | -0.04 | -0.742, 0.661 | 0.91 | -1.153, 1.073 | 75.52 | 0.19 | <0.1 |
| Level II | | | | | | | | | | | | |
| HCs | Negative | Accuracy | 4 | 170 | 125 | **-0.467** | **-0.78, -0.154** | **0.003** | -0.973, 0.038 | 40.34 | 0.04 | 0.17 |
| HCs | Negative | Reaction time | 4 | 170 | 125 | 0.052 | -0.184, 0.288 | 0.66 | -0.184, 0.288 | 0 | 0 | 0.92 |

## Sensitivity analyses, removing those studies whose lower-level data were calculated from higher-level information

**eTable n. 9 - Sensitivity analysis removing those studies whose lower-level data were calculated from higher-level information**

Significant results are in **bold**.

The results are highlighted in red when the sensitivity analysis does not show significance, while the analysis considering all studies does.

| **Control group** | **Emotion type** | **Outcome type** | **Studies, n** | **BD patients, n** | **Controls, n** | **SMD** | **95% CIs** | **p-value** | **95% PIs** | **I^2^** | **tau^2^** | **Q test p-value** |
| --- | --- | --- | --- | --- | --- | --- | --- | --- | --- | --- | --- | --- |
| ***Identification*** | | | | | | | | | | | | |
| *Adults* | | | | | | | | | | | | |
| Level I | | | | | | | | | | | | |
| HCs | Any emotion | Accuracy | 38 (from 65) | 2093 | 2107 | **-0.587** | **-0.712, -0.462** | **<0.001** | -1.216, 0.041 | 70.95 | 0.1 | <0.1 |
| HCs | Any emotion | Reaction time | 11 (from 25) | 922 | 1032 | **0.652** | **0.153, 1.15** | **0.01** | -1.005, 2.308 | 95.89 | 0.65 | <0.1 |
| FDRs | Any emotion | Accuracy | 5 (from 11) | 614 | 468 | 0.034 | -0.185, 0.253 | 0.76 | -0.371, 0.438 | 52.91 | 0.03 | <0.1 |
| FDRs | Any emotion | Reaction time | 4 (from 9) | 576 | 408 | 0.491 | -0.293, 1.274 | 0.22 | -1.207, 2.189 | 95.71 | 0.59 | <0.1 |
| Level II | | | | | | | | | | | | |
| HCs | Negative | Accuracy | 7 (from 45) | 496 | 492 | **-0.47** | **-0.816, -0.123** | **0.008** | -1.33, 0.39 | 81.3 | 0.16 | <0.1 |
| HCs | Negative | Reaction time | 4 (from 19) | 152 | 195 | 0.636 | -0.408, 1.681 | 0.23 | -1.628, 2.901 | 94.32 | 1.05 | <0.1 |
| HCs | Positive | Accuracy | 5 (from 39) | 421 | 422 | -0.147 | -0.68, 0.386 | 0.59 | -1.359, 1.065 | 90.18 | 0.31 | <0.1 |
| HCs | Positive | Reaction time | 3 (from 13) | 122 | 165 | 0.793 | -0.451, 2.037 | 0.21 | -1.626, 3.212 | 94.2 | 1.12 | <0.1 |
| *Children/adolescents* | | | | | | | | | | | | |
| Level I | | | | | | | | | | | | |
| HCs | Any emotion | Accuracy | 4 (from 6) | 87 | 87 | **-0.579** | **-0.883, -0.274** | **<0.001** | -0.883, -0.274 | 0.01 | 0 | 0.46 |
| HCs | Any emotion | Reaction time | 2 (from 3) | 40 | 40 | 0.352 | -0.298, 1.003 | 0.29 | -0.554, 1.259 | 43.39 | 0.1 | 0.18 |
| *Mixed age groups* | | | | | | | | | | | | |
| Level I | | | | | | | | | | | | |
| HCs | Any emotion | Accuracy | 2 (from 3) | 144 | 102 | -0.078 | -0.335, 0.18 | 0.56 | -0.335, 0.18 | 0 | 0 | 0.34 |
| ***Discrimination*** | | | | | | | | | | | | |
| *Adults* | | | | | | | | | | | | |
| Level I | | | | | | | | | | | | |
| HCs | Any emotion | Accuracy | 12 (from 16) | 379 | 481 | **-0.63** | **-0.863, -0.397** | **<0.001** | -1.292, 0.031 | 61.17 | 0.1 | <0.1 |
| HCs | Any emotion | Reaction time | 3 (from 7) | 120 | 188 | 0.305 | -0.143, 0.753 | 0.18 | -0.48, 1.09 | 69.74 | 0.11 | <0.1 |
| Level II | | | | | | | | | | | | |
| HCs | Negative | Accuracy | 3 (from 5) | 134 | 89 | **-0.584** | **-0.882, -0.285** | **<0.001** | **-0.927, -0.24** | 10.7 | 0.01 | 0.34 |
| HCs | Negative | Reaction time | 3 (from 4) | 134 | 89 | 0.032 | -0.243, 0.307 | 0.82 | -0.243, 0.307 | 0 | 0 | 0.8 |

## Publication bias

The funnel plots for each meta-analysis are available at https://osf.io/ewx8z/?view_only=a44a50eb883a4bfbabb6be7a9d3a70a9

**eTable n. 10 - Publication bias**

| **Control group** | **Emotion type** | **Outcome type** | **Egger's z** | **p-value** |
| --- | --- | --- | --- | --- |
| ***Identification*** | | | | |
| *Adults* | | | | |
| Level I | | | | |
| HCs | Any emotion | Accuracy | -2.76 | **0.006** |
| HCs | Any emotion | Reaction time | 3.492 | **<0.001** |
| FDRs | Any emotion | Accuracy | -0.171 | 0.86 |
| Level II | | | | |
| HCs | Negative | Accuracy | -4.103 | **<0.001** |
| HCs | Negative | Reaction time | 2.28 | **0.023** |
| HCs | Positive | Accuracy | 0.19 | 0.85 |
| HCs | Positive | Reaction time | 1.379 | 0.17 |
| FDRs | Negative | Accuracy | -0.546 | 0.58 |
| Level III | | | | |
| HCs | Anger | Accuracy | -2.981 | **0.003** |
| HCs | Anger | Reaction time | 2.804 | **0.005** |
| HCs | Disgust | Accuracy | -0.391 | 0.7 |
| HCs | Fear | Accuracy | -3.219 | **0.001** |
| HCs | Fear | Reaction time | 1.326 | 0.18 |
| HCs | Happiness | Accuracy | -2.854 | **0.004** |
| HCs | Happiness | Reaction time | 2.537 | **0.011** |
| HCs | Neutr al | Accuracy | 0.494 | 0.62 |
| HCs | Neutral | Reaction time | 1.055 | 0.29 |
| HCs | Sadness | Accuracy | -2.424 | **0.015** |
| HCs | Sadness | Reaction time | 1.28 | 0.2 |
| HCs | Surprise | Accuracy | -1.526 | 0.13 |
| ***Discrimination*** | | | | |
| *Adults* | | | | |
| Level I | | | | |
| HCs | Any emotion | Accuracy | -0.866 | 0.39 |
